# The first draft reference genome of the American mink (*Neovison vison*)

**DOI:** 10.1038/s41598-017-15169-z

**Published:** 2017-11-06

**Authors:** Zexi Cai, Bent Petersen, Goutam Sahana, Lone B. Madsen, Knud Larsen, Bo Thomsen, Christian Bendixen, Mogens Sandø Lund, Bernt Guldbrandtsen, Frank Panitz

**Affiliations:** 1 Center for Quantitative Genetics and Genomics, Department of Molecular Biology and Genetics, Aarhus University, DK-8830 Tjele, Denmark; 2 DTU Bioinformatics, Department of Bio and Health Informatics, Technical University of Denmark, 2800 Kgs, Lyngby, Denmark; 3 Section for Molecular Genetics and Systems Biology, Department of Molecular Biology and Genetics, Aarhus University, DK-8830 Tjele, Denmark; 4 Centre of Excellence for Omics-Driven Computational Biodiscovery (COMBio), Faculty of Applied Sciences, AIMST University, Kedah, Malaysia

## Abstract

The American mink (*Neovison vison*) is a semiaquatic species of mustelid native to North America. It’s an important animal for the fur industry. Many efforts have been made to locate genes influencing fur quality and color, but this search has been impeded by the lack of a reference genome. Here we present the first draft genome of mink. In our study, two mink individuals were sequenced by Illumina sequencing with 797 Gb sequence generated. Assembly yielded 7,175 scaffolds with an N50 of 6.3 Mb and length of 2.4 Gb including gaps. Repeat sequences constitute around 31% of the genome, which is lower than for dog and cat genomes. The alignments of mink, ferret and dog genomes help to illustrate the chromosomes rearrangement. Gene annotation identified 21,053 protein-coding sequences present in mink genome. The reference genome’s structure is consistent with the microsatellite-based genetic map. Mapping of well-studied genes known to be involved in coat quality and coat color, and previously located fur quality QTL provide new knowledge about putative candidate genes for fur traits. The draft genome shows great potential to facilitate genomic research towards improved breeding for high fur quality animals and strengthen our understanding on evolution of Carnivora.

## Introduction

American mink (*Neovison vison*, 2n = 30) is a semiaquatic species of mustelids native to North America. The exact taxonomic position of the mink remains to be resolved^1^. So far data from cytogenetics^2^ and molecular phylogeny^3^ placed American mink and sea mink (*Neovison macrodon*) to a separate genus *Neovison*. Since the recent extinction of the sea mink^4^, the American mink is the only extant member of the genus *Neovison* in the order Carnivora. The estimated genome size of mink is 2.7 Gb similar to the ferret genome^5^. Besides, American mink has the smallest number of chromosomes among the Carnivora^6^. Because of this, mink was included in many fluorescent *in situ* hybridization of a chromosome-specific probe from one species to chromosomes from other species (ZOO-FISH) analyses^7–9^.

American mink is the most common farmed animal for fur, exceeding the silver fox, sable, marten, and skunk in economic importance. Fur quality, fur color and fertility are the most important traits for the fur industry. Considerable genetic research has been done in order to uncover genes causing variation in these traits. The first linkage map based on microsatellite markers was published in 2007^10^. This map was updated twice by incorporating more markers and re-assigning markers order^11,12^, yielding a total of 104 markers. Quantitative Trait Locus (QTL) mapping based on linkage analyses mapped some genes related to fur quality and fur color to chromosomes^13^. Comparative mapping identified 16 candidate genes for fur quality or color and were mapped to chromosome arms^12^. Sequencing of bacterial artificial chromosome (BAC) clones based on hybridization was used to sequence these genes^5^. Also, a transcriptome profile of mink is available^14^. However, without a reference genome, prospects to find genes for economical important traits of interest to selective breeding remain limited.

With the first release of the human genome^15^ and the emergence of the next generation sequencing technology^16^, more and more genome sequences have become available. Even though we already have dog^17^, cat^18^ and ferret^19^ genomes, the divergent^20^ and chromosome rearrangement^21^ of these species, it will be valuable to obtain the mink genome sequence. With a reference genome assembly for mink available, research and breeding based on genomic approaches will become possible. For example, gene function and expression analysis with RNA-seq (RNA sequencing)^22^, epigenetics and transcription factor analysis with ChIP-seq (chromatin immunoprecipitation sequencing)^23^ and whole genome genotyping all depend on a reference genome. For genotyping, availability of a high quality reference greatly eases discovery and evaluation of single nucleotide polymorphisms (SNP), which will make genome-wide association studies (GWAS) possible^24^. Also, genomic selection^25^ which has been extremely successful in several farm animal species is greatly facilitated by easy and reliable marker discovery. In order to provide the fundamental information for genomic research for fur related traits, we present the first draft genome sequence of American mink, and show the application potential for fur industry.

## Results

### Sequencing and assembly

Whole genome shotgun sequencing (WGS) strategy was used to generate data from two individuals of American minks, one pearl male and one brown female. 383 Gb next-generation Illumina paired-end (PE) reads was generated by sequencing genome shotgun libraries with insert sizes of 150, 165 and 600 bp. In addition, 414 Gb of mate-paired (MP) reads were generated with insert sizes of 3, 5, 6, 8, 10, 14 and 32 kb. The estimated size of the mink genome of 2.7 Gb was covered 295 fold. Sequencing reads were assembled by ALLPATHS-LG^26^ with three different combinations of data (Supplementary Table S2) due to server memory limitations (1TB memory available). We constructed four assemblies. The first one was the Pearl-mink-assembly (PMA) constructed using reads from the pearl mink. The second one was Brown-mink-assembly (BMA) using part of brown mink data (for details see method section). The third one was a hybrid-assembly (HA) of all pearl mink data and part of brown mink data (part of 150 bp, part of 600 bp, 8 kb, part of 10 kb, 14 kb and 32 kb libraries were used). The fourth assembly, draft-assembly, was constructed by an additional round of scaffolding with SSPACE^27^ using the HA (lowest number of scaffolds) as the backbone and simulated long insert MP reads from BMA (longer scaffold N50) using ART^28^. Finally this resulted in a 2.45 Gb assembly including gaps (Table 1), which corresponds to 90% of the estimated 2.7 Gb mink genome size^29^. The draft genome consisted of 7,175 scaffolds with an N50 of 6.3 Mb, where the largest scaffold was 40.3 Mb. The detailed information on all the four assemblies was presented in Supplementary Table S2. We experienced that the draft assembly, which combined HA with additional scaffolding procedure largely improved the scaffold N50 of assembly and reduced the number of scaffolds.

**Table 1:** Summary details of the mink genome assembly.

*Neovison vison* genome	
Estimated genome size	2.7 Gb
Total assembly length	2.45 Gb
Total sequence	2.27 Gb
Short read coverage	295
Scaffold count	7,175
Scaffold N50	6.3 Mb

### Assembly assessment

To assess the quality of the draft genome and the validity of the additional scaffolding procedure, we first mapped a part of the PE and MP sequence data back to the assembly. Insert size libraries of 150 bp, 600 bp and 3 kb were chosen to assess the quality of the assembly by aligning them to the four assemblies (Supplementary Table S3). The result showed that 98%, 98% and 91% of these libraries could be mapped to the draft assembly. In addition, 95.72%, 89.55% and 69.91% of pairs were properly paired (Supplementary Table S3). Compared draft assembly with the PMA, the percentage of alignment increased 0.04%, 0.04% and 0.11%, but properly pair decreased 0.23%, 0.11% and 0.47%. The decreased of properly pair mostly came from the HA, since the HA was built from reads from both individuals. After additional scaffolding, the properly pair for 150 bp and 600 bp libraries were the same in the draft assembly and the HA, and the properly pair for 3 kb library increased 0.42% from the HA. In all, the sequence alignment of these three libraries showed similar results among different assemblies and improvement of the additional scaffolding. However, because the tolerance of heterozygosity of two individuals, some alignments decrease by a small percentage from PMA to HA.

For the two BAC-end sequence libraries, after filtering out clones where it was not possible to find both ends, we had 1,795 pairs of BES (BAC-end sequence) from black mink^30^ and 833 pairs BES from the CHORI-231 BAC library^5^. The BESs were mapped to the genome assembly. To estimate the properly paired reads for BES libraries, both ends should be uniquely aligned to the same scaffolds. Out of these, 99.32% of the reads from the first library and 89.7% of the reads from the second library could be mapped to the draft assembly. From the first library, 79.55% (1,428 out of 1,795) pairs and 55.58% (463 out of 833) of pairs from the second library were properly paired. The estimated insert sizes were close to the sizes targeted during the construction of the BAC libraries, which were 25 kb (20–50 kb BAC library) and 164 kb (170 kb BAC library) (Supplementary Table S3). Comparing the BES alignment of draft assembly with PMA and HA, we found the properly paired increased from 76.27% to 78.27% and at last to 79.55% for the first BES library, and increased from 40.58% to 50.54% and at last to 55.58% for the second BES library. This showed our strategy successfully solved some long distance linking.

The similar short reads mapping pattern was also presented in ferret assembly. We downloaded and aligned selected libraries of the ferret assembly (Supplementary Table S4). The 180 bp library had 88.93% of reads that could be aligned, and 79.20% of them were properly paired. The 3 kb library had 93.15% reads aligned and 80.64% paired properly. The 6 kb-10 kb and 40 kb libraries had 95.90% and 86.42% reads aligned and 82.06% and 63.77% properly paired, respectively. The poor properly paired reads of the BES libraries of mink and 40 kb library of the ferret assembly came mostly from two ends aligned to different scaffolds (Supplementary Table S3 and Supplementary Table S4). These comparisons showed that we had a comparable genome assembly for mink similar to ferret.

The completeness of the genes represented in the assembly was assessed by BUSCO version 2^31^. The mammalian orthologous gene set (4,104 genes) was used to assess the presence of standard genes in the genome assembly. The same analysis was also conducted with single-individual (Pearl mink and Brown mink) assembly and ferret genome. Results showed that 98% (95.8% complete and 2.2% fragmentary) of these 4,104 genes could be detected in the draft mink assembly (Supplementary Table S5). We could see the final scaffolding using the simulated mate-pair data improved the complete from 93.80% (PMA) and 94.9% (BMA) to 95.80%, fragment genes decreased from 3.8% and 2.7% to 2.2% and missing genes decreased from 2.4% (PMA and BMA) to 2.0% (Supplementary Table S5). The results from the draft assembly were comparable with that of ferret and showed that our strategy to build the draft assembly improved the assembly of coding sequence regions. Moreover, we also checked the 45S rDNA (ribosomal DNA) completeness in the assembly, as other species whose genome were built from second-generation sequencing; we also could not detect the complete 45S rDNA in the assembly^32^. This mostly came from the difficulty to assemble tandem repeat cluster from short reads^33^.

### Repeat annotation in the mink genome and comparison to related species

**Figure 1:**
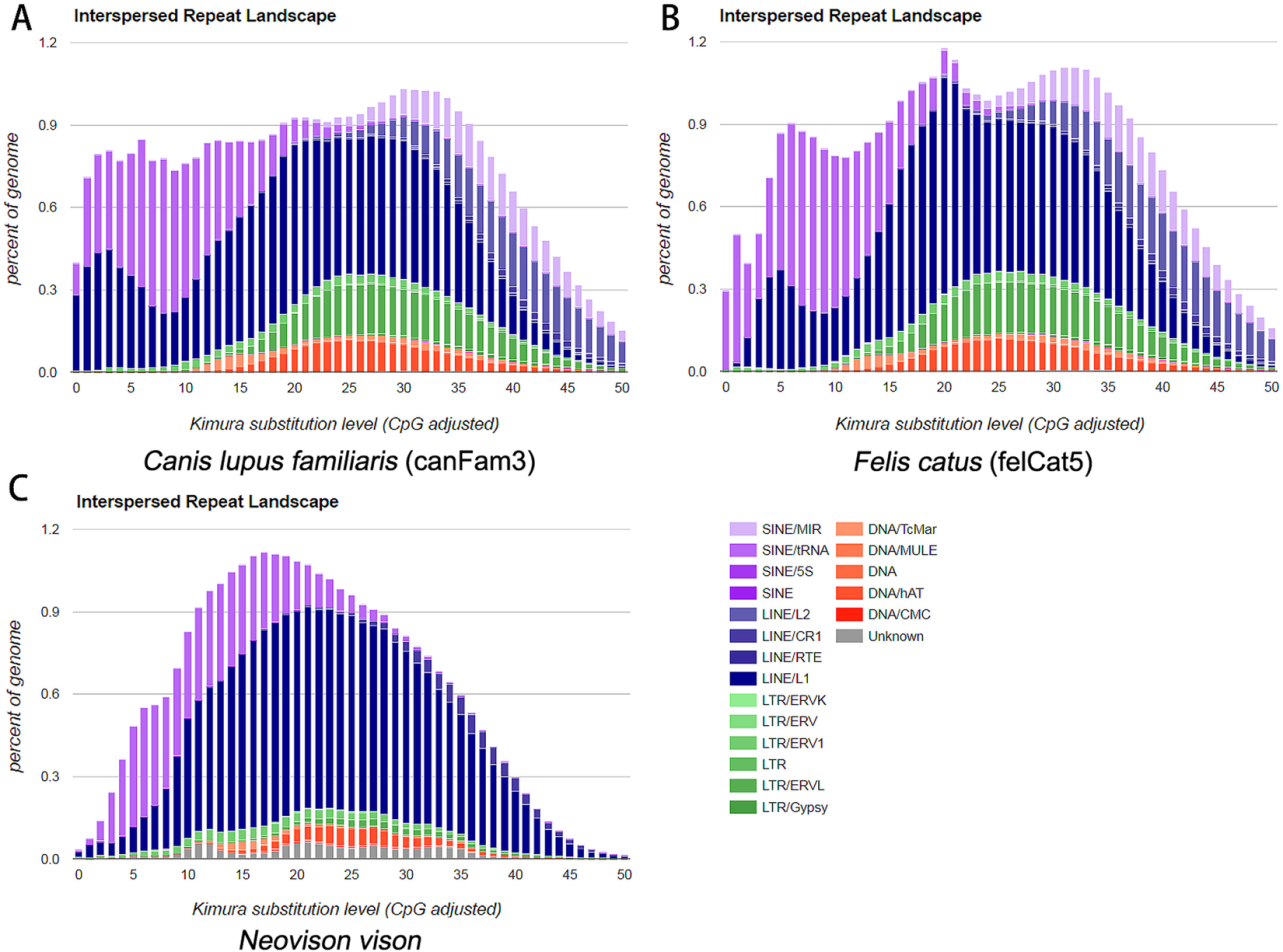
Interspersed Repeat Landscape of the (**A**) dog; (**B**) cat and (**C**) mink genome.

RepeatModeler^34^ was used to construct the species-specific repeat library for mink and RepeatExplorer^35^ was used to analyze the identified repeat families. RepeatExplorer identified three novel satellite repeats in mink. One of these satellites was similar to the *Mustela putorius* 1080 bp *Bam* HI repeat DNA (GenBank: x59440.1). Satellite repeats constituted around 2.26% of the total mink genome based on the result of RepeatExplorer (Supplementary Figure S1). RepeatModeler identified 285 repeat consensuses in mink. Among these, LINE (long interspersed nuclear elements) and LTR (long terminal repeat) had the highest number of family members (Supplementary Table S6). Combining the output of these two analyses and a repeat database of the dog genome^36^, we built a comprehensive mink repeat database for RepeatMasker. At the end, 29% of the mink genome was found to be composed of repeat sequences (Table 2). Adding the low represented satellite repeats sequence from the RepeatMasker result, we had around 31% of mink genome sequence belonging to repeat sequence. Our assembly have 10% less sequence than estimated genome size and part of these sequences probably are repeat sequence, so the real repeat content of mink might be larger than we observed now. The dog genome and cat genome have 43% and 44% of repeat sequence (http://www.repeatmasker.org) according to the pre-analysis genome from RepeatMasker website. Since there are still some part of repeat elements that are missing in dog and cat assembly^37,38^, the repeat content will be slightly higher than 43% for dog and 44% for cat. Therefore, we believe mink have less repeat content than dog and cat. The most abundant repeat family LINEs is shared by these three genomes: around 20% of the dog genome, 21% of the cat genome and 15% of the mink genome consist of LINEs. In all three species, the second most abundant repeat family is short interspersed nuclear elements (SINE). These constitute 11%, 11% and 7% of the genomes respectively. The dynamics of the repeat sequence is largely shaping the genome^39^. Therefore, we computed the substitution level of repeat sequences in these three species by aligning all elements belonging to the same family to consensus sequences in each species. As shown in Fig. 1, the dog and cat genomes share one major peak of substitution level at 30–35. Dog has another small substitution level peak around 0–10, while cat has a small substitution level peak around 5–10. In contrast, the mink assembly has only one substitution level peak around 10–25. Moreover, the peaks for LINEs and SINEs are similar in dog and cat. In dog and cat, the peak for LINEs are at 5, 20 and 30–35. For SINEs, there is a consistence pattern with LINEs in these two species. However, the peak for LINEs and SINEs are different in mink genome. The LINEs in mink has a peak at around 20–25. The SINEs in mink has a peak at around 10–20. These results showed that more repeat sequences in dog and cat have high identity compared to repeat families’ consensus sequences. This implied that dog and cat have more recently active repeat sequences, especially LINEs and SINEs. This could partially explains why dog and cat genomes have higher amounts of repeat sequence than mink genome, because dog and cat genome have more recently inserted repeat sequences compared to the mink genome.

**Table 2:** The repeat sequence composition of mink genome.

Family	Percent of genome
LINEs	14.76
SINEs	7.05
LTR elements	3.35
Small RNA	2.39
Transposon	1.29
Unclassified	1.19
Simple repeats	0.70
Low complexity	0.40
Satellites	0.09
Total	28.85

### Alignment with the dog genome assembly

**Figure 2:**
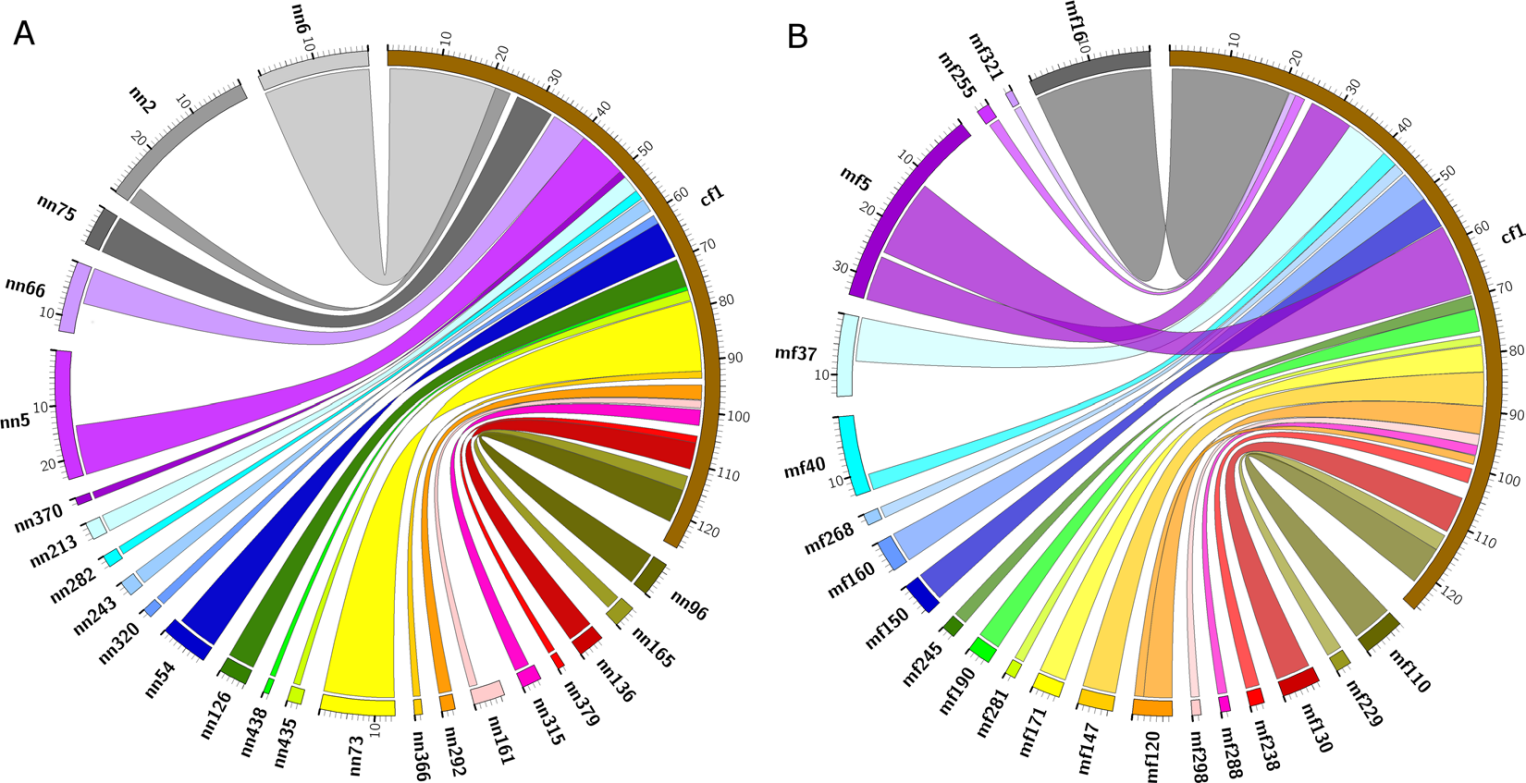
Genome alignment of (**A**) mink genome and (**B**) ferret genome to dog chromosome 1 (cf1). The scaffold of mink start with ‘nn’ and ferret scaffold srart with ‘mf’. The first seven Mb of mink scaffold 66 (nn66) and ferret scaffold 37 (mf37) can be aligned to position 33 Mb to 40 Mb of dog chromosome 1 (cf1).

The mink and the ferret genome assemblies were aligned to the dog genome assembly and similar alignment patterns were observed (Fig. 2A for mink, Fig. 2B for ferret and Supplementary Figures S2–S38). A total of 1,525,079,306 bp of the mink genome assembly and 1,516,552,860 bp of the ferret genome assembly could be aligned to the dog genome. The distributions of synteny block sizes were similar for the two species (Table 3). Both the mink and the ferret assemblies shared a number of chromosomal rearrangements when compared to the dog genome (Table 4, Fig. 2 and Supplementary Figures S2–S38). For example, scaffold 66 (nn66) of mink and scaffold 37 (mf37) of ferret could be aligned to dog chromosome 1 (Fig. 2) and dog chromosome 12 (Supplementary Figure S12). Likewise part of nn4 and mf7 could be aligned to part of dog chromosome 14, whereas other part of nn4 and mf7 could be aligned to the reversed sequence of other part of dog chromosome 14 (Supplementary Figure S14). First, these findings demonstrated that the scaffold structure is consistent between the mink and the ferret, some of the same rearrangements were detected when comparing to the dog assembly. Second, they identified specific rearrangements that must have happened since the last common ancestor of the three species and either the current dog population or the last common ancestor of mink and ferret.

**Table 3:** The size distribution of synteny block.

Block size	Count of each block size in mink	Total length for each block size (bp)	Count of each block size in ferret	Total length for each block size (bp)
10 bp–100 bp	205	15,162	159	11,219
100 bp–1 kb	1889	772,514	1615	698,172
1 kb–10 kb	1259	3,903,496	1227	3,703,896
10 kb–100 kb	414	15,469,913	323	10,698,371
100 kb–1 Mb	402	167,495,661	222	91,195,403
1 Mb–10 Mb	364	1,162,951,190	300	1,076,777,135
10 Mb–100 Mb	13	174,471,370	24	333,468,664
Total		1,525,079,306		1,516,552,860

**Table 4:** Chromosome rearrangement of dog compared to mink and ferret.

dog chromosome	mink scaffold	ferret scaffold
1,12	nn66	mf37
14,16	nn4	mf7
2,5	nn3	mf13
5,6	nn58	mf93
3,13	nn45	mf102
10,15	nn21	mf10
2,11	nn22	mf14
14,18	nn35	mf4
18,21	nn152	mf52
19,32	nn145	mf22
19,36	nn40	mf61
36,37	nn182	mf96
14	nn4	mf7
16	nn31	mf60
7	nn2	mf34
8	nn50	mf47
15	nn100	mf6
17	nn139	mf113
18	nn46	mf94
26	nn187	mf9

### Gene annotation and orthologous analysis

**Figure 3:**
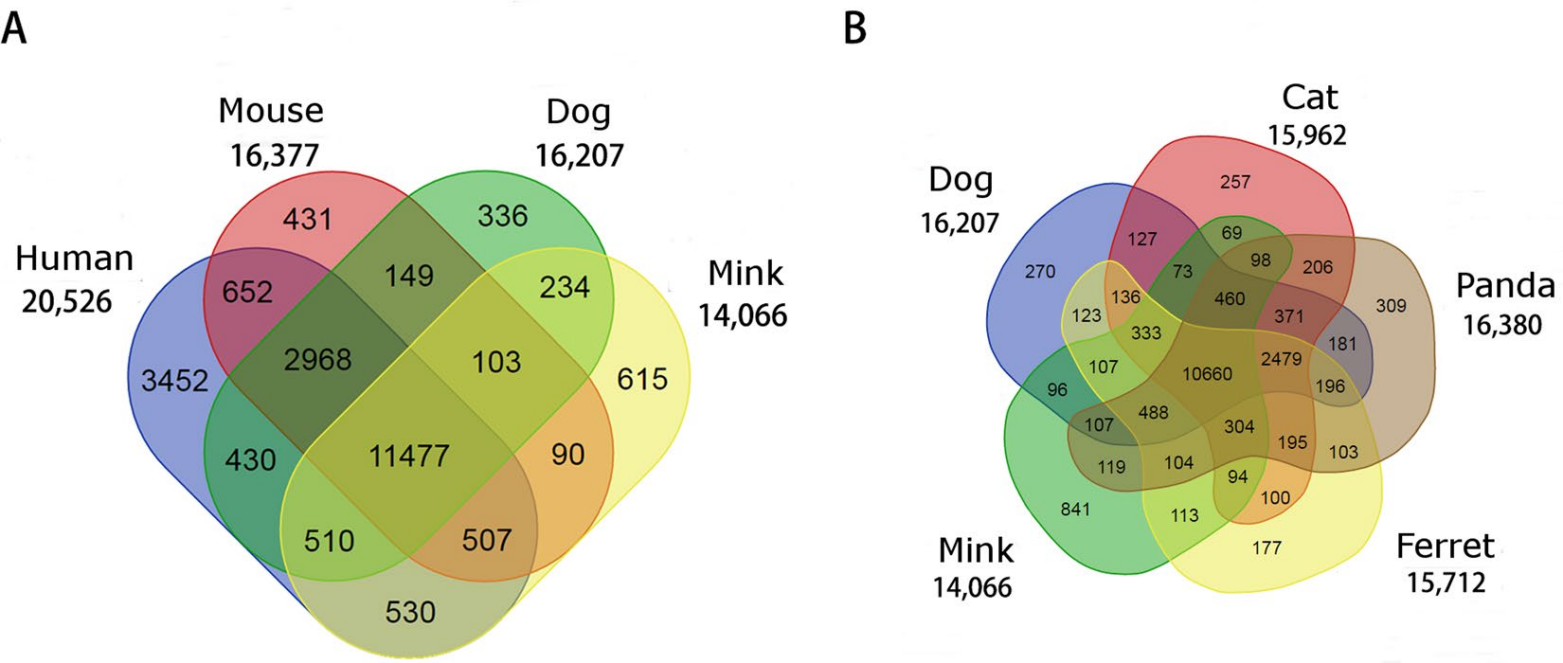
Unique and shared gene families between (**A**) the human, mouse, dog and mink genomes; (**B**) dog, cat, panda, ferret and mink genomes. Numbers in Venn diagram represent the number of gene family and number under each species represent total number of gene family in these species.

Combining *ab inito* gene prediction, protein alignment and transcriptome assembly^40^, we identified 21,053 protein-coding genes in the mink genome assembly. To create a detailed annotation of these genes, mink orthologous gene families were identified by scanning EggNOG^41^ mammalian database with HMMER3^42^. Moreover, two comparison analyses were performed by comparing mink orthologous gene families with three mammalian genomes namely human, mouse, dog, and with four Carnivora species namely dog, cat, panda and ferret. The mink proteome contained 14,066 orthologous gene families containing 17,052 genes. Among these, 11,477 gene families in mink were shared by all other three mammalian genomes compared here (Fig. 3A). Subsequently we checked the sequence similarity of mink genes identified here against genes from all species deposited in the OrthoMCL database^43^. Out of 15,608 genes which could be identified in the OrthoMCL data, 7,645 genes were most similar to dog genes compared with other species (Supplementary File 2). Gene family comparison among five Carnivora species (Fig. 3B) showed that our mink genome assembly has 841 orthologous gene families (990 genes) not shared with these four Carnivora genomes. The overlap of mink unique orthologous groups in Fig. 3A and Fig. 3B was 355. We further checked the 4,001 genes that were not mapped to any mammalian gene families. 1,119 of them matched other animals’ genes (EggNOG animal collection). These data suggested either mink retains a large amount of genes that may have been lost in other Carnivora genomes and mammalian genomes or we have some putative pseudogenes that show similarity to proteins from other kingdoms.

### Integrating the linkage map with the reference assembly and identification of fur quality genes

The linkage maps are useful tool for genetic research. We downloaded 103 microsatellite (SSR) marker primers or whole clone sequences^12^. These SSR markers were mapped to the mink genome assembly and 71 of them were able to anchor in the assembly. A total of 51 scaffolds harbored a single marker. Seven scaffolds had two markers. Among these, twelve markers were mapped relatively close to each other validating both the genetic and the physical distances. Two scaffolds had three markers, and all of them were located close to each other in same order as in the linkage map (Supplementary Table S7). These results demonstrated the agreement of the draft genome assembly with the linkage map. We mapped 21 fur color and fur quality genes (Supplementary Table S8) to the mink genome assembly, and compared their physical locations on the genome assembly with the physical locations of SSR markers within previously associated QTLs^13^ for fur properties. Four genes of the 21 candidates were located in QTL intervals (Table 5). According to Uniprot^44^, HLADRB1 (HLA-DR beta chain) is involved in membrane development and immune response. MITF (microphthalmia-associated transcription factor) may play a vital role in regulation of tyrosinase (TYR) and tyrosinase-related protein 1 (TYRP1) and also in the differentiation of various types of cell^45,46^. AGRP (agouti-related protein) may be involve in feeding behavior^47^. Finally PMEL (melanocyte protein) involved in the biogenesis of melanosomes^48^. Even though we found these genes close to fur quality QTLs, we could not conclude that variation in these genes was responsible for the QTL affecting fur quality. However, it demonstrated that the draft genome serves as a platform for multiple research approaches.

**Table 5:** The genes close to fur quality QTLs.

Chromosome	chromosomal position with 95% confidence intervals^13^	Gene	Traits
1	0.0–100.0 cM	HLADRB1	Guard hair thickness
6	23–26 cM	MITF	Guard hair thickness, guard hair length, wool density and quality
7	34–37 cM	AGRP	Surface quality and skin length
12	23 cM	PMEL	Surface

## Discussion

With the development of next generation sequencing technology, genome assembly becomes feasible for non-model organisms. Even though the assembly process is still computationally challenging for large genome, reference genomes for more and more species have been published^49^. Instead of following the conventional strategy, we assembled sequences from two individual minks, then some of the sequencing libraries were used to build a combined assembly and finally, simulated long insert reads from one individual were used to scaffolding combined assembly to build a consensus draft assembly. The reasons to adopt this strategy are the following: Firstly, ALLPATHS-LG^26^ has a well-developed algorithm to assemble the consensus sequence for polymorphic regions. Secondly, the sequencing of human^50^ and cattle^51^ reference genomes was also based on multiple individuals. Thirdly, the computation power to assemble combined dataset from both individuals exceeded the available 1TB server capacity. We also tried a different less memory-consuming assembler, however the result was smaller scaffold N50 and more scaffolds number. Finally, the available algorithms for merging assemblies were all not suitable for large genomes so we used the simulated MP data and additional scaffolding to achieve this.

American mink *(Neovison vison)* has an estimated genome size of 2.7 Gb^29^ and among the assemblies of large genome species, we achieved a competitive number of N50. We assembled 7,175 scaffolds with an N50 of 6.3 Mb. Among published genome assemblies, this number ranges from 10 kb (two-toed sloth) to 47.0 Mb (horse)^52^. Alignment of three sequencing libraries and two external BAC-end libraries (Supplementary Table S3) confirmed the validity of our assembly. Moreover, compared with the sequence alignment of ferret (Supplementary Table S4) we have achieved a comparable assembly. The adverse results of properly paired reads of two BES libraries and the ferret 40 kb library mostly came from the pairs aligned to different scaffolds. The assessment using BUSCO V2 with a mammalian orthologous gene set showed our assembly represented genes well with 95.80% complete genes, 2.2% of genes fragmented and 2.0% of genes missing (Supplementary Table S5). All of above results showed we have a high quality mink genome. The quality of the assembly largely influences the following analysis, as we can see from the BUSCO V2 result, the draft assembly has 2% more complete mammalian single copy-gene detected compared with the Pearl-mink-assembly (Supplementary Table S5). In addition, the genome alignment also depends on high quality assembly. The less fragment assembly not only largely reduces the complexity of genome alignment but also helps to detect the genome rearrangement between species. To further improve our assembly, long reads like PacBio^53^ can help to increase the contiguity of assembly^54^ and to reduce the ambiguous sequence in the assembly^55^. To solve further distance than the libraries we included in our assembly, we need Optical mapping data^56^ or chromatin interaction data^57^. With the help with long reads, we will have better genome annotation.

The mink genome showed less repeat sequence compared to the dog and cat genomes. However, all of these genomes have LINEs being the most abundant and SINEs being the second. The similarity of the genome repeats composition may be caused by the relatively recent separation of Carnivora species^20^. However, from the substitution level of these three species, we can see the difference in the dynamics of repeat sequence. As shown in Fig. 1, the dog and cat genomes have more repeat sequence elements and showed high identity to consensus sequence judged from the low substitution rate. In addition, the newly inserted elements are easier to detect in assembly. This suggests that dog and cat genomes have more recently inserted repeat sequences compared to mink. We know eukaryotic genomes have different amount of repeat sequence and also different genome structure because of the differential propagation and deletion of these elements^58^. Even within the closely related species like dog, cat and mink, the repeat sequence dynamics are different. By the genome alignment of mink and ferret to dog, we identified several specific rearrangements that must have happened since the last common ancestor of the three species and either the current dog population or the last common ancestor of mink and ferret. These findings will help us to better understand the evolution of Carnivora.

Genomic selection^59^ has revolutionized breeding of several livestock animals. This constitutes a paradigmatic shift from the time before reference genomes were available^60^. A reference genome assembly allows reliable identification of large numbers of markers and thereby facilitates application of genomic selection in practice. A reference genome assembly will also greatly improve breeding using genomics tools in the mink industry. With the reference, we can integrate available genetic research results within the genome and make them more sharable between different research and development groups working in mink breeding. For example, by combining the linkage map, the location of fur quality genes and fur quality QTLs^13^, we can search for genetic variants contributing to fur quality. The next step after the reference mink genome would be generating abundant markers covering the whole genome including SNPs which are the markers of choice today^61^. With this, the application of genomic selection and performing genome-wide association analysis will become feasible in mink.

## Methods

### Genomic data generation and genome assembly

The first mink sequenced was a male pearl American mink (*N. vison*) individual from the Aarhus University farm, Denmark. Genomic DNA was isolated and sequencing was performed by AROS (http://arosab.com). In order to use the ALLPATHS-LG pipeline^62^, we designed one overlapping pair-end library (165 bp, 100 PE) and two long insert size mate-pair library (3 kb and 5 kb, 100 PE). All data were generated by HiSeq. 2500 platform (Illumina Inc. San Diego, CA, USA). The total data were 163.2 Gb for pair-end sequence and 184.3 Gb for mate-pair sequence. The second animal sequenced was a brown mink female individual obtained from a private farm. For *de novo* assembly using ALLPATHS-LG sequencing libraries with different insert sizes were applied. Illumina libraries for the HiSeq. 2000 platform were generated following manufacturer’s protocol: two paired-end libraries with overlapping 150 bp reads and 600 bp inserts, respectively; three mate-pair libraries with 3 kb, 6 kb and 10 kb inserts. In addition, 8 kb, 14 kb and 32 kb insert libraries were acquired through Eurofins (www.eurofinsdna.com). Sequencing data of pearl mink is deposited on European Nucleotide Archive (ENA, http://www.ebi.ac.uk/ena) with accession number ERR1676595-ERR1676603 and sequencing data for brown mink is under project PRJEB16307. Due to the limitation of server memory, we performed three assemblies. The first was the Pearl-mink-assembly (PMA), which used all sequencing data from Pearl mink. The second one was the Brown-mink-assembly (BMA), which used part of Brown mink data (150 bp, 600 bp, 3 kb, 5 kb, 6 kb, 8 kb, part of 10 kb, 14 kb and 32 kb). The third assembly was hybrid-assembly (HA) with 165 bp PE library, 3 kb and 5 kb MP libraries from the Pearl mink and part of 600 bp PE library, 8 kb, part of 10 kb, 14 kb and 32 kb MP libraries from the Brown mink. To allow for genetic differences between the two individuals, we set PLOIDY = 2 and HAPLOIDIFY = True to ALLPATHS-LG, which helps to assemble the consensus sequence of polymorphic regions. Quality control and error correction were automated procedure within ALLPATHS-LG. Ultimately the hybrid-assembly and the Brown-mink-assembly were chose to construct the consensus draft assembly. In order to merge these two assemblies, we simulated 10k, 20k and 40k MP data from the second assembly with ART^28^ followed by a re-scaffolding of the HA by SSPACE^27^. The details of the libraries were listed in Supplementary Table 1S. The final assembly is deposited on ENA with accession ERZ337136.

### Assembly assessment

To check the correctness of the assembly and additional scaffolding procedure, we mapped genome sequencing data of the 150 bp, 600 bp and 3 kb libraries to the assembly using BWA^63^. In order to compare with the ferret assembly, ferret sequence libraries 180 bp (SRR085065), 3 kb (SRR085064), 6 kb –10 kb (SRR253162) and 40 kb (SRR253149) were downloaded from DNAnexus (https://www.dnanexus.com/) and aligned to the ferret genome with the same procedure. BWA men infers the reads orientation and the insert size during alignment and the properly paired reads were reported from SAMtools^64^ flagstat. Two external BES (BAC-end sequence) libraries^5,30^ were also used to check the correctness of the draft assembly. Firstly, the two BAC-ends of the same BAC clone were collected and renamed; BAC clones missing one of the end reads were removed. Then, the BAC-end sequences were aligned using BWA^63^. If two ends of the same BES aligned to different scaffolds or only one end was mapped, the BAC was marked as not properly paired. Also, BUSCO v2^31^ was used to check the completeness of genome assembly using vertebrate core genes. We ran BUSCO with the mammalian orthologous gene set (4,104 genes), calculated the complete, fragment, and lost genes in the assembly. We also used RNAmmer^65^ to detect the 45S ribosomal DNA cluster in our assembly.

### Repeat sequence annotation

RepeatExplorer^35^ and RepeatModeler^34^ were used to perform *de novo* prediction of novel repeat sequences in the mink genome before running RepeatMasker. RepeatExplorer’s result was analyzed by aligned contigs against the NR database^66^ using BLAST^67^. Clusters containing non-repeat sequences were removed from the result. Consensus satellite repeat sequences were extracted by Tandem Repeat Finder^68^. The RepeatModeler pipeline was used to obtain a consensus sequence for each repeat family. Finally, results from RepeatExplorer and RepeatModeler were combined with the dog repeat sequence database^36^ to construct a repeat database for mink. We used this repeat database with RepeatMasker^69^ to annotate repetitive sequences in the mink genome. The substitution level calculation and plots were done using calcDivergenceFromAlign.pl and createRepeatLandscape.pl scripts provided with RepeatMasker.

### Genome alignment

The soft-masked genomic sequence of mink and ferret (MusPutFur 1.0) were aligned to soft-masked dog (CanFam 3.1) genome downloaded^70^ from Ensembl using LASTZ^71^. Before alignment, we removed all the sequences named Unknown from dog genome. The pairwise genome alignment was chained according to their location in both genomes. The netting process chose for the reference species the best sub-chain in each region. A custom-made python script conducted the statistic of the block size. The genome ring figures were generated by Circos^72^.

### Gene annotation

The whole procedure of annotation consisted of *ab initio* gene prediction, homology-based prediction and RNA-seq. The information was merged together by the EVM^40^ weighted algorithm to build a consensus gene set. 1) AUGUSTUS^73^ with human parameter settings was used to perform the *ab initio* gene prediction. 2) Protein alignment was performed by Exonerate^74^ and Spaln^75^ using the Uniprot^44^ database. 3) An American mink transcriptome was available^14^. In order to improve the annotation, we re-analyzed this RNA-seq data set (PRJEB1260). Using Trinity^76^, we performed both *de novo* transcriptome assembly and also genome-guided transcriptome assembly. Both assemblies were then used in the annotation pipeline. PASApipeline^77^ was used to generate gene structures from the two transcriptome assemblies and build a comprehensive transcriptome database. To avoid false positives generated during transcriptome assembly, we did not use it directly as evidence. Instead, we used PASApipeline. PASApipeline will first align transcripts to the genome and perform a new assembly based on its alignment, so we will have a PASA_alignment and a PASA_assembly for evidences for annotation. Then all evidences were combined by EVM setting weights for AUGUSTUS to 1, for Exonerate to 4, for Spaln to 4, for PASA_alignment to 1 and for PASA_assembly to 10.

### Orthologous gene families

The amino acid sequences from the mink genome were extracted from annotation and scanned against EggNOG^41^ mammalian database using HMMER3^42^. The best hit to the database for each gene was used to identify the orthologous groups. Mink orthologous groups were compared with human (GRCh37), mouse (NCBIM37) and dog (BROADD2). Similarity of mink genes with genes in other genomes was investigated using the OrthoMCL^43^ online service. For each gene in the mink genome, the genome with the closest match was identified. Likewise all the orthologous groups containing dog (CanFam 3.1), cat (Felis_catus_6.2), panda (ailMel1) and ferret (MusPutFur 1.0) were extracted to perform the same comparison among Carnivora species. Finally, genes which could not be assigned to any mammalian gene family were scanned against the EggNOG^41^ animal database using HMMER3^42^.

### Integration of the linkage map with assembly and identification of fur quality genes

The most recent version of mink linkage map^12^ was obtained. The forward and reverse primer of each microsatellite (SSR) marker was extracted. For microsatellites where the primer information was not available, we mapped the whole clone sequence which was used to design primers to genome assembly. Totally, 103 markers out of 104 markers were kept for analysis. For primer mapping, both primers mapped and distances in the range 200 to 300 bp were considered correct; for clone sequence alignment, we required full alignment with few mismatches. Potential fur quality and color gene set^5^ was mapped to genome using Exonerate^74^. We located the previously reported QTL^13^ interval by SSR markers and compared the scaffold location of these markers with the locations of genes. The genes located in the interval were extracted and annotated by Uniprot^44^.

### Availability of data

The datasets generated and/or analyzed during the current study are available on European Nucleotide Archive (ENA, http://www.ebi.ac.uk/ena) with accession number ERR1676595-ERR1676603 for Pearl mink; project PRJEB16307 for brown mink and ERZ337136 for the draft assembly.

## Electronic supplementary material

Supplementary Information

Supplementary Dataset
