# Supplementary material for: The first draft reference genome of the American mink (Neovison vison)
Source: Sci Rep. 2017 Nov 6;7:14564. doi: 10.1038/s41598-017-15169-z (PMC5674041; doi:10.1038/s41598-017-15169-z)
Supplement: Supplementary file 1 — Supplementary Information [file 41598_2017_15169_MOESM1_ESM.doc]

**The first draft reference genome of the American mink (*Neovison vison*)**

Zexi Cai, Bent Petersen, Goutam Sahana, Lone B. Madsen, Knud Larsen, Bo Thomsen, Christian Bendixen, Mogens Sandø Lund, Bernt Guldbrandtsen, Frank Panitz

Supplementary Table S1. Reads number for each libraries

| Library names | Insert size | Pairs number | Reads length | Source |
| --- | --- | --- | --- | --- |
| 165 bp | 165 bp | 816,052,282 | 100 | Pearl mink |
| 150 bp | 150 bp | 683,004,865 | 100 | Brown mink |
| 600 bp | 600 bp | 193,640,166 | 100 | Brown mink |
| 3 kb | 3 kb | 695,510,557 | 100 | Pearl mink |
| 3 kb_2 | 3 kb | 631,017,642 | 100 | Brown mink |
| 5 kb | 5 kb | 225,932,436 | 100 | Pearl mink |
| 6 kb | 6 kb | 127,619,025 | 100 | Brown mink |
| 8 kb | 8 kb | 4,091,316 | 150 | Brown mink |
| 10s kb | 10 kb | 648,081,361 | 50 | Brown mink |
| 10l kb | 10 kb | 41,926,661 | 150 | Brown mink |
| 14 kb | 14 kb | 2,921,299 | 150 | Brown mink |
| 32 kb | 32 kb | 313,902 | 150 | Brown mink |
| S10 kb | 10 kb | 219,289,023 | 100 | Simulate |
| S20 kb | 20 kb | 222,035,183 | 100 | Simulate |
| S40 kb | 40 kb | 224,792,351 | 100 | Simulate |

Supplementary Table S2. Assembly statistic for assemblies

|  | Pearl-mink-assembly^a^ | Brown-mink- assembly^b^ | Hybrid-assembly^c^ | Draft assembly^d^ |
| --- | --- | --- | --- | --- |
| Estimated genome size | 2.7 Gb | 2.7 Gb | 2.7 Gb | 2.7 Gb |
| Total assembly length | 2.43 Gb | 2.61 Gb | 2.41 Gb | 2.45 Gb |
| Total sequence | 2.31 Gb | 2.33 Gb | 2.27 Gb | 2.27 Gb |
| Contig count | 177,579 | 170,660 | 157,853 | 157,853 |
| Contig N50 | 27.6 Kb | 32.7 Kb | 28.9 Kb | 28.9 Kb |
| Scaffold count | 22,578 | 24,921 | 11,169 | 7,175 |
| Scaffold N50 | 646,304 bp | 3.4 Mb | 2.6 Mb | 6.8 Mb |

^a^, using only the data from Pearl mink; ^b^, using most of data from Brown mink;^c^, using all data from Pearl mink , and part of 600 bp, 8 kb ,part of 10 kb, 14 kb and 32 kb libraries from Brown mink; ^d^, using simulated data from Brown-mink-assembly to re-scaffold Hybrid-assembly.

Supplementary Table S3. Sequence alignment

|  | 150 bp | 600 bp | 3 kb | Bac-end1 | Bac-end2 |
| --- | --- | --- | --- | --- | --- |
| Reads aligned Pearl-mink-assembly | 98.06% | 98.24% | 91.04% | 99.35% | 90.81% |
| Properly paired^a^ Pearl-mink-assembly | 95.95% | 89.66% | 70.38% | 76.27% | 40.58% |
| Singletons^b^ Pearl-mink-assembly | 0.35% | 0.95% | 7.50% | 0.36% | 8.46% |
| Mapped to different scaffolds^c^ Pearl-mink-assembly | 1.59% | 7.02 | 11.00% | 20.84% | 36.22% |
| Reads aligned Brown-mink-assembly | 98.61% | 98.74% | 91.37% | 99.41% | 90.84% |
| Properly paired^a^ Brown-mink-assembly | 96.86% | 91.03% | 69.94% | 80.11% | 54.02% |
| Singletons^b^ Brown-mink-assembly | 0.29% | 0.68% | 7.29% | 0.33% | 8.22% |
| Mapped to different scaffolds^c^ Brown-mink-assembly | 1.31% | 6.52% | 11.58% | 18.28% | 25.47% |
| Reads aligned Hybrid-assembly | 98.10% | 98.28% | 90.97% | 99.32% | 90.95% |
| Properly paired^a^ Hybrid-assembly | 95.72% | 89.55% | 69.49% | 78.27% | 50.54% |
| Singletons^b^ Hybrid-assembly | 0.36% | 0.88% | 7.50% | 0.42% | 8.28% |
| Mapped to different scaffolds^c^ Hybrid-assembly | 1.82% | 7.22% | 11.56% | 18.46% | 27.68% |
| Reads aligned draft assembly | 98.10% | 98.28% | 91.15% | 99.32% | 91.00% |
| Properly paired^a^ draft assembly | 95.72% | 89.55% | 69.91% | 79.55% | 55.58% |
| Singletons^b^ draft assembly | 0.36% | 0.88% | 7.33% | 0.42% | 8.22% |
| Mapped to different scaffolds^c^ draft assembly | 1.79% | 6.95% | 11.35% | 17.47% | 23.57% |
| Estimated size | 146bp | 578bp | 2.4kb | 25kb | 164kb |
| Library size | 150bp | 600bp | 3kb | 20-50kb | 170 kb |

Note, ^a^Properly paired for 150 bp, 600 bp and 3 kb libraries means the properly paired reads flagged by BWA^1^ and reported by flagstat of Samtools^2^, for Bac-end1 and Bac-end2 means both end aligned to the same scaffolds. ^b^Singletons means only one end of reads can be mapped divided by total reads number plus supplementary (reads be split and both mapped); ^c^Mapped to different scaffolds means two ends of reads mapped to different scaffolds divided by total reads number plus supplementary (reads were split and both mapped).

Supplementary Table S4. Reads alignment of ferret

|  | 180 bp | 3 kb | 6 kb -10 kb | 40 kb |
| --- | --- | --- | --- | --- |
| Reads aligned | 88.93% | 93.15% | 95.90% | 86.42% |
| Properly paired^a^ | 79.20% | 80.64% | 82.06% | 63.77% |
| Singletons^b^ | 5.85% | 1.98% | 1.59% | 6.07% |
| Mapped to different scaffolds^c^ | 2.25% | 7.99% | 8.54% | 16.89% |

Note, ^a^Properly paired for 180 bp, 3kp and 6-10 kb libraries means the properly paired reads flagged by BWA^1^ and reported by flagstat of Samtools^2^, for 40 kb library means both end aligned to the same scaffolds. ^b^Singletons means only one end of reads can be mapped divided by total reads number plus supplementary (reads be split and both mapped); ^c^Mapped to different scaffolds means two ends of reads mapped to different scaffolds divided by total reads number plus supplementary (reads were split and both mapped).

Supplementary Table S5. BUSCO gene completeness assessment

|  | Pearl-mink-assembly | Brown-mink-assembly | draft assembly | ferret |
| --- | --- | --- | --- | --- |
| Complete | 93.80% | 94.9% | 95.80% | 95.20% |
| Fragment | 3.8% | 2.7% | 2.2% | 2.5% |
| Missing | 2.4% | 2.4% | 2.0% | 2.3% |


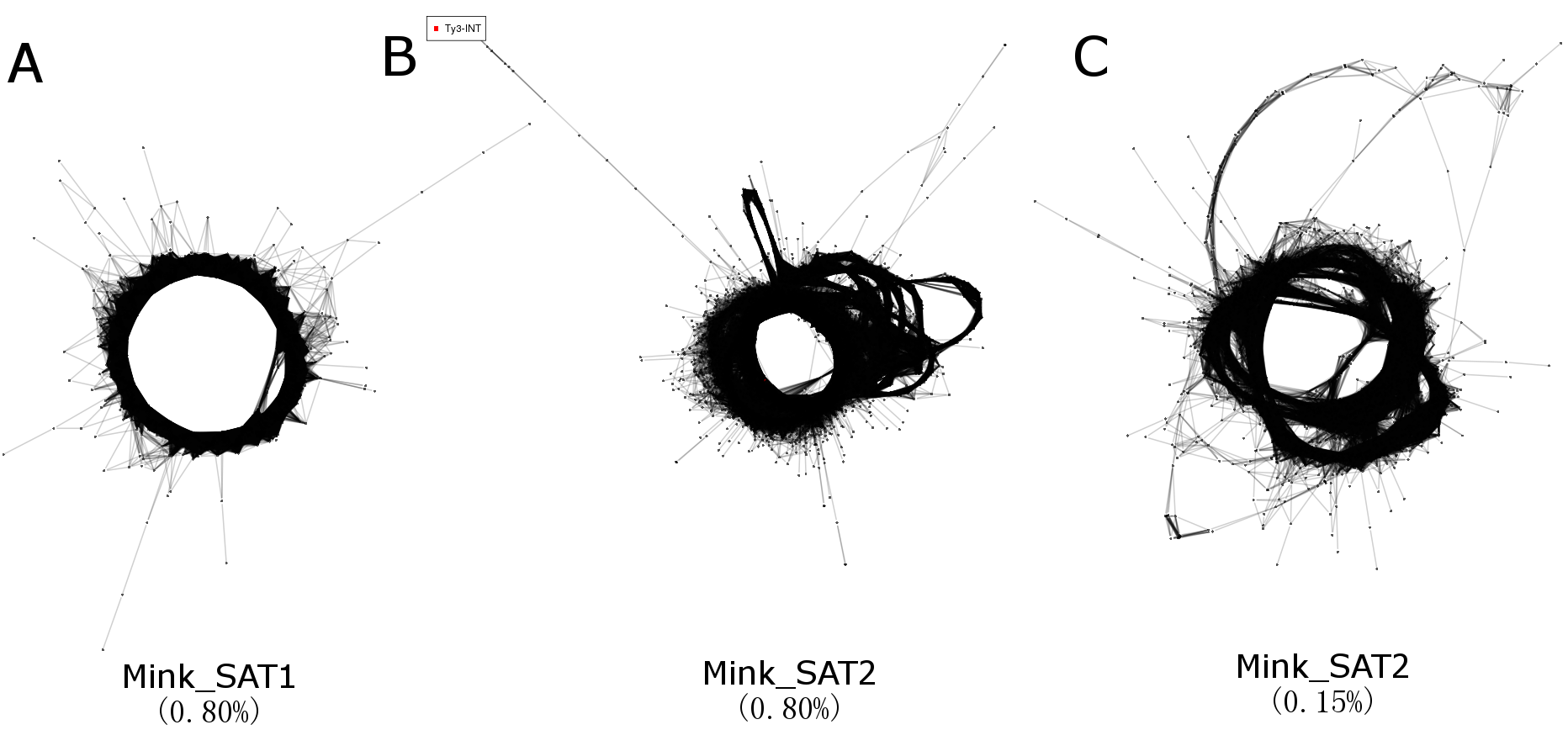


Supplementary Figure S1. The reads cluster of new found satellite repeats, the ring layout suggested satellite repeats. RepeatExplorer^3^ performs all-to-all similarity comparisons of sequence reads followed by their graph-based clustering to identify groups of reads derived from repetitive elements. (A) The layout and genome proportion of mink_sat1, it is similar to 1080bp Bam HI repeat DNA; (B) The layout and genome proportion of mink_sat2; (C) The layout and genome proportion of mink_sat3.

Supplementary Table S6. RepeatModeler^4^ build mink repeat database

| **Family** | **Number** |
| --- | --- |
| LINE | 94 |
| LTR | 93 |
| Transposon | 50 |
| SINE | 19 |
| Unknown | 19 |
| Satellite | 1 |
| Buffer | 3 |
| Simple_repeat | 2 |
| Total | 285 |


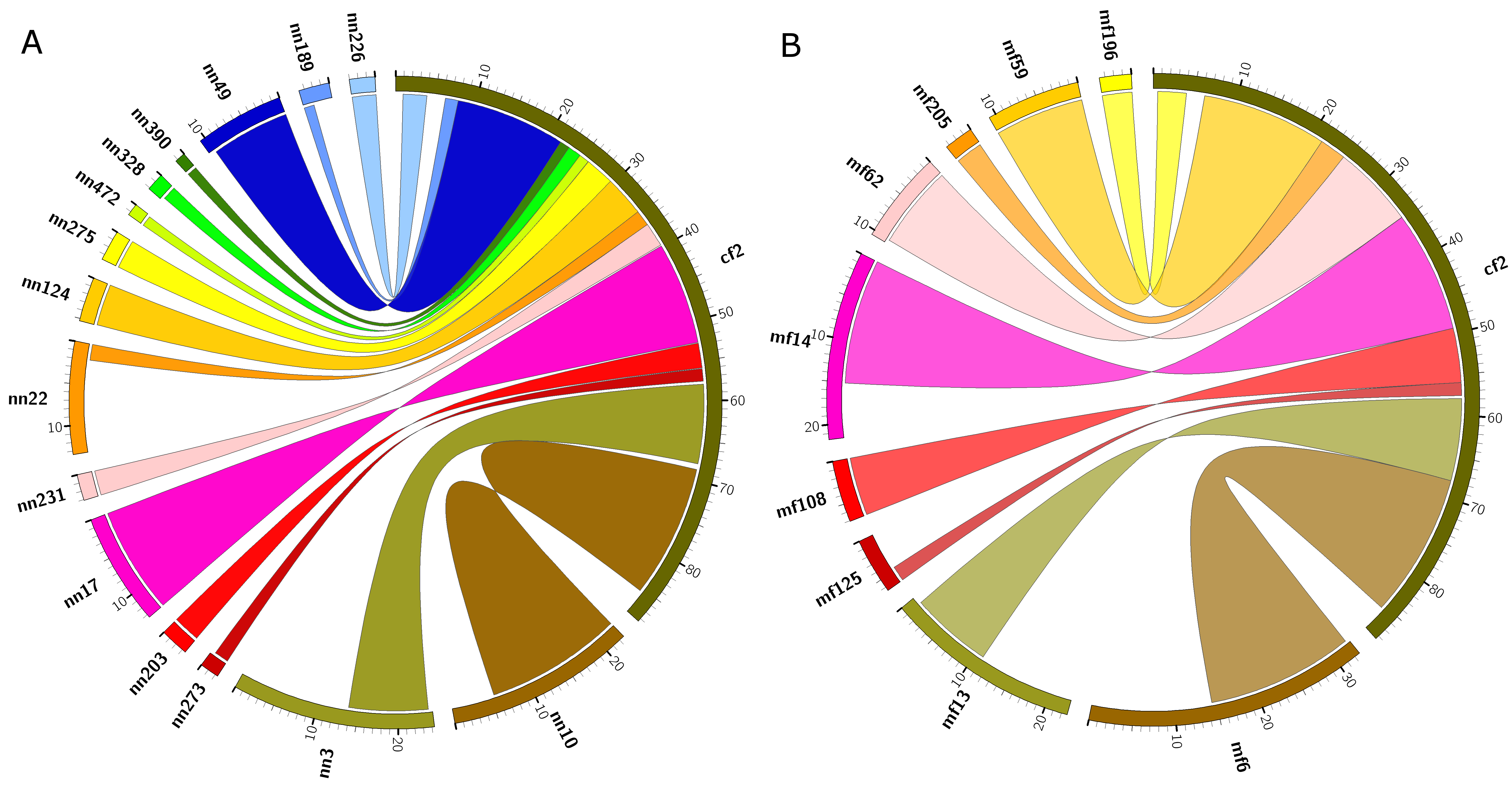


Supplementary Figure S2. Genome alignment of mink (A) and ferret (B) to dog chromosome 2 (cf2). The mink scaffold start with ’nn’ and ferret scaffold start with ’mf’. Position 14 Mb to 24 Mb of mink scaffold 3 (nn3) and position 1 Mb to 11 Mb of ferret scaffold 13 (mf13) can be aligned to position 58 Mb to 68 Mb of dog chromosome 2 (cf2). Position 0 Mb to 2 Mb of nn22 can be aligned to position 35 Mb to 37 Mb of cf2, and position 0 Mb to 15 Mb of mf14 can be aligned to position 35 Mb to 50 Mb of cf2. Position 0 Mb to 2 Mb of nn22 can be aligned to position 35 Mb to 37 Mb of cf2 and position 0 Mb to 15 Mb of mf14 can be aligned to position 45 Mb to 50 Mb of cf2.


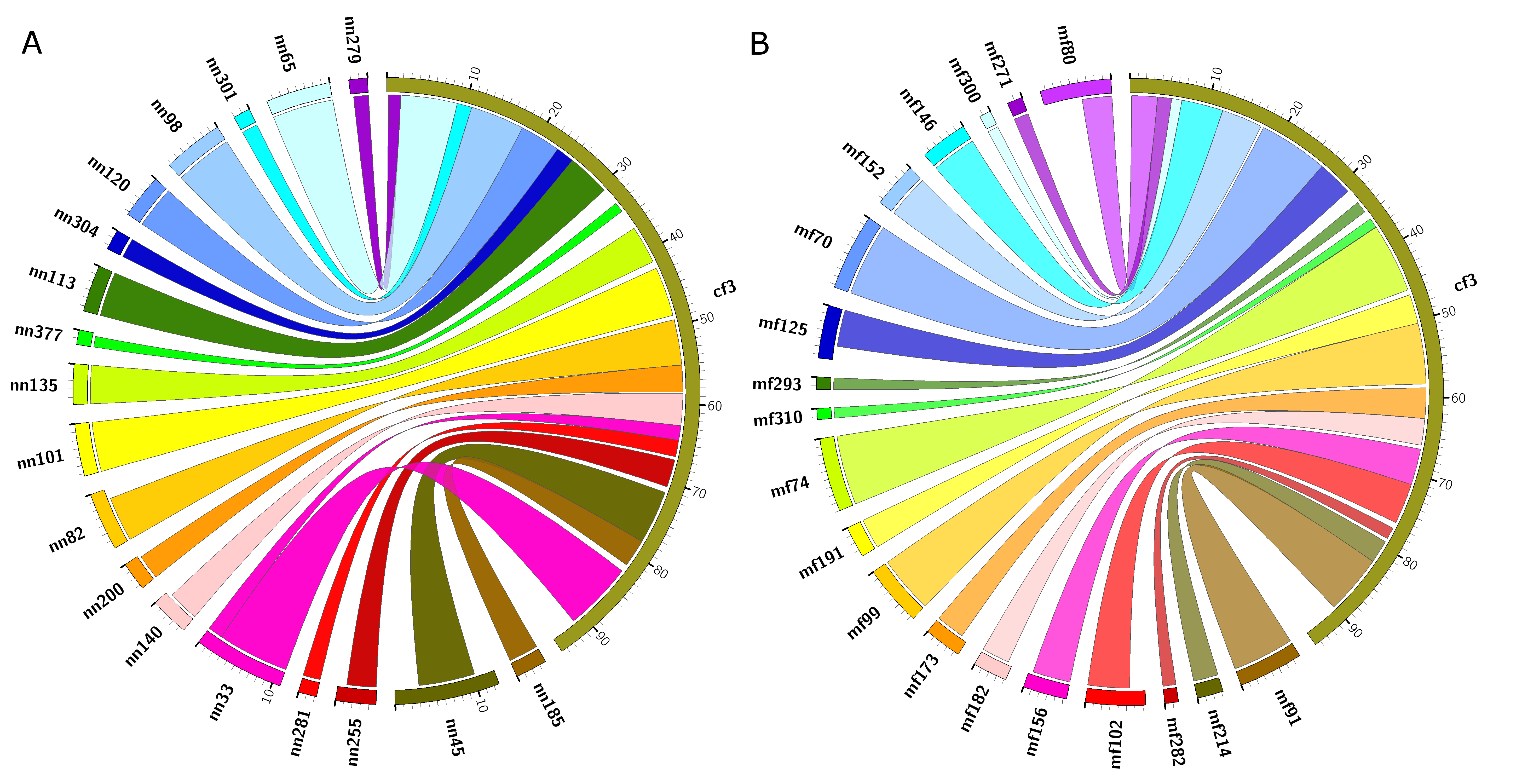


Supplementary Figure S3. Genome alignment of mink (A) and ferret (B) to dog chromosome 3 (cf3). The mink scaffold start with ’nn’ and ferret scaffold start with ’mf’. Position 3 Mb to 10 Mb of mink scaffold 45 (nn45) can be aligned to position 72 Mb to 79 Mb of dog chromosome 3 (cf3) and position 0 Mb to 5 Mb of ferret scaffold 102 (mf102) can be aligned to position 72 Mb to 77 Mb of cf3.


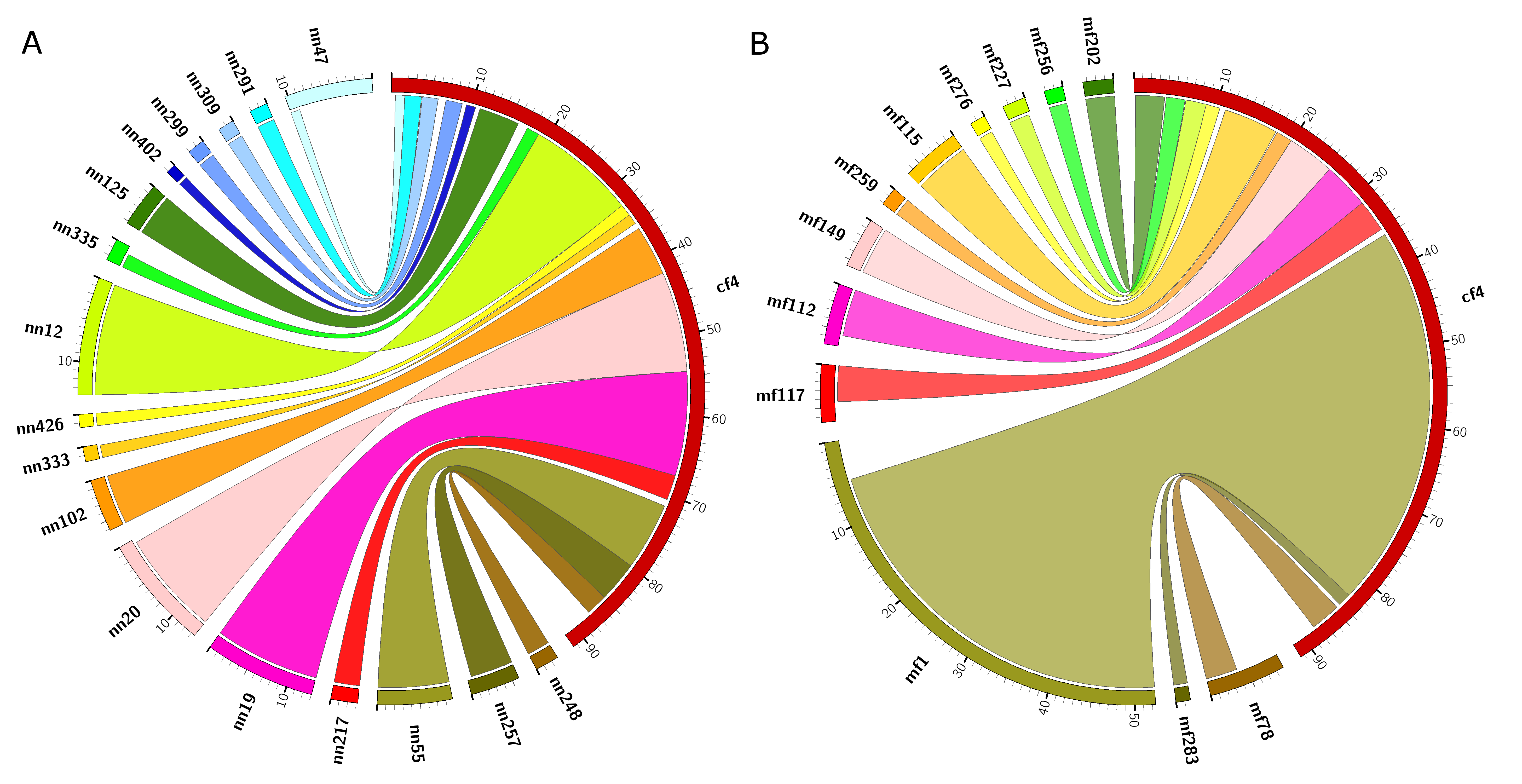


Supplementary Figure S4. Genome alignment of mink (A) and ferret (B) to dog chromosome 4 (cf4). The mink scaffold start with ’nn’ and ferret scaffold start with ’mf’.


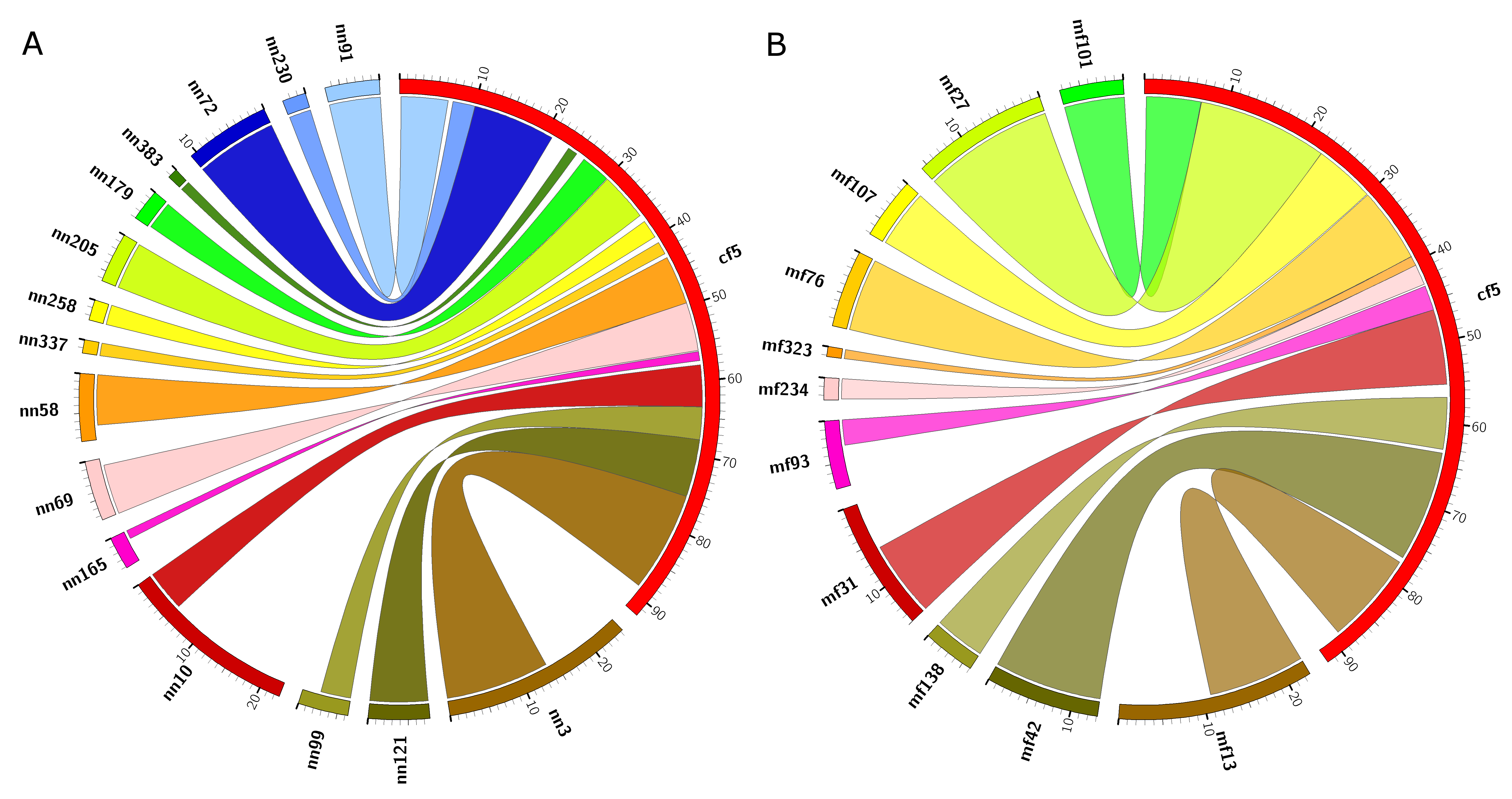


Supplementary Figure S5. Genome alignment of mink (A) and ferret (B) to dog chromosome 5 (cf5). The mink scaffold start with ’nn’ and ferret scaffold start with ’mf’. Position 0 Mb to 13 Mb of mink scaffold 3 (nn3) can be aligned to position 76 Mb to 89 Mb of dog chromosome 5 (cf5) and position 11 Mb to 22 Mb of ferret scaffold 13 (mf13) can be aligned to position 78 Mb to 89 Mb of cf5. Position 0 Mb to 7 Mb of nn58 can be aligned to position 42 Mb to 49 Mb of cf5 and 0 Mb to 3 Mb of mf93 can be aligned to position 43 Mb to 46 Mb of cf5.


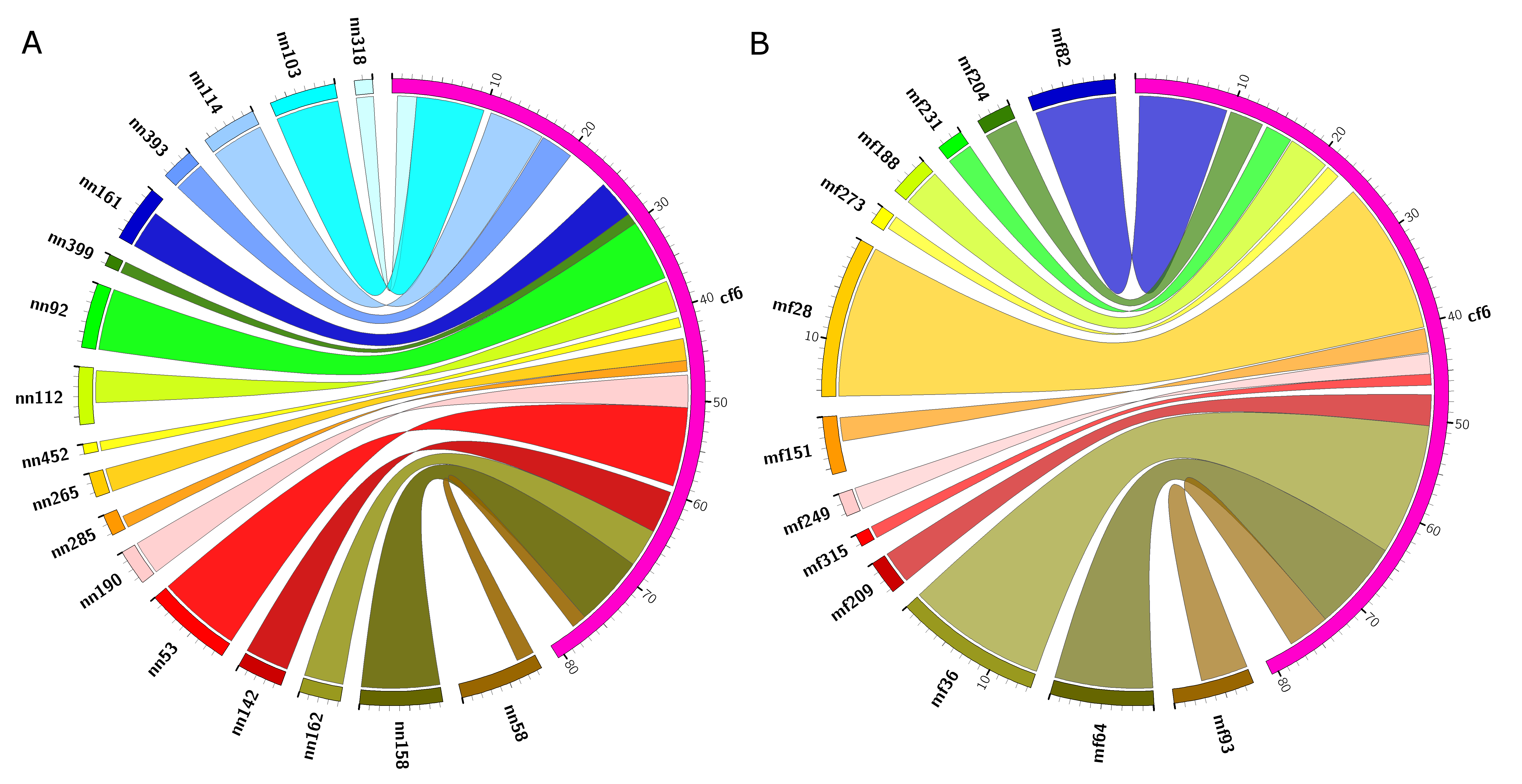


Supplementary Figure S6. Genome alignment of mink (A) and ferret (B) to dog chromosome 6 (cf6). The mink scaffold start with ’nn’ and ferret scaffold start with ’mf’. Position 6 Mb to 8 Mb of mink scaffold 58 (nn58) can be aligned to position 76 Mb to 78 Mb of dog chromosome 6 (cf6) and 3 Mb to 8 Mb of ferret scaffold 93 (mf93) can be aligned to position 73 Mb to 78 Mb of cf6.


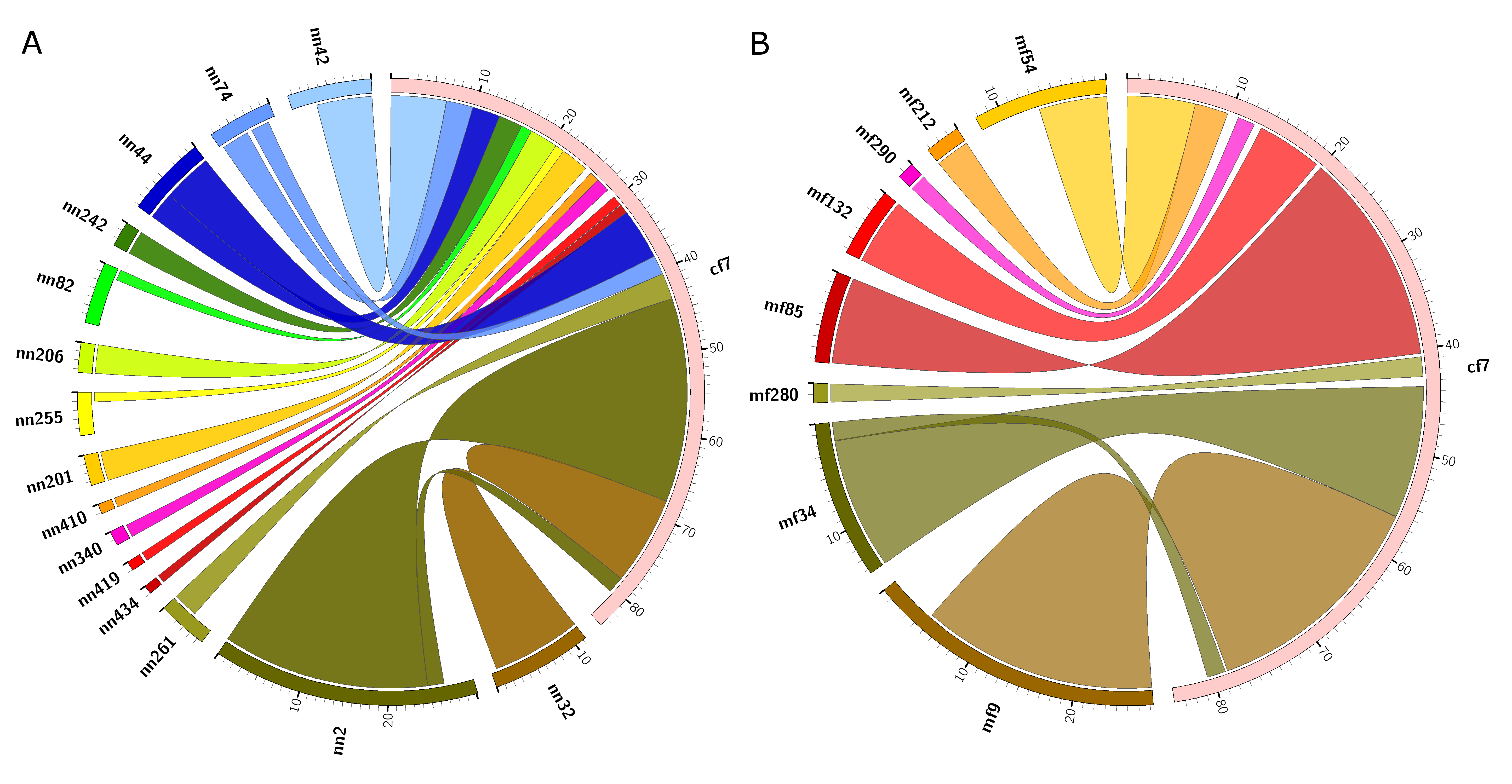


Supplementary Figure S7. Genome alignment of mink (A) and ferret (B) to dog chromosome 7 (cf7). The mink scaffold start with ’nn’ and ferret scaffold start with ’mf’. Position 24 Mb to 26 Mb of mink scaffold 2 (nn2) and position 0 Mb to 2 Mb of ferret scaffold 34 (mf34) can be aligned to position 79 Mb to 81 Mb of dog chromosome 7 (cf7) whereas position 0 Mb to 24 Mb of nn2 can be aligned to the reversed sequence of position 43 Mb to 68 Mb of cf7, and position 2 Mb to 14 Mb of mf34 can be aligned to the position 44 Mb to 56 Mb of cf7.


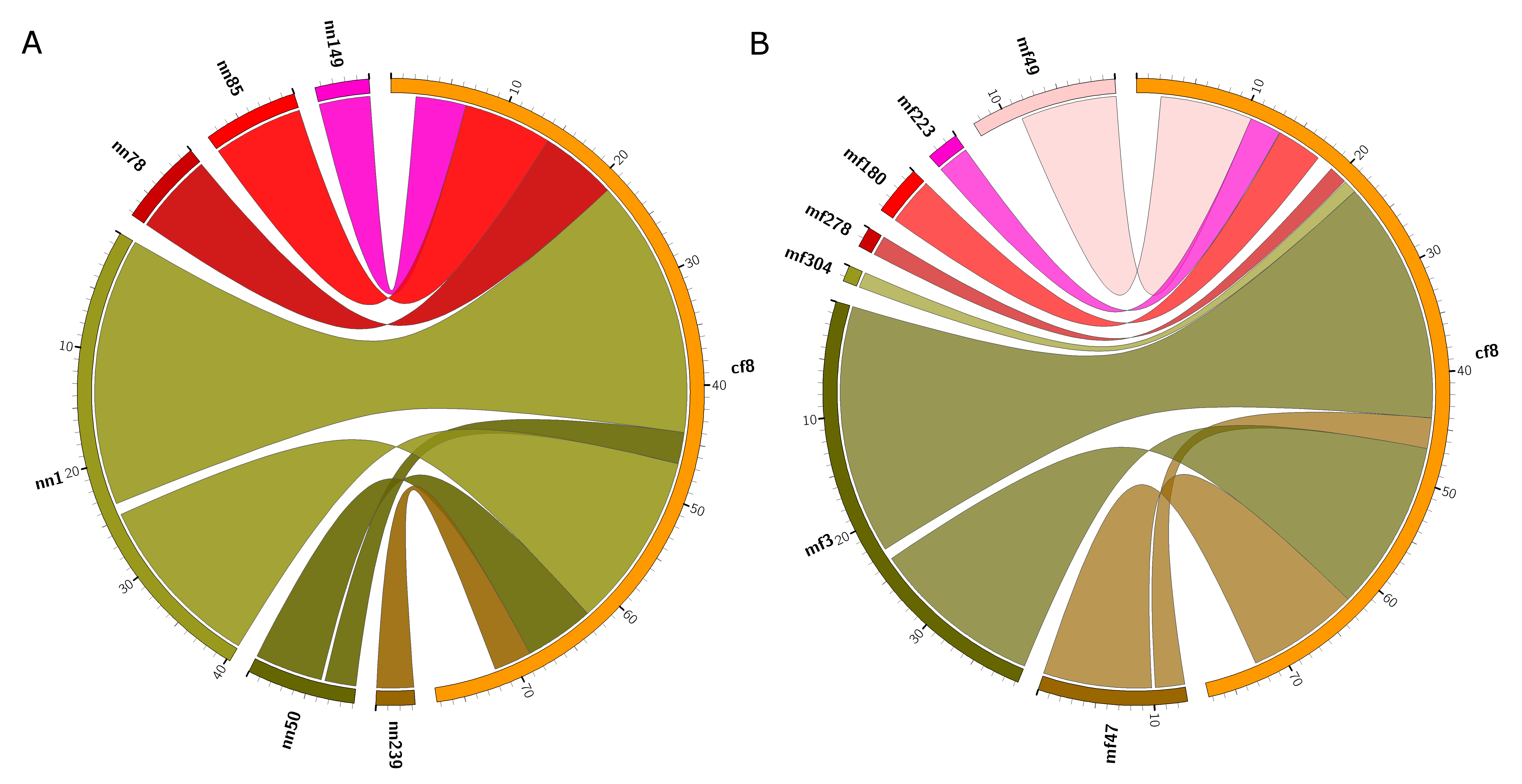


Supplementary Figure S8. Genome alignment of mink (A) and ferret (B) to dog chromosome 8 (cf8). The mink scaffold start with ’nn’ and ferret scaffold start with ’mf’. Position 6 Mb to 9 Mb of mink scaffold 50 (nn50) and position 10 Mb to 13 Mb of ferret scaffold 47(mf47) can be aligned to position 44 Mb to 47 Mb of dog chromosome 8 (cf8) whereas position 0 Mb to 6 Mb of nn50 can be aligned to the reversed sequence of position 62 Mb to 68 Mb of cf8, and position 0 Mb to 10 Mb of mf47 can be aligned to the reversed sequence of position 62 Mb to 72 Mb of cf8.


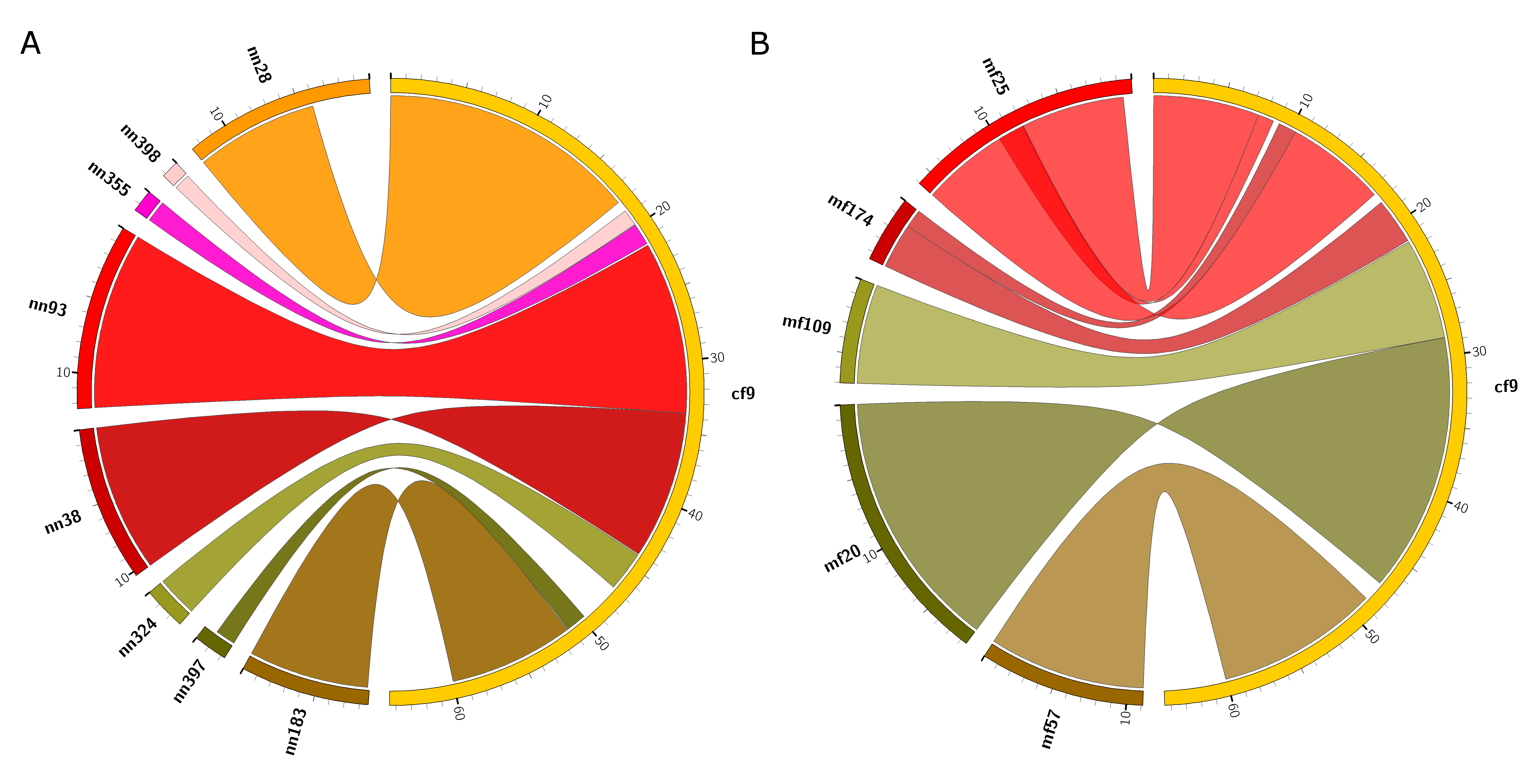


Supplementary Figure S9. Genome alignment of mink (A) and ferret (B) to dog chromosome 9 (cf9). The mink scaffold start with ’nn’ and ferret scaffold start with ’mf’.


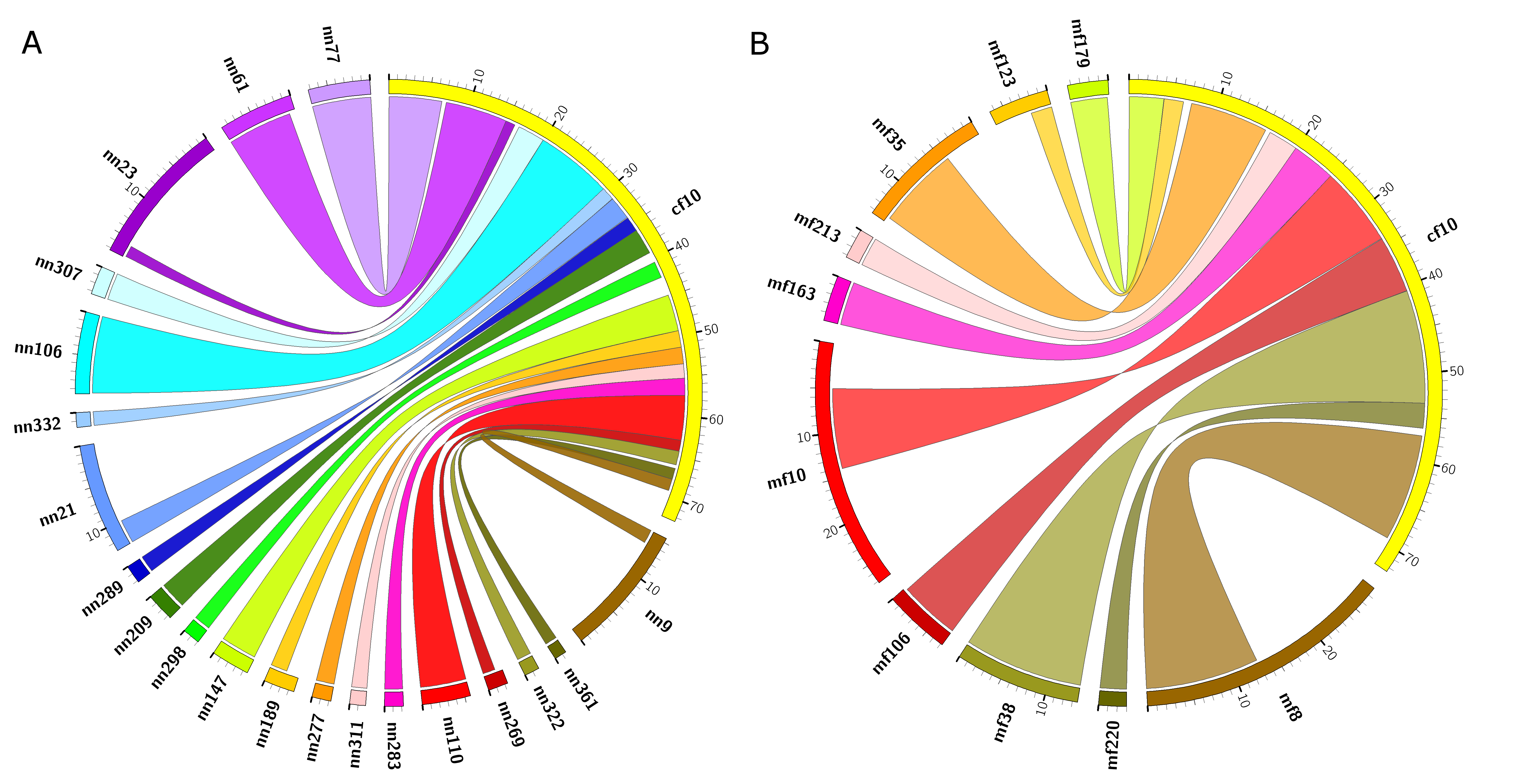


Supplementary Figure S10. Genome alignment of mink (A) and ferret (B) to dog chromosome 10 (cf10). The mink scaffold start with ’nn’ and ferret scaffold start with ’mf’. Position 10 Mb to 13 Mb of mink scaffold 21 (nn21) can be aligned to position 31 Mb to 34 Mb of dog chromosome 10 (cf10) and position 5 Mb to 14 Mb of ferret scaffold 10 (mf10) can be aligned to position 26 Mb to 35 Mb of cf10.


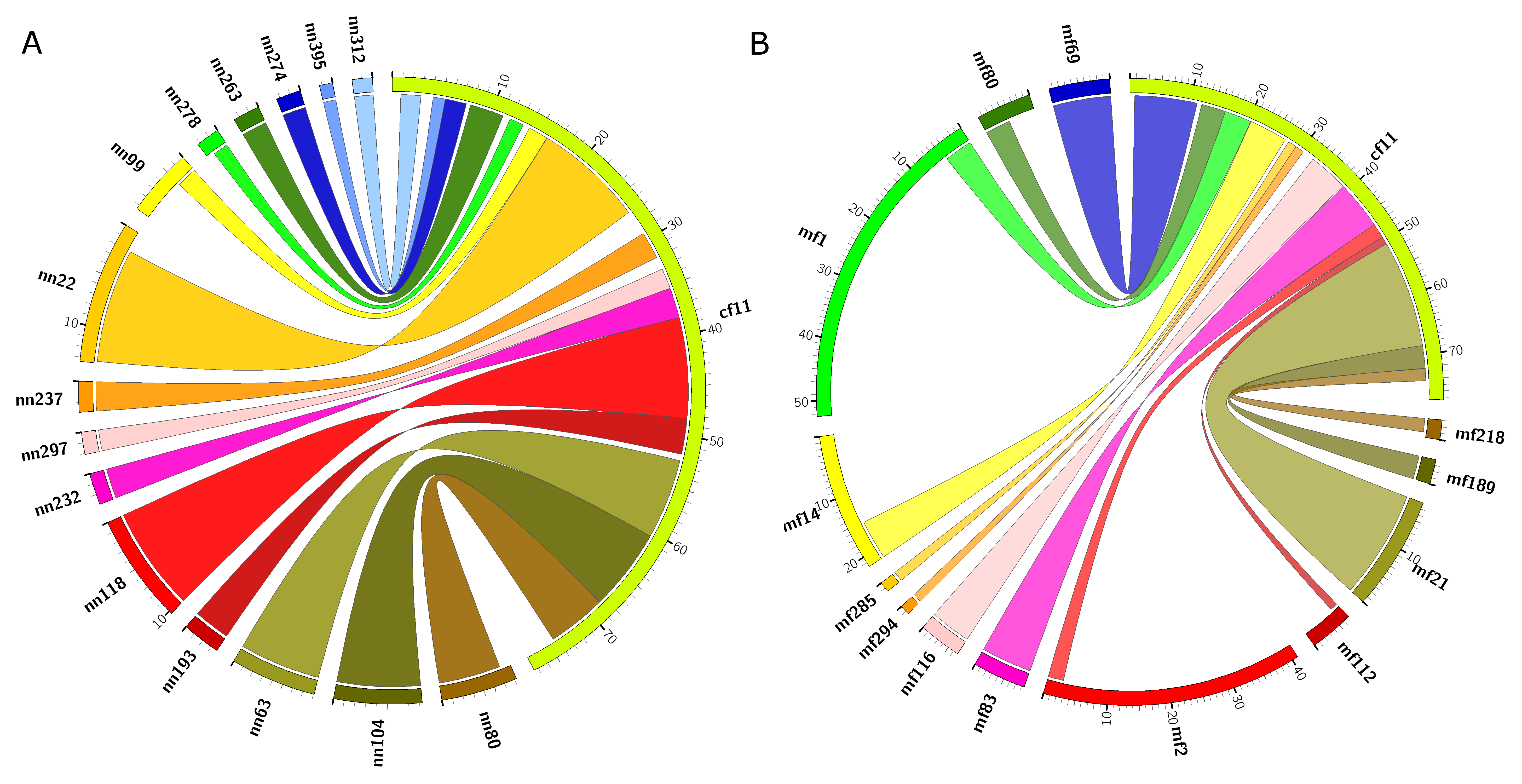


Supplementary Figure S11. Genome alignment of mink (A) and ferret (B) to dog chromosome 11 (cf11). The mink scaffold start with ’nn’ and ferret scaffold start with ’mf’. Position 2 Mb to 13 Mb of mink scaffold 22 (nn22) can be aligned to position 15 Mb to 26 Mb of dog chromosome 11 (cf11), and position 15 Mb to 21 Mb of ferret scaffold 14 (mf14) can be aligned to position 20 Mb to 26 Mb of cf11. Position 2 Mb to 13 Mb of nn22 can be aligned to position 16 Mb to 26 Mb of cf11 and position 15 Mb to 21 Mb of mf14 can be aligned to position 20 Mb to 26 Mb of cf11.


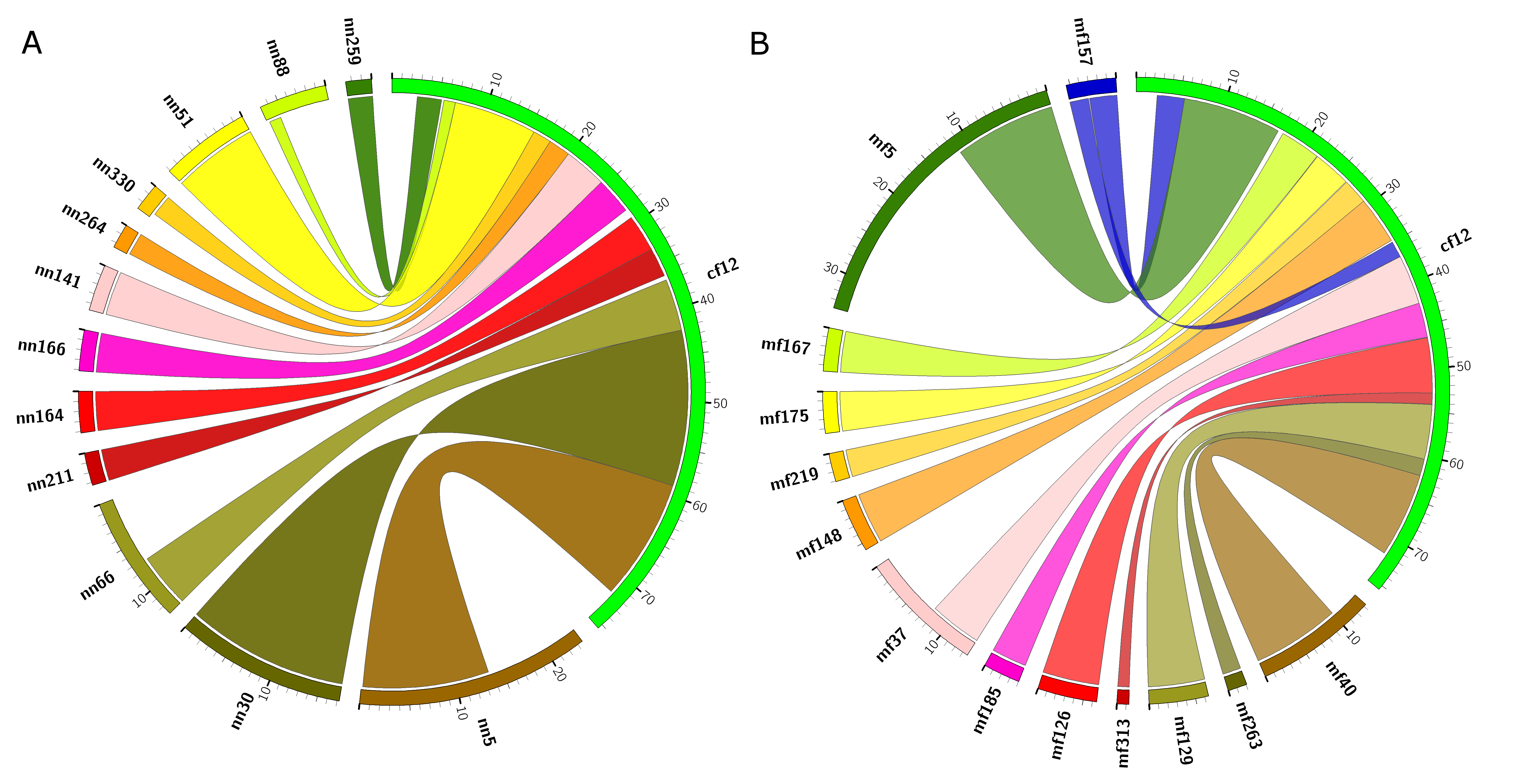


Supplementary Figure S12. Genome alignment of mink (A) and ferret (B) to dog chromosome 12 (cf12). The mink scaffold start with ’nn’ and ferret scaffold start with ’mf’. Position 7 Mb to 13 Mb of mink scaffold 66 (nn66) and ferret scaffold 37 (mf37) can be aligned to position 37 Mb to 42 Mb of dog chromosome 12 (cf12).


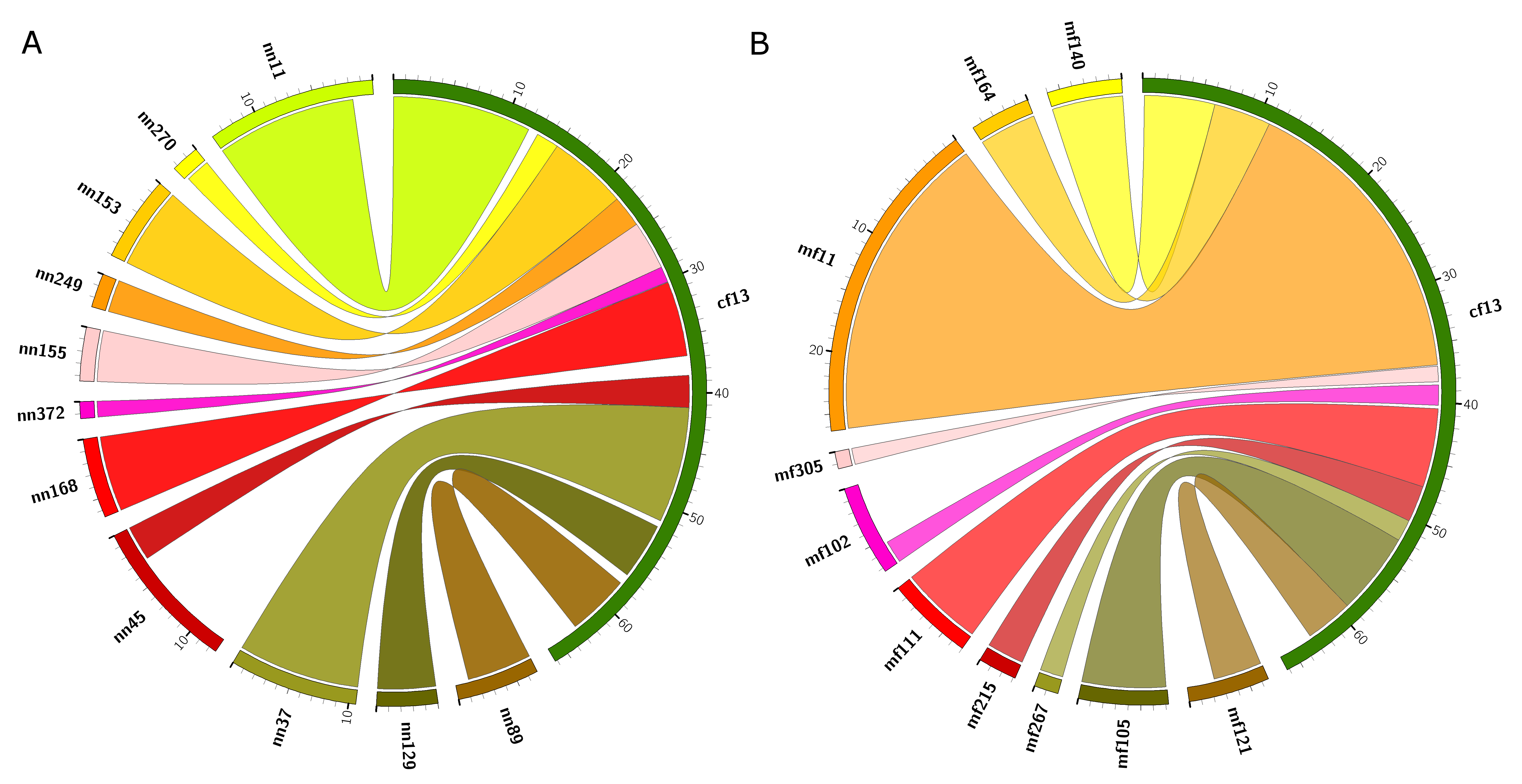


Supplementary Figure S13. Genome alignment of mink (A) and ferret (B) to dog chromosome 13 (cf13). The mink scaffold start with ’nn’ and ferret scaffold start with ’mf’. Position 0 Mb to 3 Mb of mink scaffold 45 (nn45) can be aligned to position 39 Mb to 41 Mb of dog chromosome 13 (cf13) and position 5 Mb to 7 Mb of ferret scaffold 102 (mf102) can be aligned to position 39 Mb to 40 Mb of cf13.


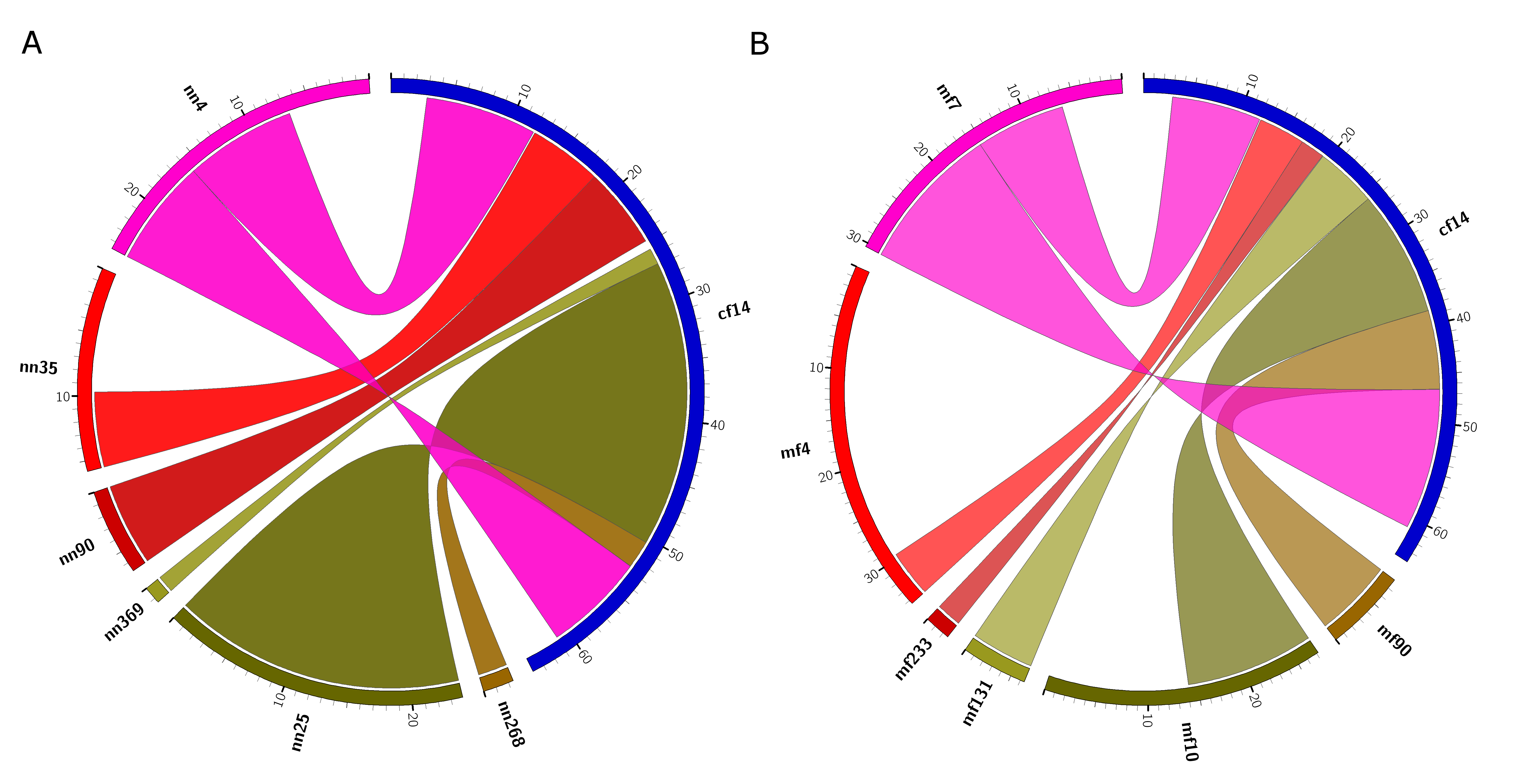


Supplementary Figure S14. Genome alignment of mink (A) and ferret (B) to dog chromosome 14 (cf14). The mink scaffold start with ’nn’ and ferret scaffold start with ’mf’. Position 7 Mb to 16 Mb of mink scaffold 4 (nn4) and ferret scaffold 7 (mf7) can be aligned to position 3 Mb to 12 Mb of dog chromosome 14 (cf14) whereas position 16 Mb to 25 Mb of nn4 can be aligned to the reversed sequence of position 52.5 Mb to 61 Mb of cf14, and position 16 Mb to 30 Mb of mf7 (mf7 is longer than nn4) can be aligned to the reversed sequence of position 47 Mb to 61 Mb of cf14. Position 10 Mb to 16 Mb of nn35 can be aligned to position 12 Mb to 18 Mb of cf14 and position 30 Mb to 34 Mb of mf4 can be aligned to position 12 Mb to 17 Mb of cf14.


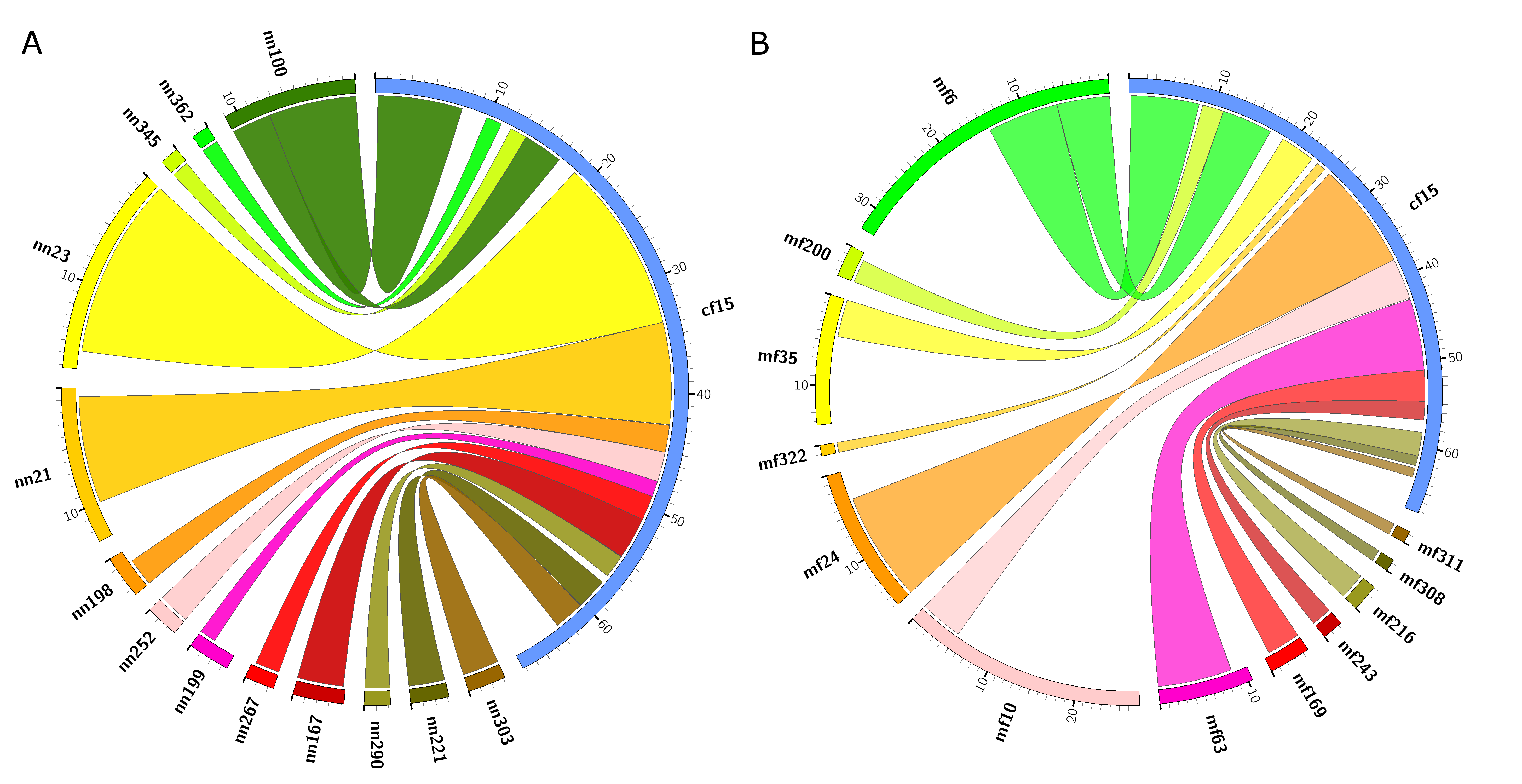


Supplementary Figure S15. Genome alignment of mink (A) and ferret (B) to dog chromosome 15 (cf15). The mink scaffold start with ’nn’ and ferret scaffold start with ’mf’. Position 1 Mb to 10 Mb of mink scaffold 21 (nn21) can be aligned to position 34 Mb to 43 Mb of dog chromosome 15 (cf15) and position 0 Mb to 6 Mb of ferret scaffold 10 (mf10) can be aligned to position 25 Mb to 37 Mb of cf15. Position 7 Mb to 11 Mb of nn100 and position 0 Mb to 6 Mb of mf6 can be aligned to position 13 Mb to 17 Mb of cf16 and position 11 Mb to 17 Mb of cf15 whereas position 0 Mb to 13 Mb of nn100 aligned to the reversed sequence of position 0 Mb to 8 Mb of cf15, and position 6 Mb to 14 Mb of mf6 aligned to position 0 Mb to 8 Mb of cf15.


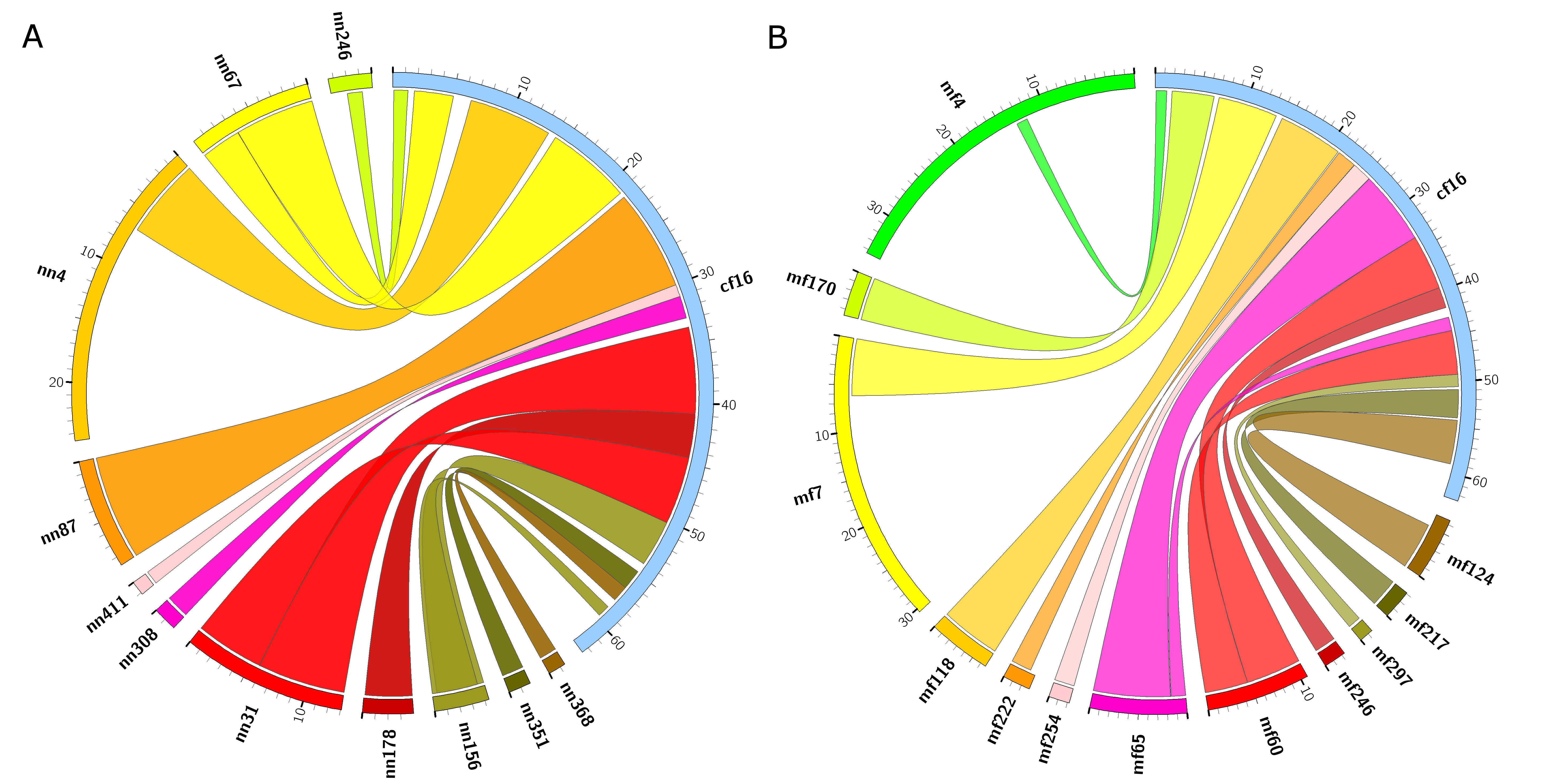


Supplementary Figure S16. Genome alignment of mink (A) and ferret (B) to dog chromosome 16 (cf16). The mink scaffold start with ’nn’ and ferret scaffold start with ’mf’. Position 0 Mb to 7 Mb of mink scaffold 4 (nn4) and ferret scaffold 7 (mf7) can be aligned to position 7 Mb to 14 Mb of dog chromosome 16 (cf16). Position 5 Mb to 13 Mb of nn31 and position 5 Mb to 11 Mb of mf60 can be aligned to position 33 Mb to 41 Mb of cf16 and position 33 Mb to 40 Mb of cf16 whereas position 0 Mb to 5 Mb of nn31 can be aligned to the reversed sequence of position 44 Mb to 50 Mb of cf16, and position 0 Mb to 5 Mb of mf7 can be aligned to the reversed sequence of position 44 Mb to 49 Mb of cf16.


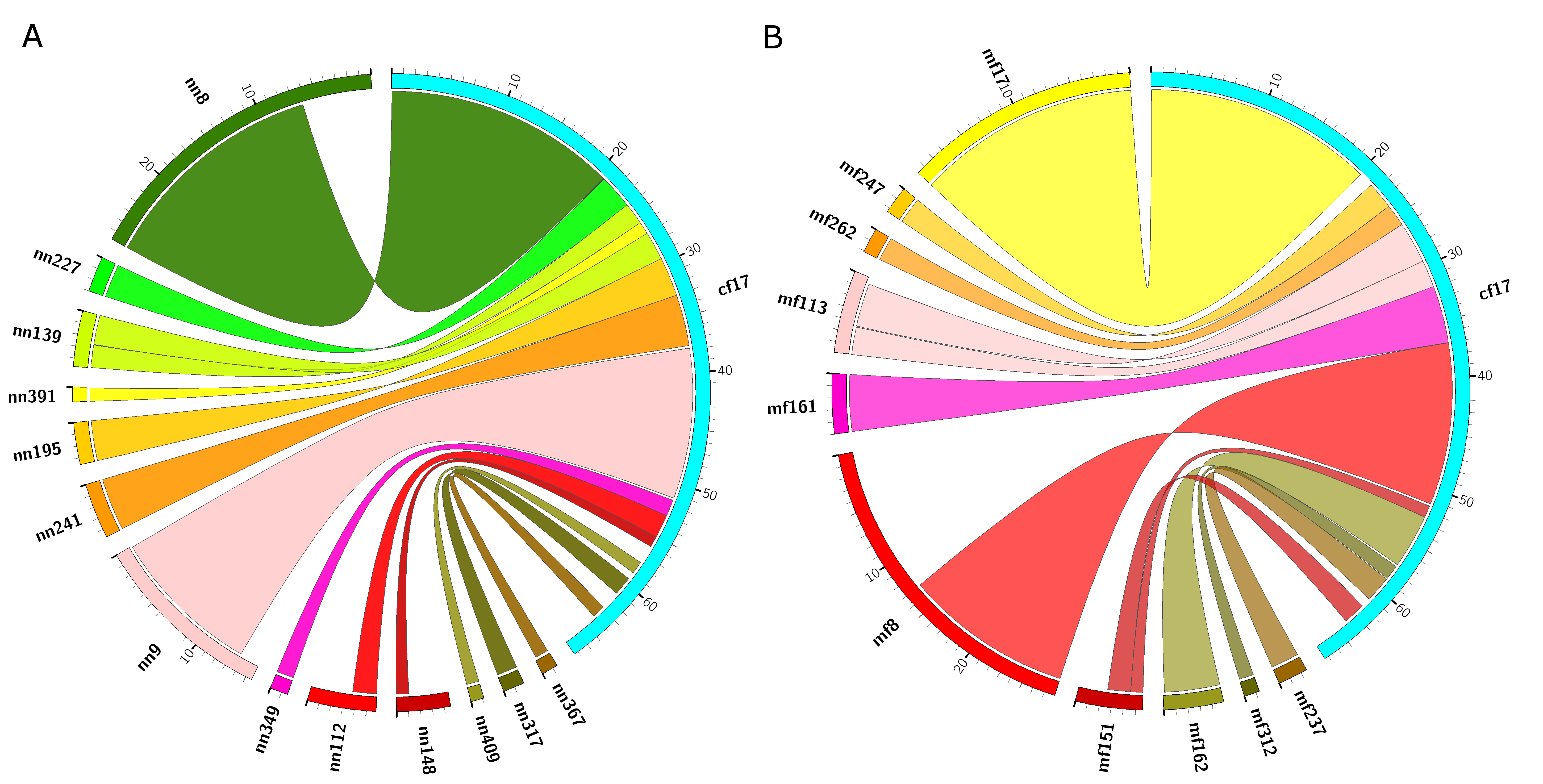


Supplementary Figure S17. Genome alignment of mink (A) and ferret (B) to dog chromosome 17 (cf17). The mink scaffold start with ’nn’ and ferret scaffold start with ’mf’. Position 3 Mb to 5 Mb of mink scaffold 139 (nn139) and position 4 Mb to 7 Mb of ferret scaffold 113 (mf113) can be aligned to the reversed sequence of position 24 Mb to 26 Mb of dog chromosome 17 (cf17) and position 29 Mb to 32 Mb of cf17 whereas position 0 Mb to 3 Mb of nn139 can be aligned to the position 27 Mb to 30 Mb of cf17, and position 1 Mb to 4 Mb of mf113 can be aligned to the reversed sequence of position 26 Mb to 30 Mb of cf17.


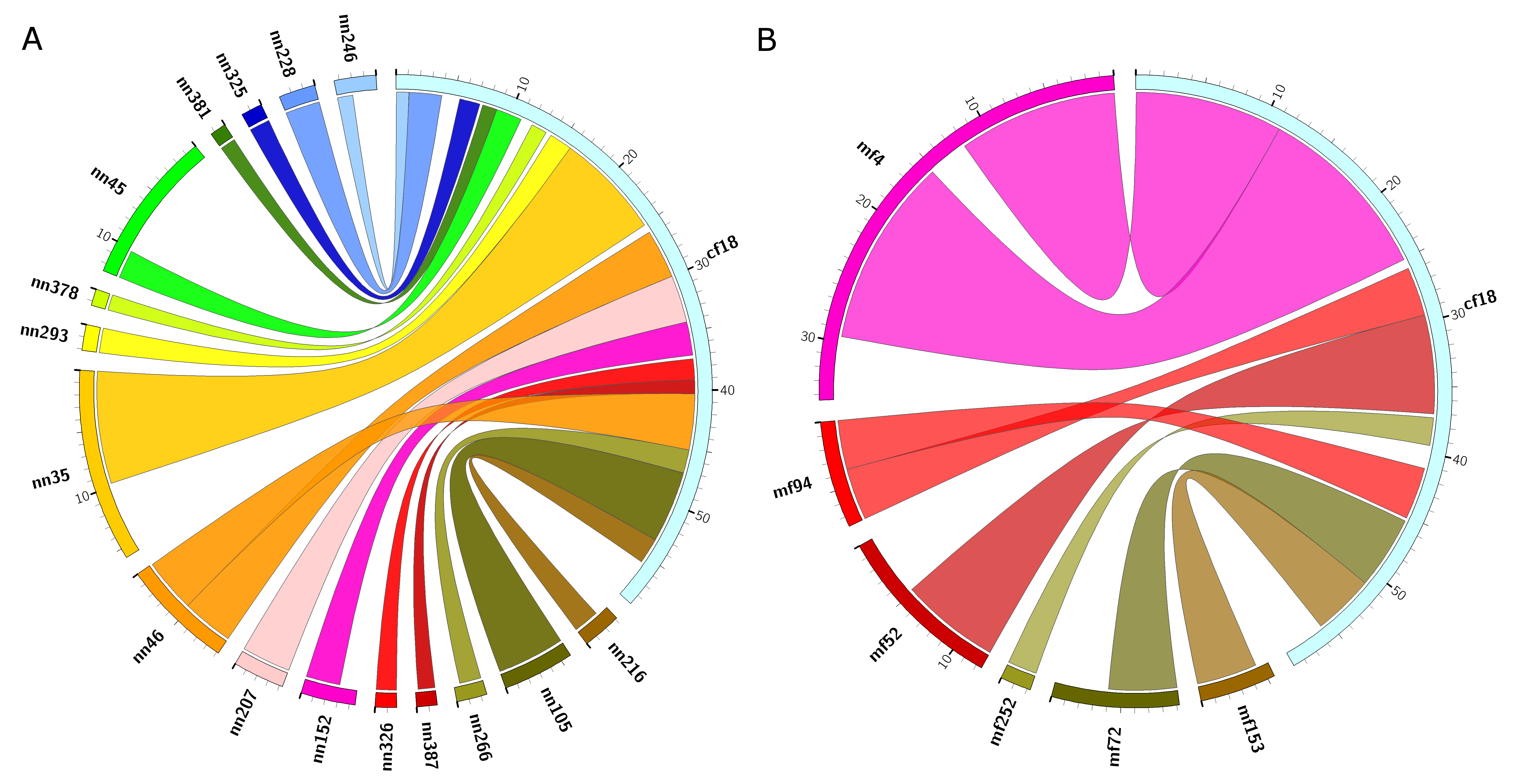


Supplementary Figure S18. Genome alignment of mink (A) and ferret (B) to dog chromosome 18 (cf18). The mink scaffold start with ’nn’ and ferret scaffold start with ’mf’. Position 0 Mb to 10 Mb of mink scaffold 35 (nn35) can be aligned to position 16 Mb to 25 Mb of dog chromosome 18 (cf18) and position 15 Mb to 30 Mb of ferret scaffold 4 (mf4) can be aligned to position 11 Mb to 25 Mb of cf18. Position 0 Mb to 3 Mb of nn152 can be aligned to positon 34 Mb to 37 Mb of cf18 and position 5 Mb to 12 Mb of mf52 can be aligned to position 29 Mb to 37 Mb of cf18. Position 0 Mb to 5 Mb of nn46 and position 0 Mb to 4 Mb of mf94 can be aligned to position 40 Mb to 45 Mb of cf18 and position 41 Mb to 45 Mb of cf18 whereas position 5 Mb to 9 Mb of nn46 and position 4 Mb to 8 Mb of mf94 can be aligned to position 26 Mb to 30 Mb of cf18.


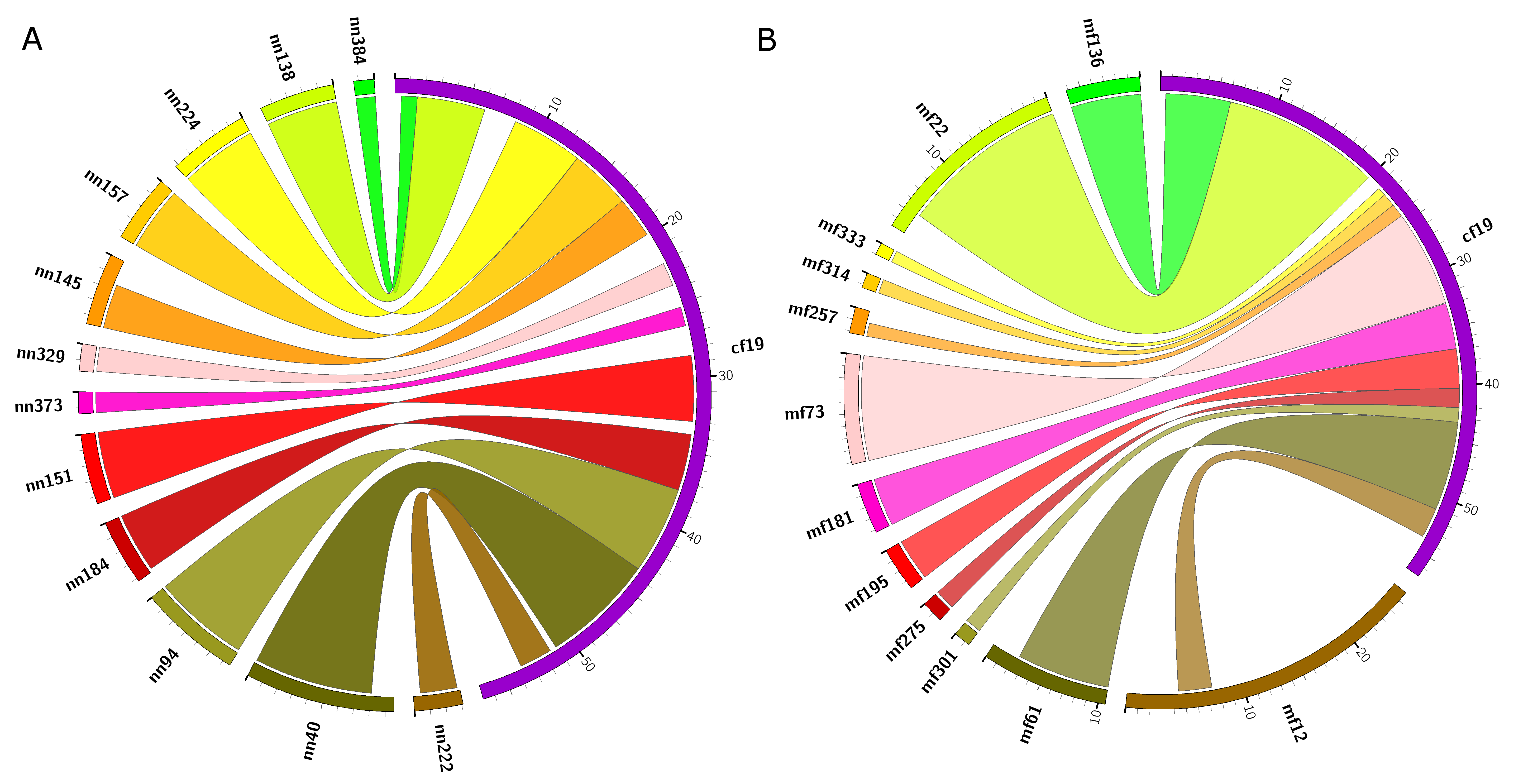


Supplementary Figure S19. Genome alignment of mink (A) and ferret (B) to dog chromosome 19 (cf`9). The mink scaffold start with ’nn’ and ferret scaffold start with ’mf’. Position 2 Mb to 5 Mb of mink scaffold 145 (nn145) can be aligned to position 17 Mb to 20 Mb of dog chromosome 19 (cf19) and position 0 Mb to 15 Mb of ferret scaffold 22 (mf22) can be aligned to position 6 Mb to 20 Mb of cf19. Position 0 Mb to 8 Mb of nn40 can be aligned to position 43 Mb to 51 Mb of cf19 and position 3 Mb to 11 Mb of mf61 can be aligned to position 43 Mb to 51 Mb of cf19.


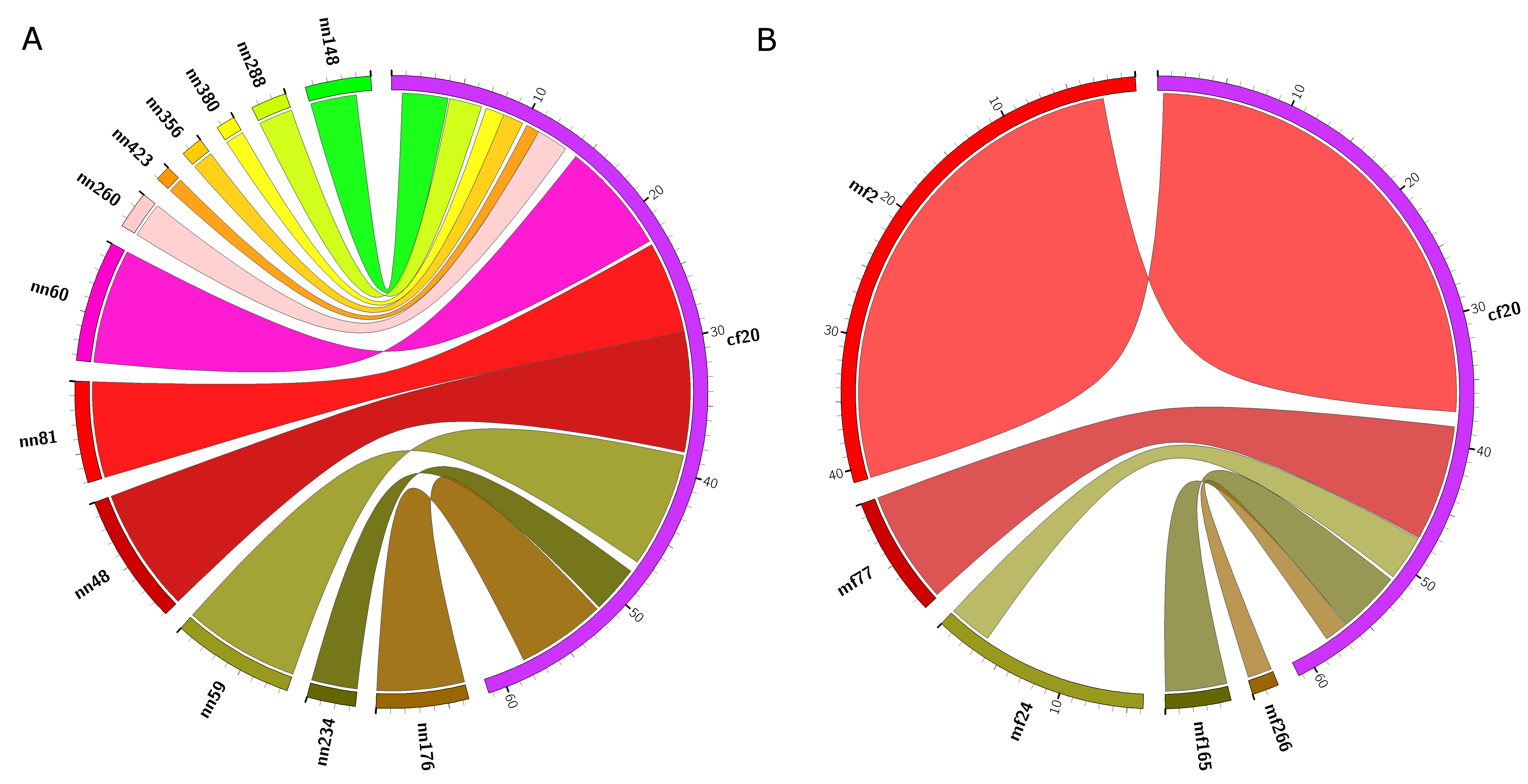


Supplementary Figure S20. Genome alignment of mink (A) and ferret (B) to dog chromosome 20 (cf20). The mink scaffold start with ’nn’ and ferret scaffold start with ’mf’.


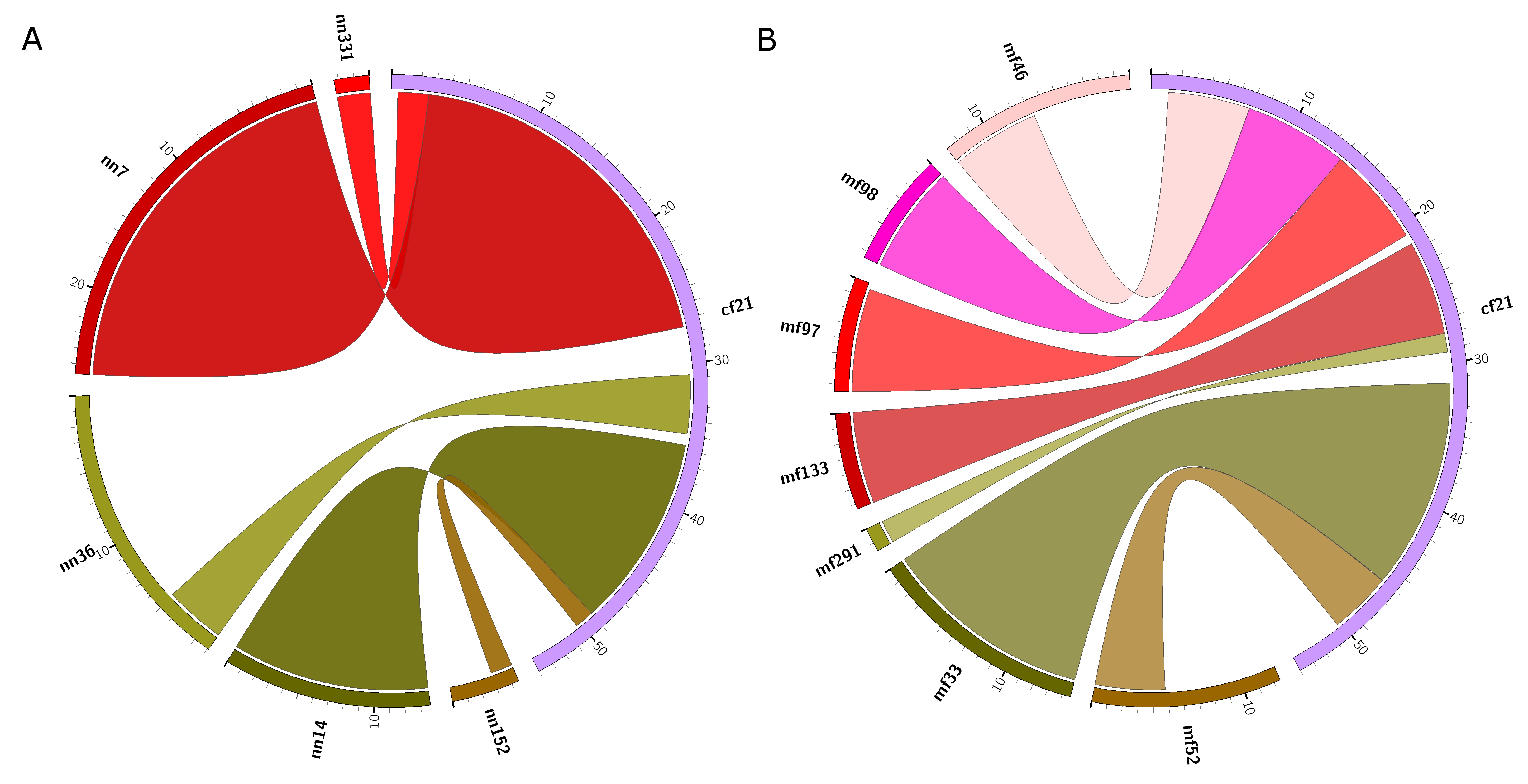


Supplementary Figure S21. Genome alignment of mink (A) and ferret (B) to dog chromosome 21 (cf21). The mink scaffold start with ’nn’ and ferret scaffold start with ’mf’. Position 3 Mb to 5 Mb of mink scaffold 152 (nn152) can be aligned to position 49 Mb to 51 Mb of dog chromosome 21 (cf21) and position 0 Mb to 5 Mb of ferret scaffold (mf52) can be aligned to position 6 Mb to 51 Mb of cf21.


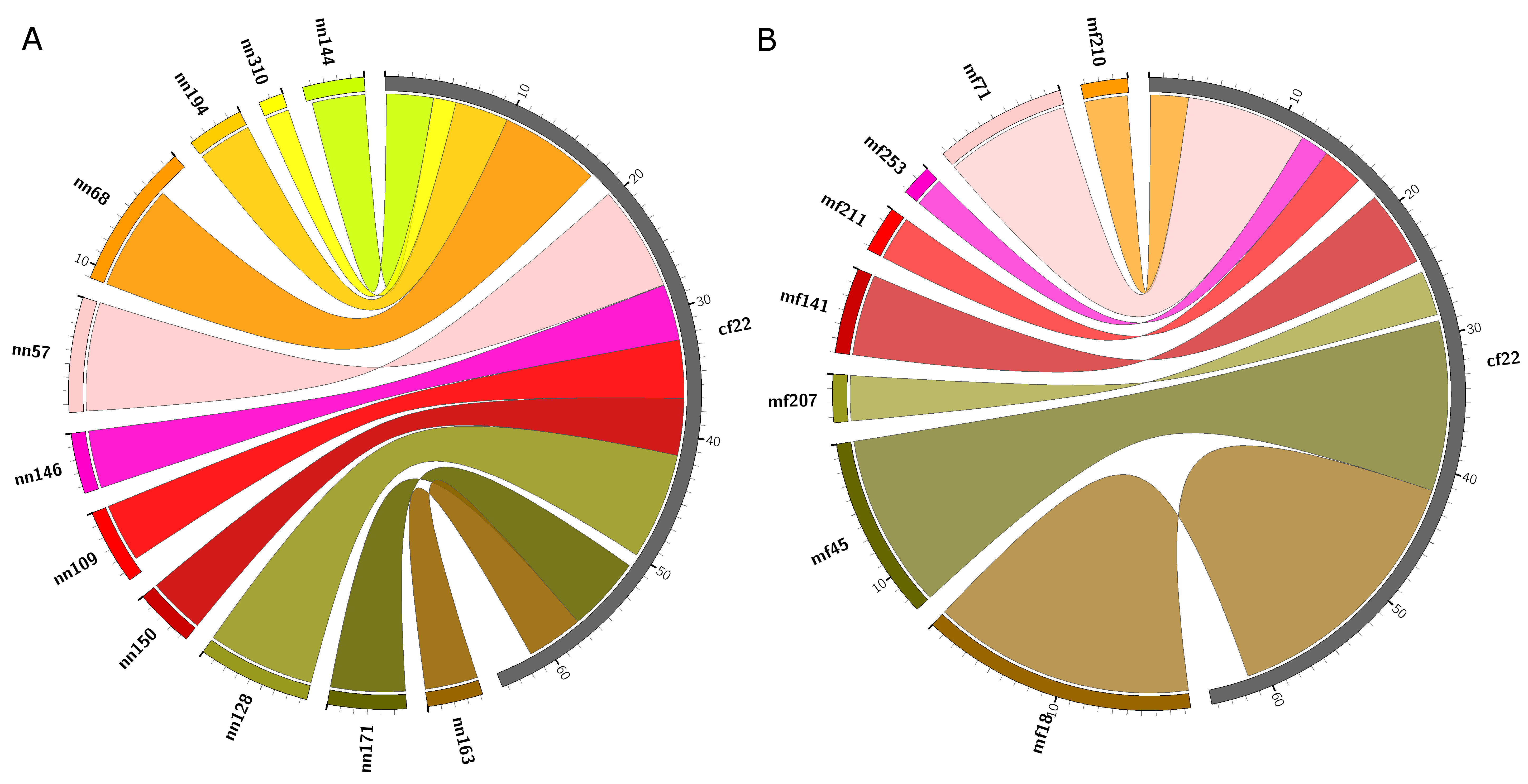


Supplementary Figure S22. Genome alignment of mink (A) and ferret (B) to dog chromosome 22 (cf22). The mink scaffold start with ’nn’ and ferret scaffold start with ’mf’.


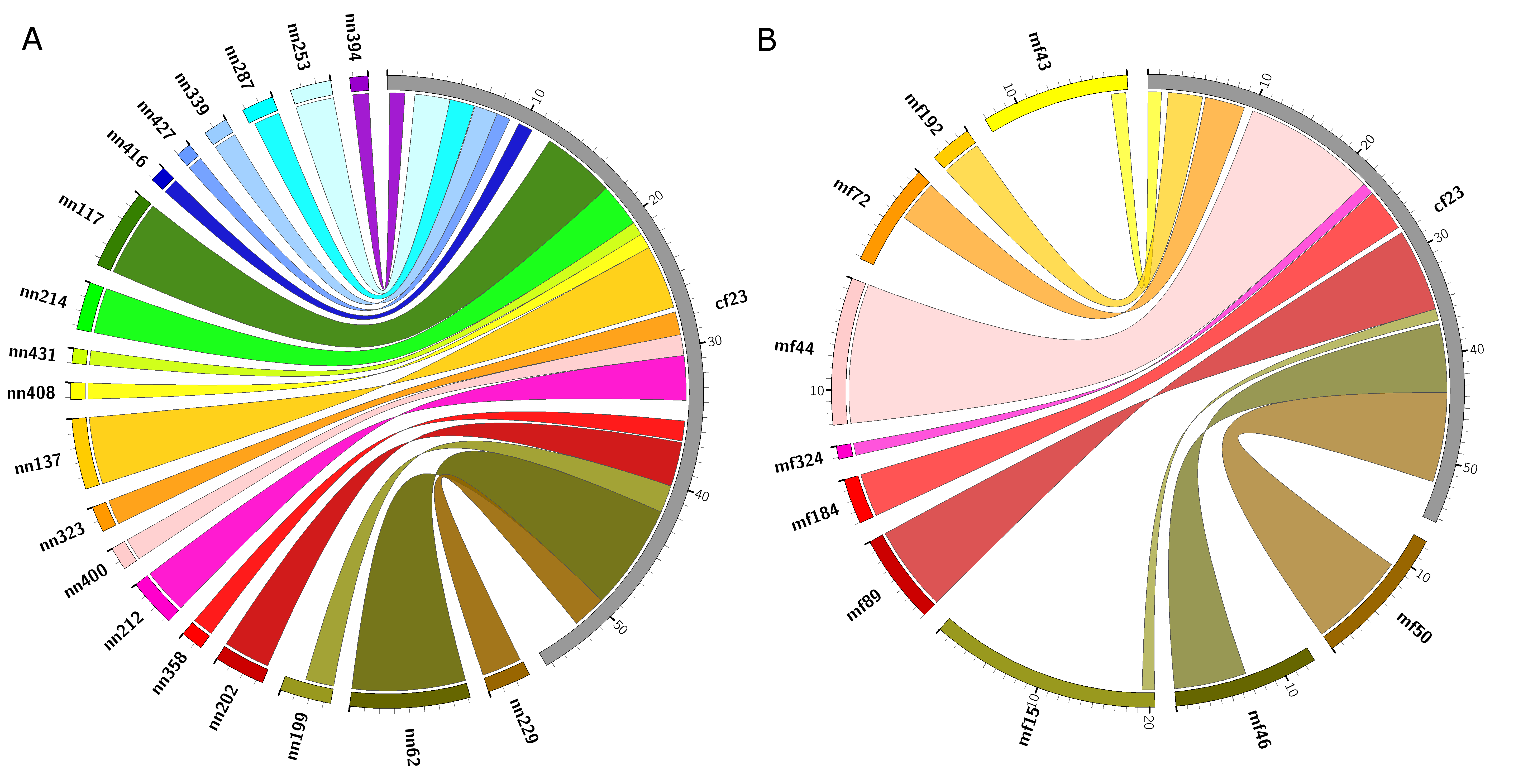


Supplementary Figure S23. Genome alignment of mink (A) and ferret (B) to dog chromosome 23 (cf23). The mink scaffold start with ’nn’ and ferret scaffold start with ’mf’.


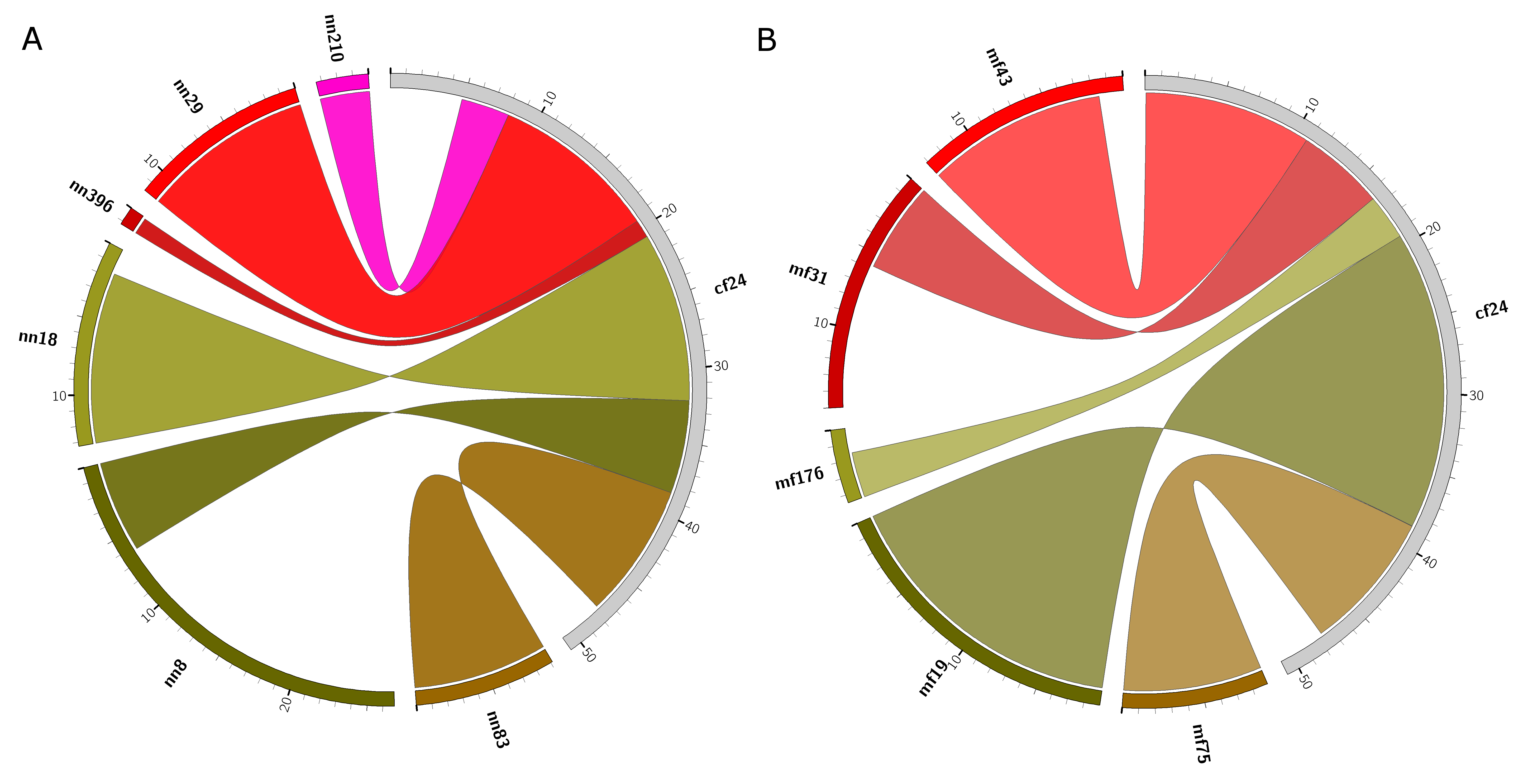


Supplementary Figure S24. Genome alignment of mink (A) and ferret (B) to dog chromosome 24 (cf24). The mink scaffold start with ’nn’ and ferret scaffold start with ’mf’.


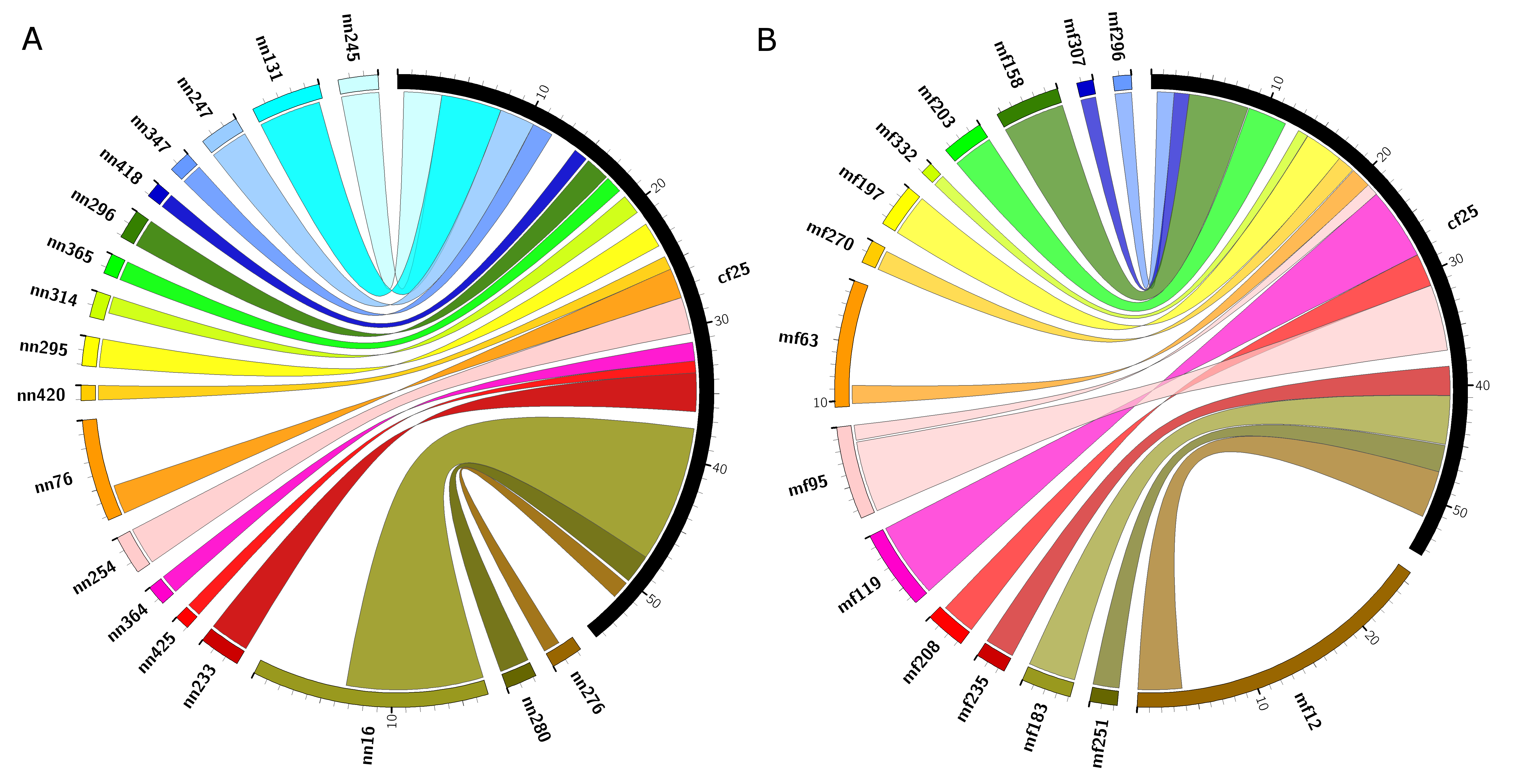


Supplementary Figure S25. Genome alignment of mink (A) and ferret (B) to dog chromosome 25 (cf25). The mink scaffold start with ’nn’ and ferret scaffold start with ’mf’.


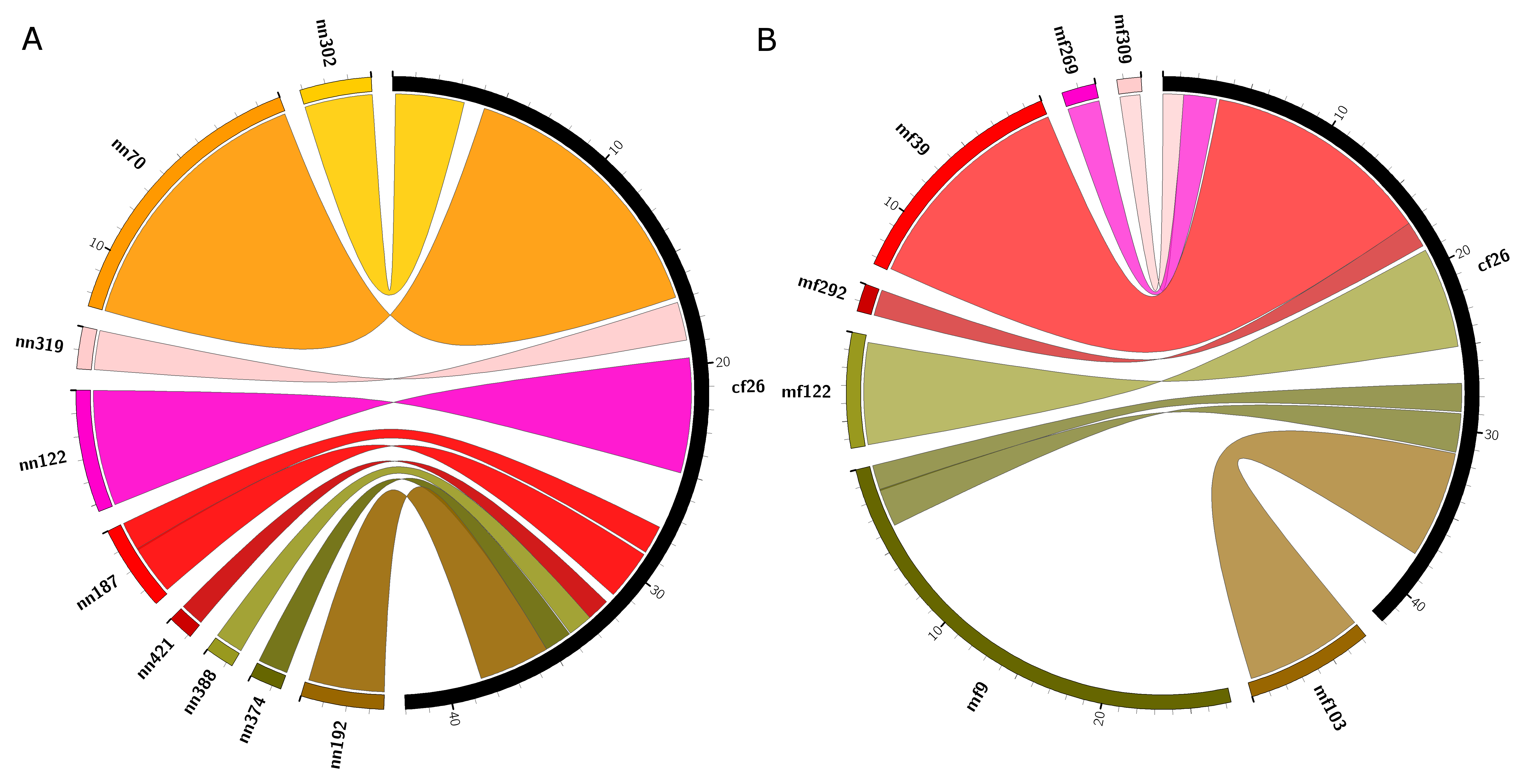


Supplementary Figure S26. Genome alignment of mink (A) and ferret (B) to dog chromosome 26 (cf26). The mink scaffold start with ’nn’ and ferret scaffold start with ’mf’. Position 0 Mb to 1 Mb of mink scaffold 187 (nn187) and position 0 Mb to 1 Mb of ferret scaffold 9 (mf9) can be aligned to position 27 Mb to 28 Mb of dog chromosome 26 (cf26) whereas position 1 Mb to 3 Mb of nn187 and mf9 can be aligned to the reverse sequence of position 29 Mb to 31 Mb of cf26.


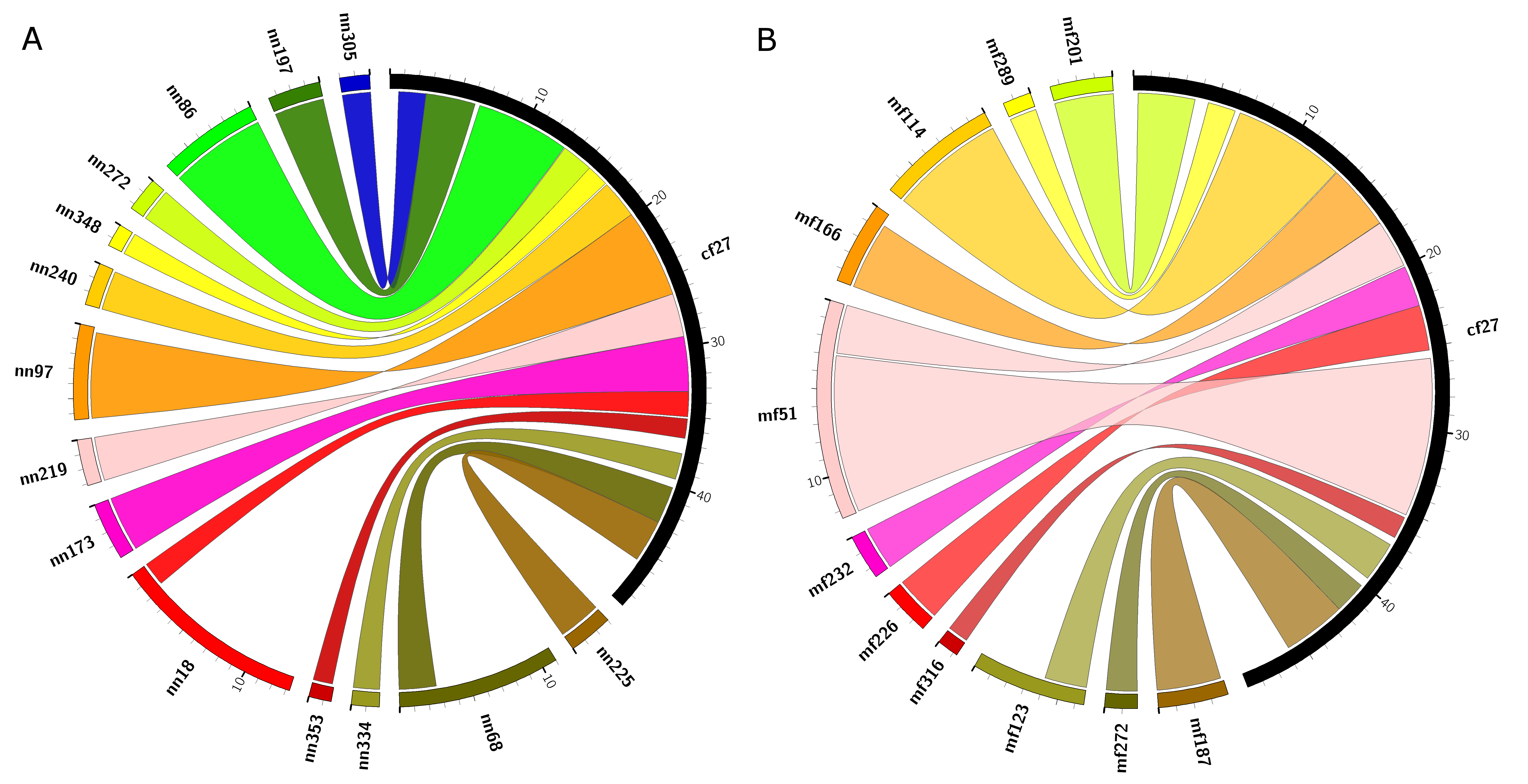


Supplementary Figure S27. Genome alignment of mink (A) and ferret (B) to dog chromosome 27 (cf27). The mink scaffold start with ’nn’ and ferret scaffold start with ’mf’.


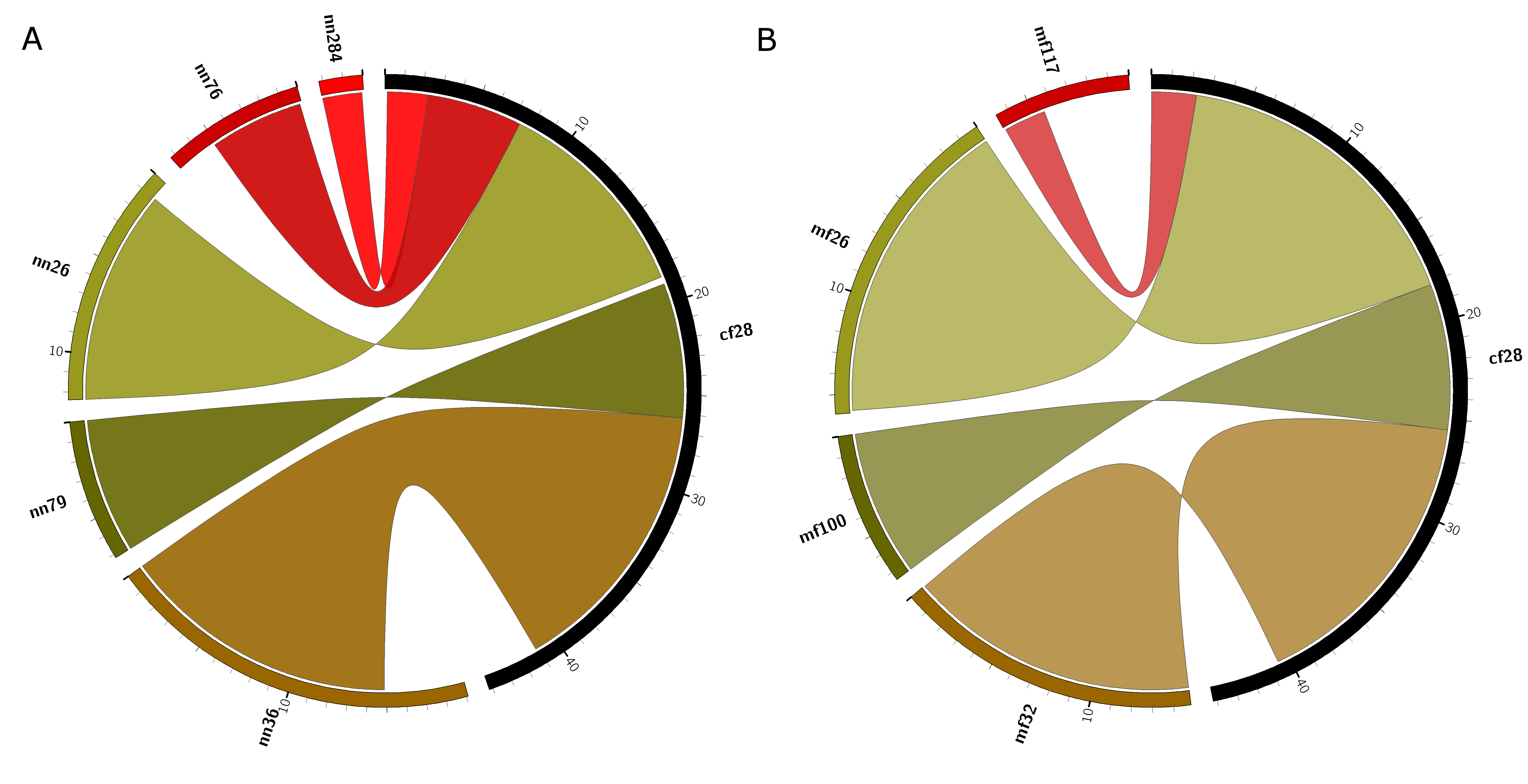


Supplementary Figure S28. Genome alignment of mink (A) and ferret (B) to dog chromosome 28 (cf28). The mink scaffold start with ’nn’ and ferret scaffold start with ’mf’.


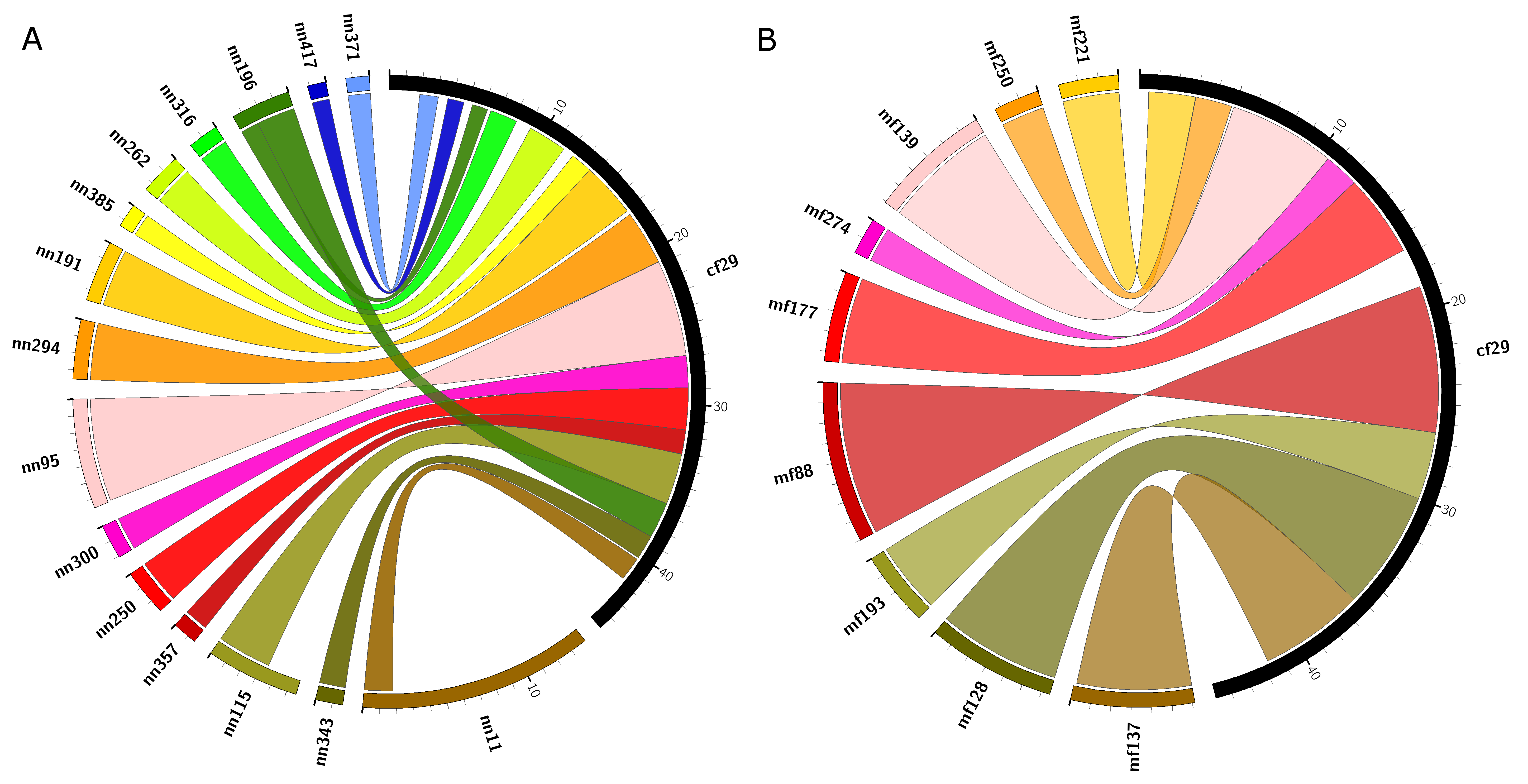


Supplementary Figure S29. Genome alignment of mink (A) and ferret (B) to dog chromosome 29 (cf29). The mink scaffold start with ’nn’ and ferret scaffold start with ’mf’.


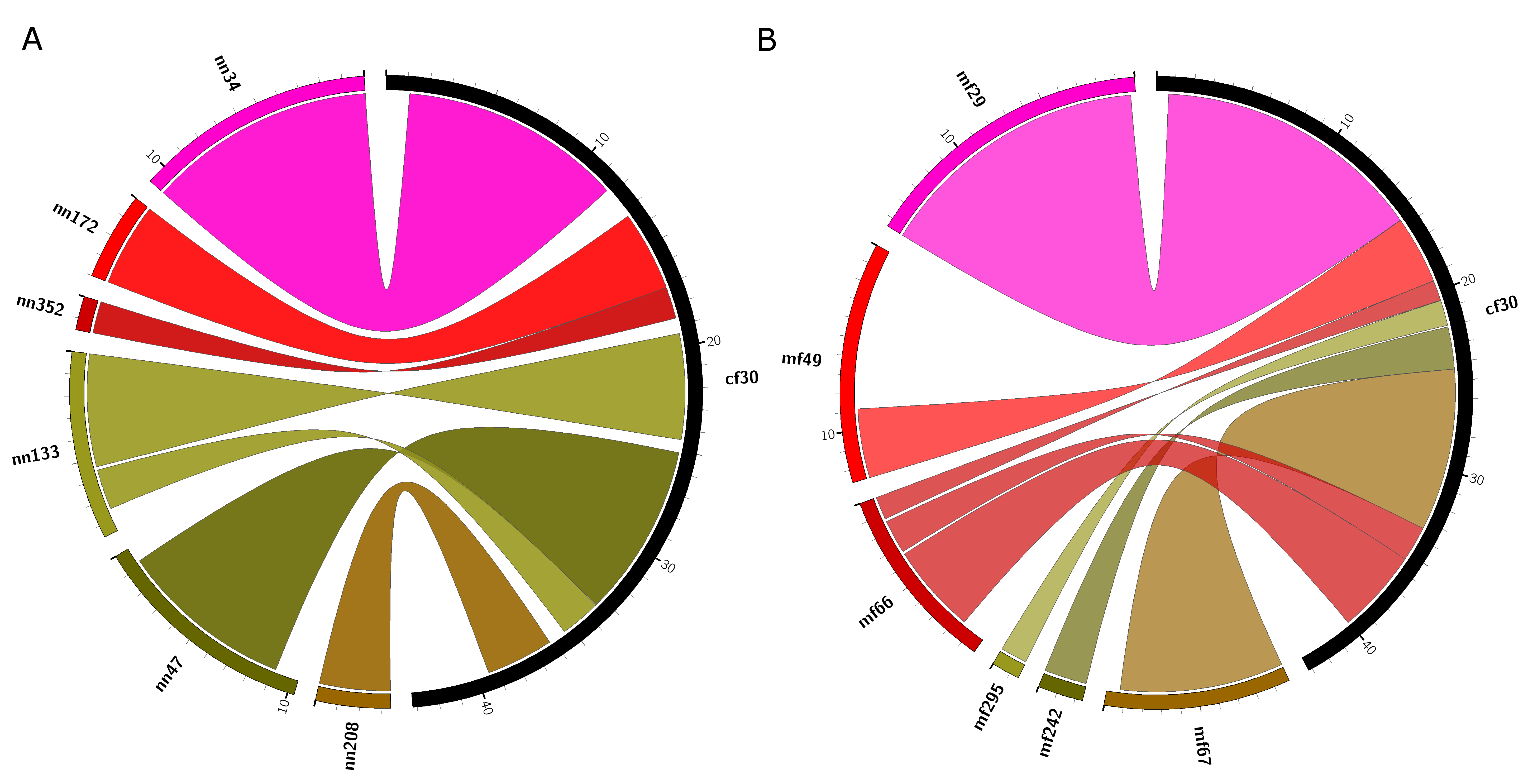


Supplementary Figure S30. Genome alignment of mink (A) and ferret (B) to dog chromosome 30 (cf30). The mink scaffold start with ’nn’ and ferret scaffold start with ’mf’.


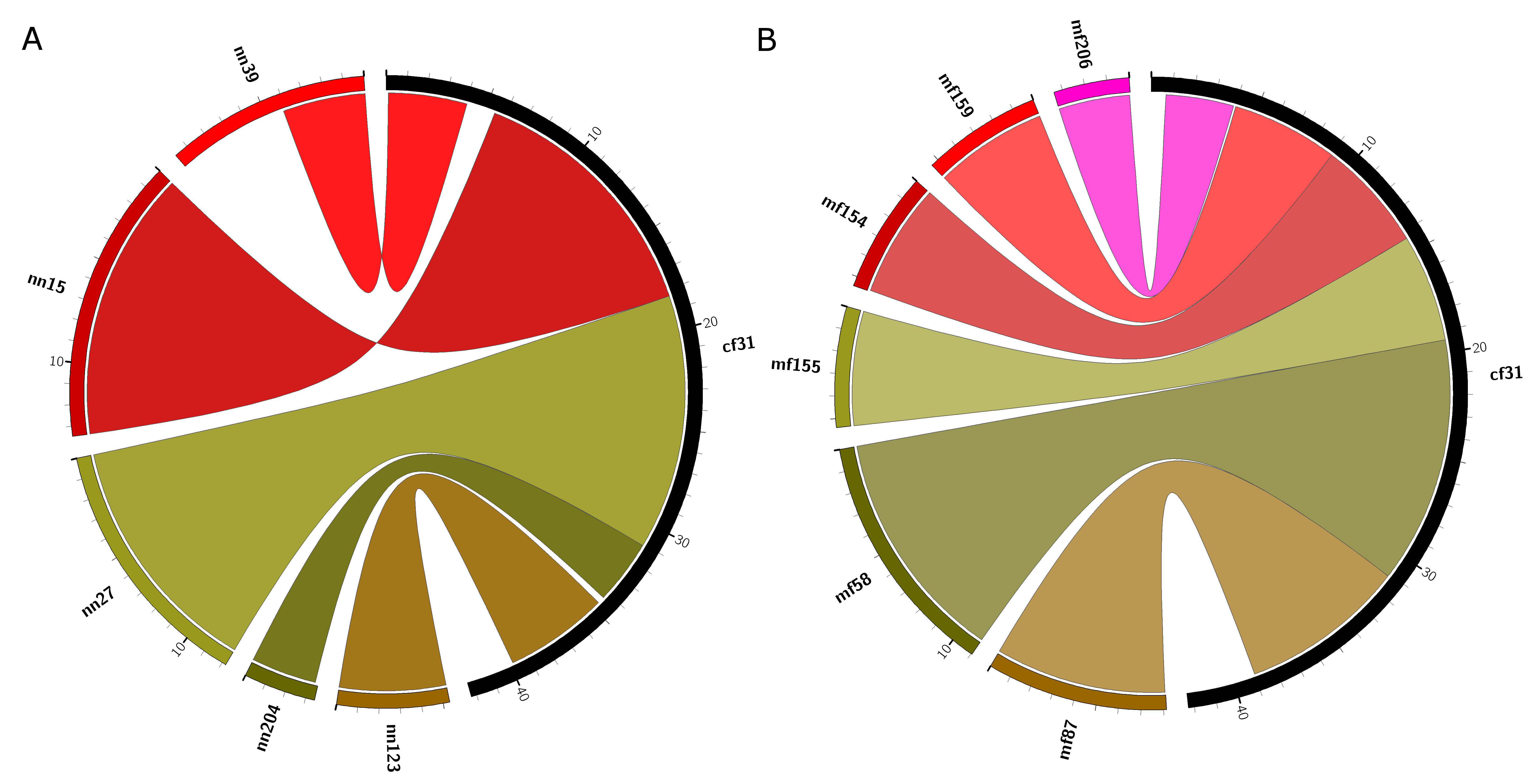


Supplementary Figure S31. Genome alignment of mink (A) and ferret (B) to dog chromosome 31 (cf31). The mink scaffold start with ’nn’ and ferret scaffold start with ’mf’.


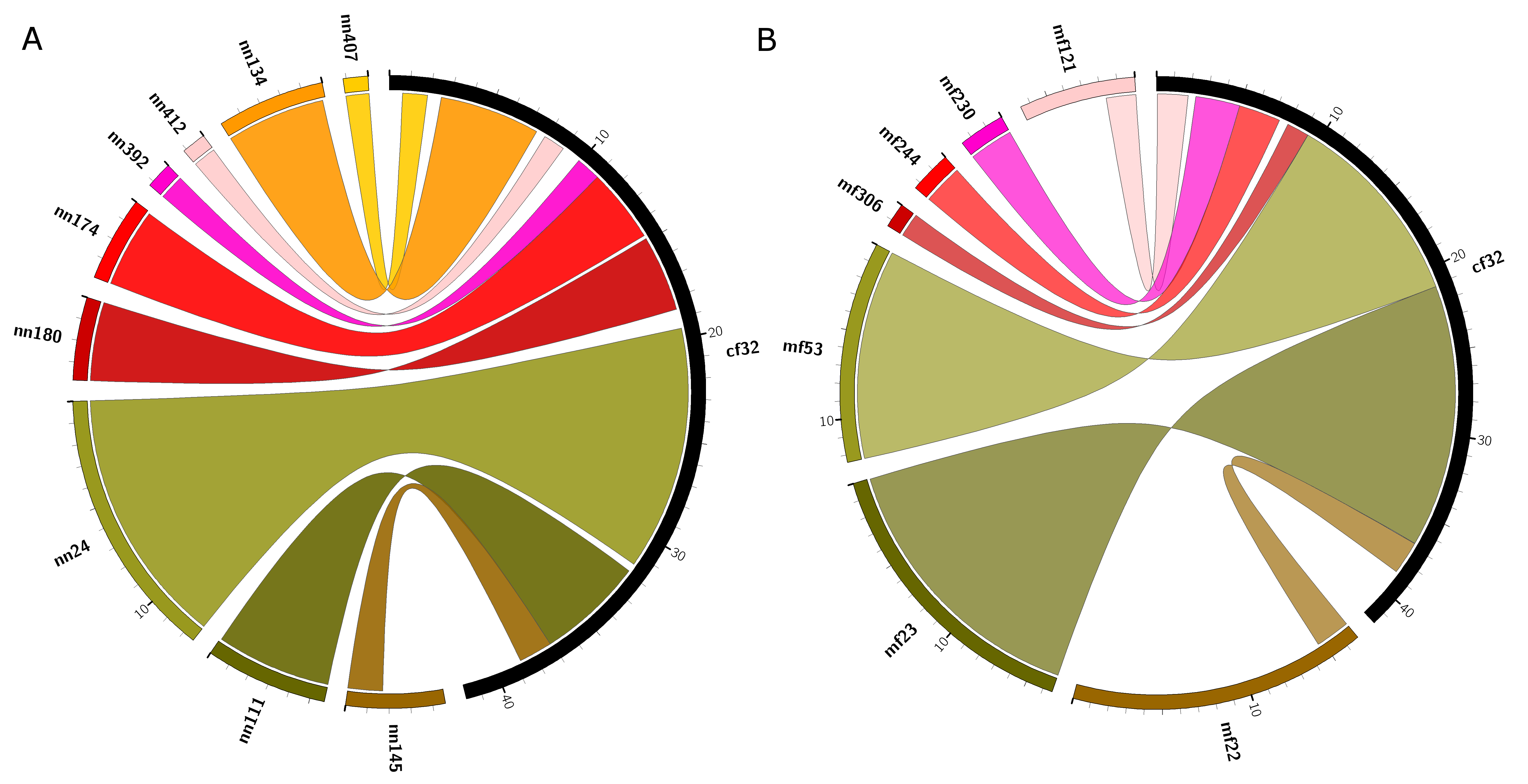


Supplementary Figure S32. Genome alignment of mink (A) and ferret (B) to dog chromosome 32 (cf32). The mink scaffold start with ’nn’ and ferret scaffold start with ’mf’. Position 0 Mb to 2 Mb of mink scaffold 145 (nn145) can be aligned to position 37 Mb to 39 Mb of dog chromosome 32 (cf32) and position 15 Mb to 17 Mb of ferret scaffold 22 (mf22) can be aligned to position 37 Mb to 39 Mb of cf32.


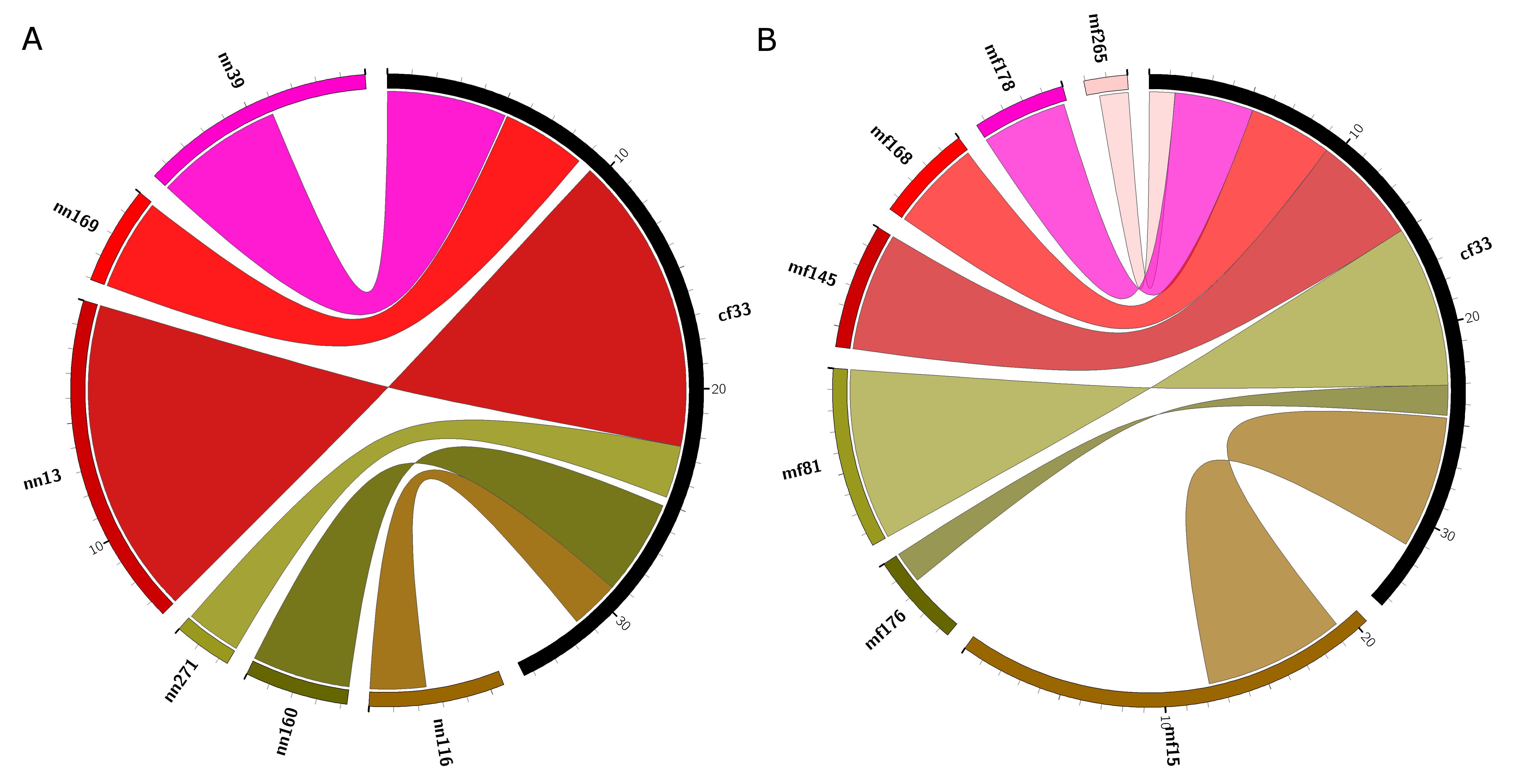


Supplementary Figure S33. Genome alignment of mink (A) and ferret (B) to dog chromosome 33 (cf33). The mink scaffold start with ’nn’ and ferret scaffold start with ’mf’.


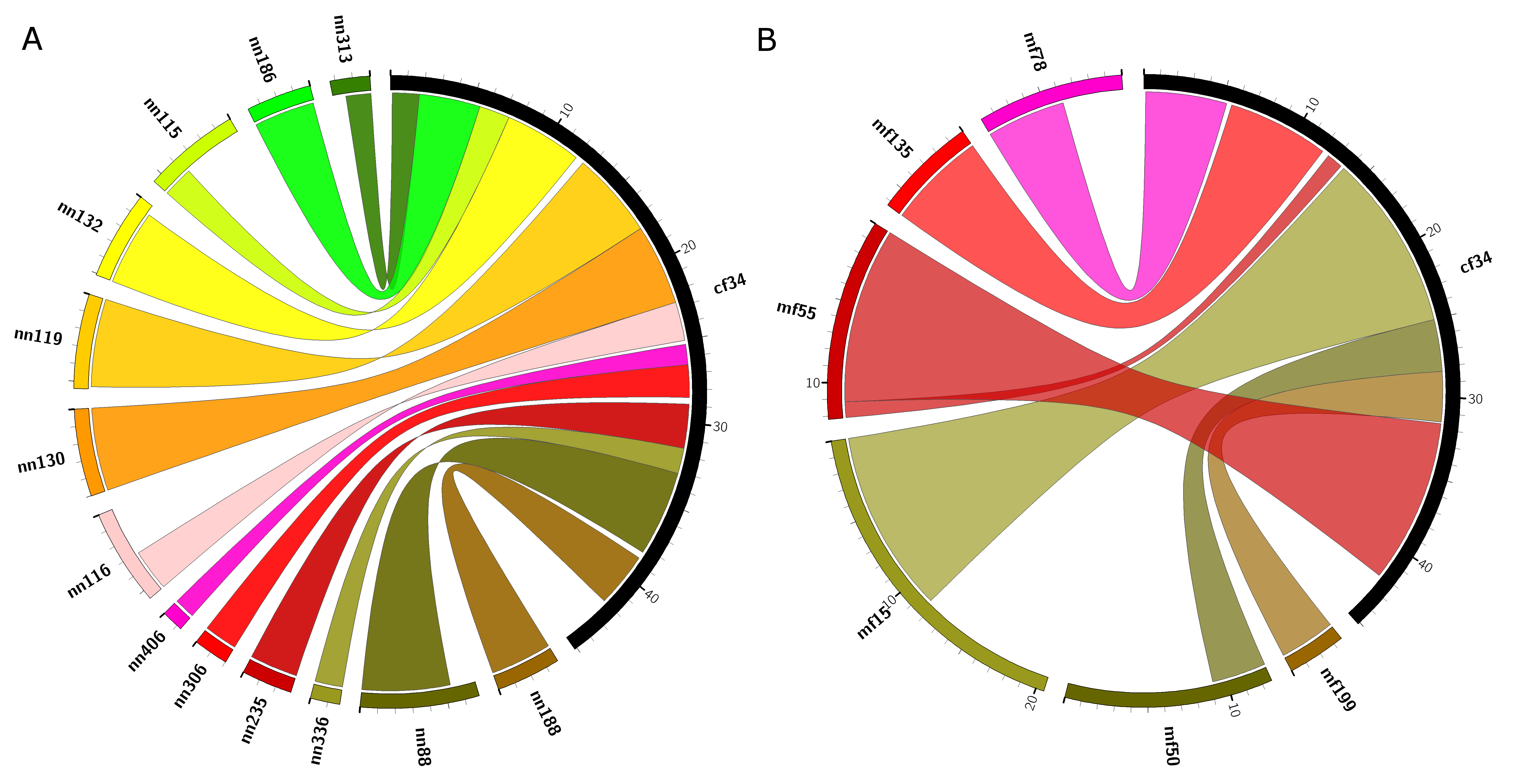


Supplementary Figure S34. Genome alignment of mink (A) and ferret (B) to dog chromosome 34 (cf34). The mink scaffold start with ’nn’ and ferret scaffold start with ’mf’.


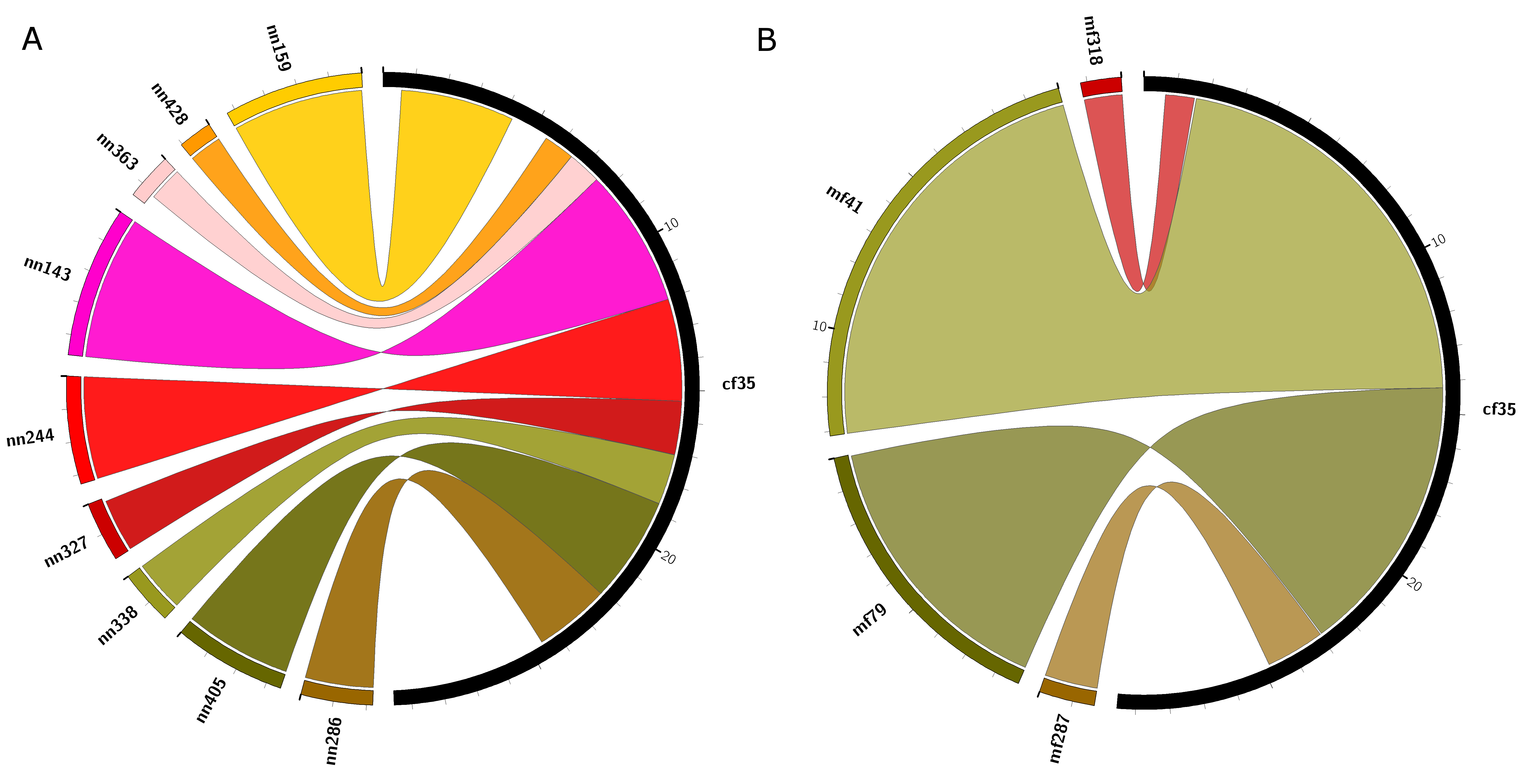


Supplementary Figure S35. Genome alignment of mink (A) and ferret (B) to dog chromosome 35 (cf35). The mink scaffold start with ’nn’ and ferret scaffold start with ’mf’.


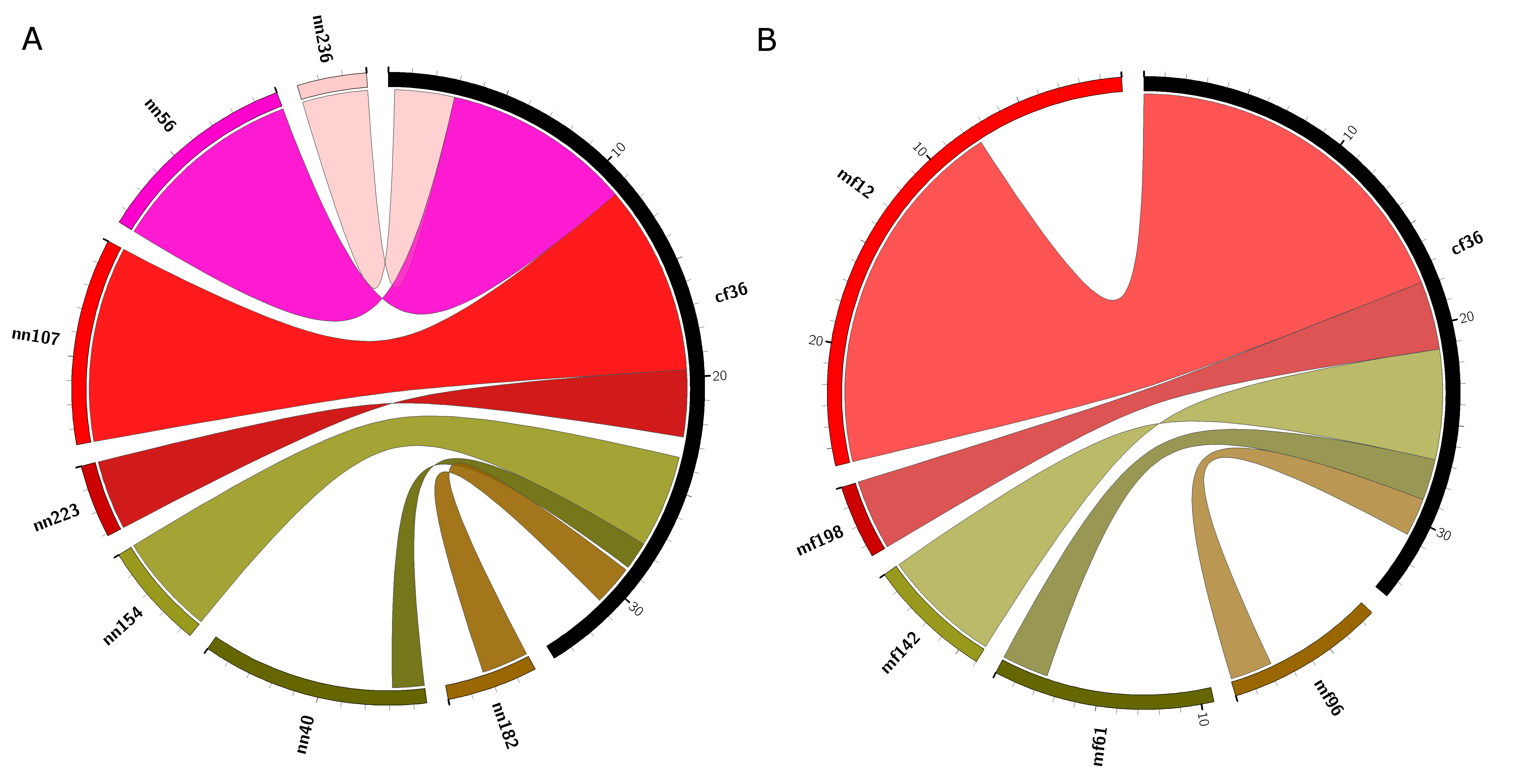


Supplementary Figure S36. Genome alignment of mink (A) and ferret (B) to dog chromosome 36 (cf36). The mink scaffold start with ’nn’ and ferret scaffold start with ’mf’. Position 8 Mb to 10 Mb of mink scaffold 40 (nn40) can be aligned to position 27 Mb to 29 Mb of dog chromosome 36 (cf36) and position 0 Mb to 2 Mb of ferret scaffold 61 (mf61) can be aligned to position 37 Mb to 39 Mb of cf36. Position 2 Mb to 4 Mb of nn182 can be aligned to position 29 Mb to 31 Mb of cf36 and position 0 Mb to 2 Mb of mf96 can be aligned to position 29 Mb to 31 Mb of cf36.


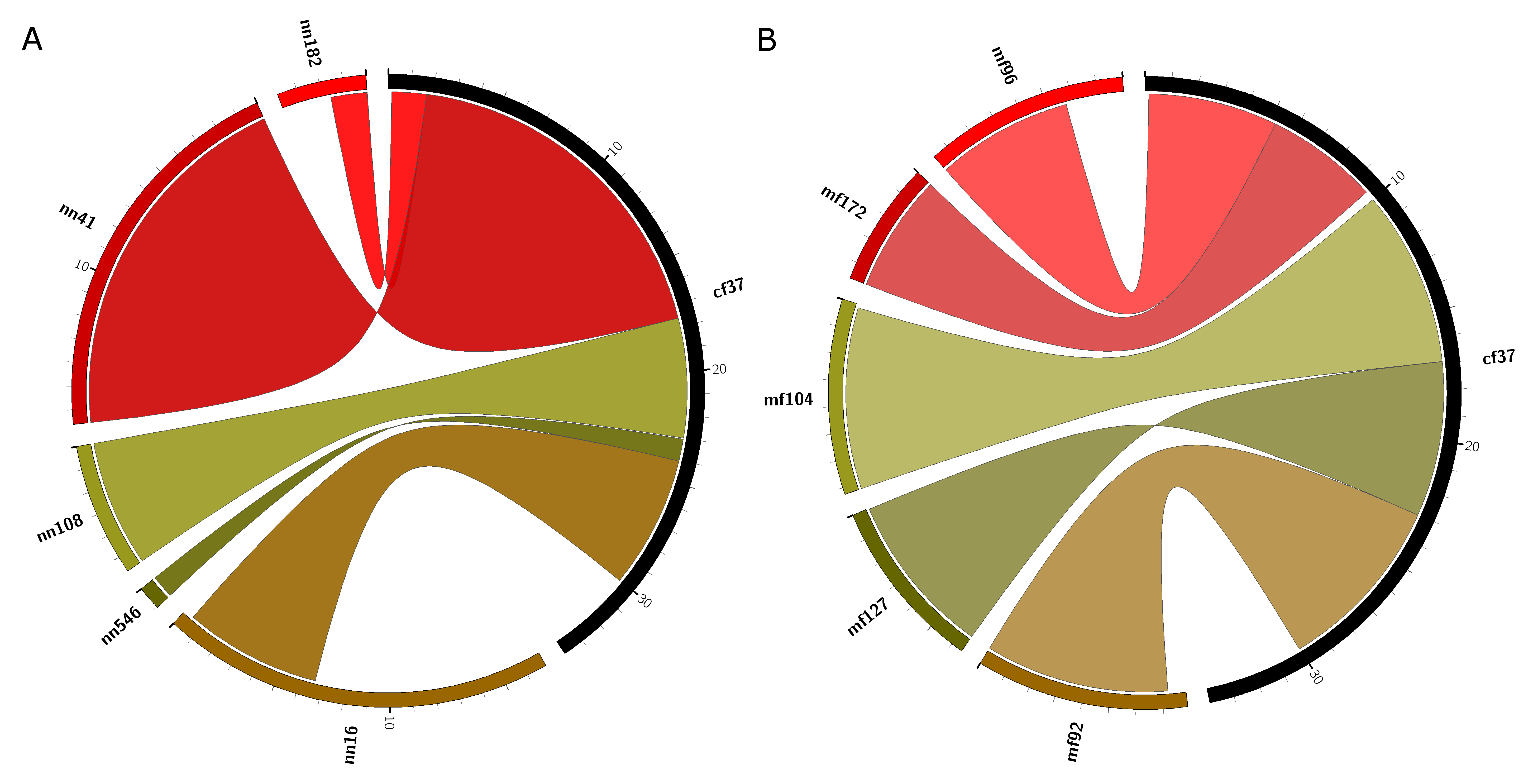


Supplementary Figure S37. Genome alignment of mink (A) and ferret (B) to dog chromosome 37 (cf37). The mink scaffold start with ’nn’ and ferret scaffold start with ’mf’. Position 0 Mb to 2 Mb of mink scaffold 182 (nn182) can be aligned to position 0 Mb to 2 Mb of cf37 and position 2 Mb to 7 Mb of mf96 can be aligned to position 0 Mb to 5 Mb of cf37.


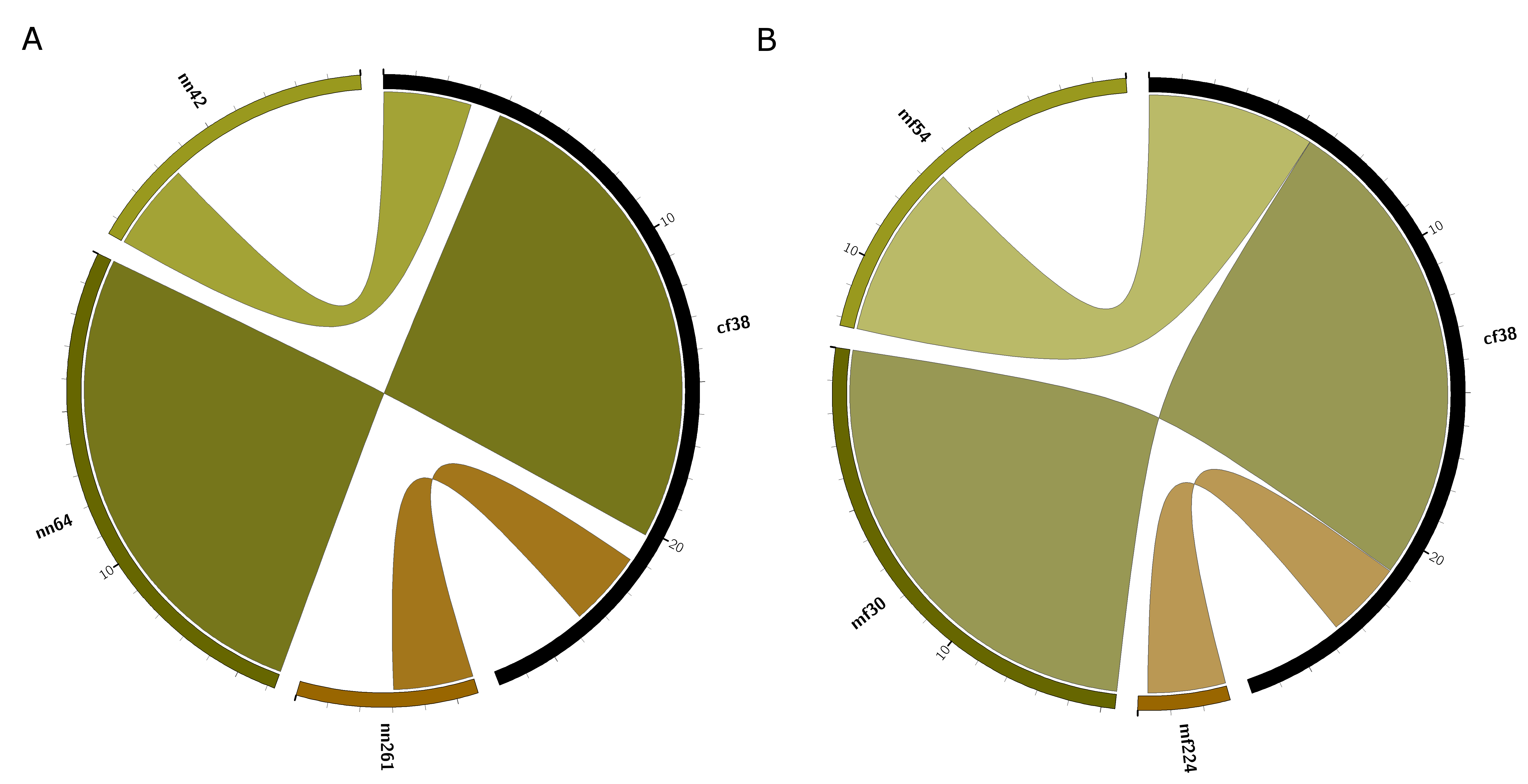


Supplementary Figure S38. Genome alignment of mink (A) and ferret (B) to dog chromosome 38 (cf38). The mink scaffold start with ’nn’ and ferret scaffold start with ’mf’.


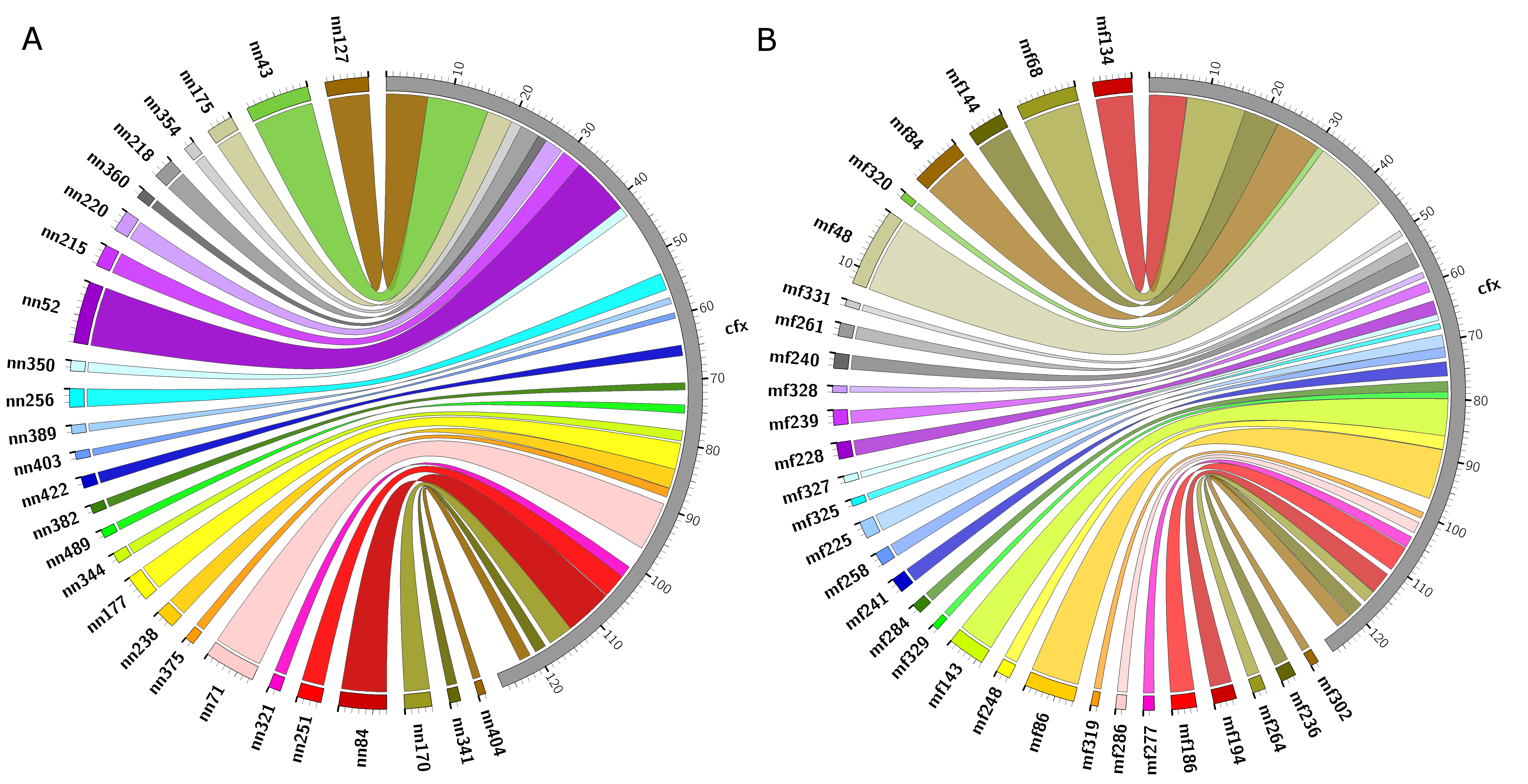


Supplementary Figure S39. Genome alignment of mink (A) and ferret (B) to dog chromosome X (cfx). The mink scaffold start with ’nn’ and ferret scaffold start with ’mf’.

Supplementary Table S7. Location of the SSR from linkage map

| Marker | Location (cm) | Location |
| --- | --- | --- |
| Mvi6056 | Lg1:0 | Scaffold30: 17015193 |
| RAN83 | Lg1:10 | Scaffold601: 256820 |
| Mvi4079 | Lg1:15 | Scaffold259: 2360634 |
| Mvi2621 | Lg1:22 | Scaffold164: 338810 |
| Mvi6028 | Lg1:26 | Scaffold143: 2646092 |
| Mvi3615 | Lg1:71 | Scaffold279: 1856033 |
| Mvi1951 | Lg1:79 | Scaffold278: 653643 |
| Mvi1901 | Lg1:82 | Scaffold17: 4883507 |
| Lut733 | Lg1:97 | Scaffold20: 5512963 |
| Mvi4076 | Lg1:123 | Scaffold186: 1486716 |
| Mvi4075 | Lg2: 0 | Scaffold10: 7115546 |
| Mvi4010 | Lg2: 19 | Scaffold10: 11354456 |
| Mvi6045 | Lg2: 23 | Scaffold10: 12374722 |
| RAN77 | Lg2: 54 | Scaffold309: 312552 |
| Mvi1843 | Lg2: 58 | Scaffold192: 1648086 |
| Mvi4037 | Lg2: 59 | Scaffold125: 3294052 |
| Mvi2407 | Lg2: 142 | Scaffold79: 6517996 |
| Mvi6096 | Lg3: 0 | Scaffold2: 8925515 |
| Mvi4094 | Lg3: 5 | Scaffold49: 10038886 |
| Mvi1007 | Lg3: 11 | Scaffold70: 12289229 |
| Mvi1914 | Lg3: 24 | Scaffold107: 2132593 |
| RAN79 | Lg3: 30 | Scaffold280: 294769 |
| Mvi111 | Lg3: 40 | Scaffold108: 81716 |
| RAN95 | Lg3: 80 | Scaffold32: 129794 |
| RAN35 | Lg4: 0 | Scaffold153: 891748 |
| Mvi3603 | Lg4: 22 | Scaffold11: 4532101 |
| RAN87 | Lg4: 27 | Scaffold11: 10572611 |
| Mvi2627 | Lg4: 40 | Scaffold602: 86708 |
| Mvi1929 | Lg4: 71 | Scaffold25: 9120956 |
| Mvi6060 | Lg5: 0 | Scaffold128: 4034432 |
| Mvi1538 | Lg5: 40 | Scaffold28: 1126486 |
| Mvi2606 | Lg6: 4 | Scaffold48: 3987444 |
| Mvi6066 | Lg6: 12 | Scaffold81: 4248469 |
| RAN98 | Lg6: 19 | Scaffold60: 678476 |
| Mvi4063 | Lg6: 22 | Scaffold60: 6695051 |
| Mvi4042 | Lg6: 28 | Scaffold260: 1530475 |
| Mvi4052 | Lg6: 39 | Scaffold188: 3324538 |
| RAN59 | Lg6: 44 | Scaffold235: 119358 |
| Lut604 | Lg6: 50 | Scaffold13: 4879513 |
| Mvi2243 | Lg6: 53 | Scaffold13: 12677683 |
| RAN115 | Lg7: 0 | Scaffold3: 6450200 |
| Mvi1957 | Lg7: 33 | Scaffold3: 23998455 |
| Mvi1614 | Lg7: 48 | Scaffold36: 17604590 |
| Mvi4062 | Lg8: 0 | Scaffold83: 4432322 |
| Mvi4025 | Lg8: 41 | Scaffold18: 6915051 |
| RAN92 | Lg8: 80 | Scaffold8: 7525080 |
| RAN105 | Lg9: 0 | Scaffold172: 1079238 |
| Mvi6029 | Lg9: 39 | Scaffold80: 4610175 |
| Mvi6061 | Lg9: 82 | Scaffold63: 4501456 |
| RAN30 | Lg9: 89 | Scaffold118: 9039106 |
| RAN31 | Lg9: 104 | Scaffold118: 8806836 |
| Mvi4082 | Lg10: 0 | Scaffold64: 8856428 |
| Mvi4040 | Lg10: 15 | Scaffold64: 6007323 |
| Mvi3618 | Lg11: 40 | Scaffold167: 2285606 |
| RAN108 | Lg11: 49 | Scaffold180: 1805700 |
| Mvi6098 | Lg11: 55 | Scaffold420: 730658 |
| RAN10 | Lg11: 73 | Scaffold281: 75532 |
| RAN101 | Lg12: 0 | Scaffold226: 2828875 |
| RAN73 | Lg12: 6 | Scaffold77: 161769 |
| RAN63 | Lg12: 17 | Scaffold23: 4276738 |
| RAN38 | Lg12: 28 | Scaffold23: 5492267 |
| Mvi6017 | Lg12: 37 | Scaffold23: 9088448 |
| RAN71 | Lg12: 54 | Scaffold77: 305507 |
| Mvi6094 | Lg12: 94 | Scaffold106: 6765606 |
| Mvi4091 | Lg13: 26 | Scaffold1: 29506814 |
| Mvi4041 | Lg13: 62 | Scaffold78: 1502865 |
| RAN26 | Lg13: 82 | Scaffold85: 4784254 |
| Mer22 | Lg14: 0 | Scaffold92: 5791585 |
| Mvi1321 | Lg14: 9 | Scaffold114: 2215172 |

Supplementary Table S8. Location of genes which may be involved in fur quality and fur color

|  | Location | Note* |
| --- | --- | --- |
| ASIP(A) | Scaffold18: 10561641-10567254 | Distribution of pigment |
| MITF(S) | Scaffold60: 525579 - 744253 | degree and distribution of spotting |
| PMEL(M) | Scaffold77: 305758 - 314440 | merle pattern |
| MC1R(E) | Scaffold99: 6234528 - 6235478 | determining expression of eumelanin |
| FGF5(L) | Scaffold134: 2968173 - 2988731 | length of hair |
| DEFB103(K) | Scaffold156: 314928 - 316012 | coloring pattern |
| KRT71(R) | Scaffold197: 17127 – 214955  Scaffold305: 34110 – 42668  Scaffold305: 55231 – 101385  Scaffold305: 89742 – 242082  Scaffold305: 234140 - 304386 | Determine whether an animal's coat is straight or curly |
| TYRP1(B) | Scaffold297: 55231 - 73321 | determine the degree to which an animal expresses tyrosinase |
| KIT | Scaffold37: 6179515 - 6259242 | regulation of cell survival and proliferation, hematopoiesis, stem cell maintenance, gametogenesis, mast cell development, migration and function, and in melanogenesis |
| LYST | Scaffold309: 355504 - 500120 | May be required for sorting endosomal resident proteins into late multivesicular endosomes by a mechanism involving microtubules. |
| AGRP | Scaffold3: 6402931 - 6403706 | Involved in the control of feeding behavior through the central melanocortin system |
| Atoh-1 | Scaffold180: 1833037 - 1834098 | Plays a role in the differentiation of subsets of neural cells |
| ITGB1 | Scaffold226: 2855977 - 2882938 | Plays a mechanistic adhesive role during telophase, required for the successful completion of cytokinesis |
| RSPO2 | Scaffold11: 10428730 - 10583479 | Involve in Wnt signaling pathway and Probably also acts as a ligand for frizzled and LRP receptors. |
| SLC24A5 | Scaffold172: 1116489 - 1144984 | Cation exchanger involved in pigmentation, possibly by participating in ion transport in melanosomes. |
| DEFB1 | Scaffold156: 213584 - 213727 | Has bactericidal activity. |
| TMIE | Scaffold59: 5078153 - 5085613 | The protein may play some role in a cellular membrane location. |
| TYR | Scaffold7: 17055635 - 17165565 | A copper-containing oxidase that functions in the formation of pigments such as melanins and other polyphenolic compounds. |
| HLADRB1 | Scaffold601: 231858 - 232112  Scaffold601: 140294 - 140533  Scaffold601: 29252 - 29491  Scaffold624: 29507 - 29749 | NA |
| MC3R | Scaffold6: 15586560 - 15587408  Scaffold32: 84975 – 85829  Scaffold83: 6136107 - 6137075 | Receptor for MSH (alpha, beta and gamma) and ACTH. |
| MC2R | Scaffold32: 33340 - 34227 | Plays a central role in the control of energy homeostasis and body weight regulation by increasing ligand-sensitivity of MC4R and MC4R-mediated generation of cAMP (By similarity). |

*Genes function from Uniprot (http://www.uniprot.org/)

1 Li, H. Aligning sequence reads, clone sequences and assembly contigs with BWA-MEM. *arXiv preprint arXiv:1303.3997* (2013).

2 Li, H. *et al.* The sequence alignment/map format and SAMtools. *Bioinformatics* **25**, 2078-2079 (2009).

3 Novák, P., Neumann, P., Pech, J., Steinhaisl, J. & Macas, J. RepeatExplorer: a Galaxy-based web server for genome-wide characterization of eukaryotic repetitive elements from next-generation sequence reads. *Bioinformatics* **29**, 792-793 (2013).

4 Smit, A. & Hubley, R. RepeatModeler Open-1.0. *Repeat Masker Website* (2010).
